# OTUD4 stabilizes CYLD and counteracts BCL3 K63-linked polyubiquitination to suppress melanoma progression

**DOI:** 10.1038/s41419-026-09130-0

**Published:** 2026-07-28

**Authors:** Yuming He, Lin Miao, Yunfang Deng, Yuyao Xue, Zhaohui Liu, Zhiming Sun, Chujing He, Hao Feng, Jiabing Li, Yuqiao Cai, Yaoyu Gu, Ke Li, Yu Zhao

**Affiliations:** 1 The National & Local Joint Engineering Laboratory of Animal Peptide Drug Development, College of Life Sciences, Hunan Normal University, Changsha, China; 2 Peptide and Small Molecule Drug R&D Platform, Furong Laboratory, Hunan Normal University, Changsha, China; 3 Department of Dermatology, Hunan Provincial People’s Hospital (The First Affiliated Hospital of Hunan Normal University), Changsha, China

**Keywords:** Melanoma, Ubiquitylation

## Abstract

Melanoma is a highly aggressive malignancy in which dysregulated ubiquitin-dependent signaling contributes to tumor progression. *OTUD4* is frequently downregulated in melanoma, but the functional significance of this alteration remains unclear. Here, we identify OTUD4 as a dual-mode deubiquitinase that suppresses melanoma progression by coordinating K48- and K63-linked polyubiquitination. OTUD4 directly binds and stabilizes CYLD by counteracting K48-linked polyubiquitination, whereas it independently binds BCL3 and counteracts BCL3 K63-linked polyubiquitination. OTUD4 depletion promotes BCL3 ubiquitination and nuclear translocation in melanoma cells, leading to increased melanoma cell proliferation and tumor growth in clonogenic and xenograft assays. Clinically, expression of *OTUD4* and *CYLD* progressively decreases from normal skin through benign nevi to melanoma, with a further reduction in highly metastatic tumors. Reduced expression of *OTUD4* and *CYLD* is associated with poor prognosis in patients with melanoma. Together, these results reveal an OTUD4–CYLD–BCL3 regulatory axis in melanoma, in which OTUD4 stabilizes CYLD and inhibits BCL3 activity.

**Figure:**
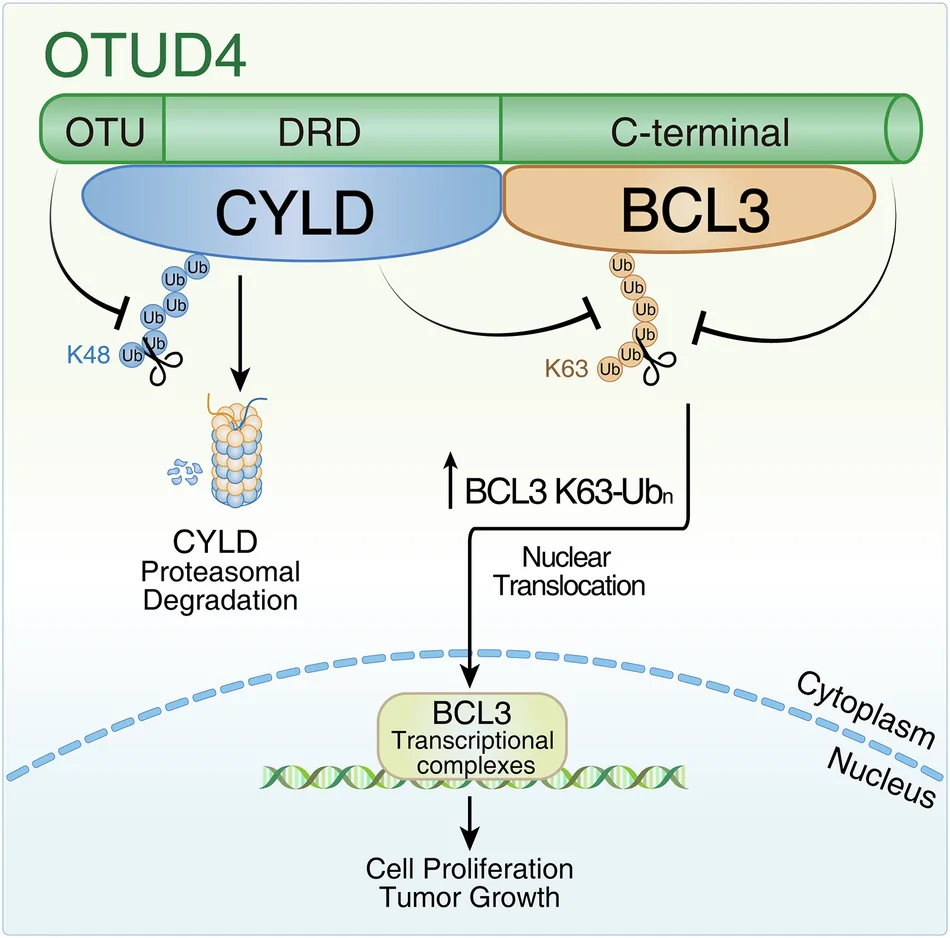

## Introduction

Melanoma is a highly aggressive malignancy with a propensity for early metastasis and frequent therapeutic resistance to targeted agents and immune checkpoint blockade [1–3]. Although significant therapeutic progress has been made in recent years, including the development of BRAF/MEK inhibitors, long-term clinical benefit remains limited in many patients [4, 5]. Constitutive activation of NF-κB signaling is a hallmark of melanoma and promotes tumor cell proliferation, survival, invasion, and tumor–immune signaling [6, 7]. The atypical IκB family member BCL3 functions as a nuclear co-regulator of noncanonical NF-κB–dependent transcription in melanoma, inducing the expression of proliferative genes such as *Cyclin D1* [8–10]. In addition, melanoma progression is driven by post-translational signaling that enables rapid and reversible reconfiguration of oncogenic programs [11–15].

Ubiquitination is a key post-translational mechanism that regulates the stability, subcellular localization, and activity of core signaling regulators across multiple cancer types [16–21]. This regulation is dynamically balanced by the opposing activities of E3 ubiquitin ligases and deubiquitinases (DUBs), which modulate the strength and duration of oncogenic pathway activation [22–26]. Disruption of this regulatory balance has been linked to aberrant NF-κB signaling and malignant progression across multiple cancer types, including melanoma [27, 28]. BCL3 activity is tightly controlled by ubiquitin-dependent mechanisms, with K63-linked polyubiquitination favoring its nuclear activity and promoting NF-κB–dependent transcription [9, 29]. BCL3 also undergoes ubiquitination-mediated proteasomal degradation [29–31], although the specific type of ubiquitin chain remains unclear. In melanoma, the deubiquitinase CYLD is frequently downregulated and functions as a negative regulator of NF-κB and BCL3, consistent with a tumor-suppressive role [9, 32–36]. CYLD is transcriptionally suppressed by factors such as Snail [10], and its protein levels are regulated by ubiquitin-dependent protein stability control, with several E3 ligases promoting CYLD ubiquitination and degradation [37–41]. Whether CYLD itself is subject to upstream DUB-mediated regulation that stabilizes its protein levels in melanoma remains unknown.

OTUD4, a member of the ovarian tumor (OTU) family of deubiquitinases, is dysregulated in multiple solid tumors and has been implicated in tumor progression [42–49]. Two distinct modes of OTUD4 function have been described. In one mode, OTUD4 regulates K48-linked polyubiquitination through a noncanonical, activity-independent mechanism, acting as a scaffold via its deubiquitinase recruitment domain (DRD) to engage partner DUBs, including USP7 and USP9X, thereby stabilizing selected substrates [50–52]. In parallel, OTUD4 possesses intrinsic catalytic activity toward K63-linked polyubiquitin chains. This activity is regulated by phosphorylation and modulates K63-dependent signaling across diverse cellular contexts, including canonical NF-κB signaling by regulating substrates such as MYD88 and TAK1 [45, 53, 54]. Notably, CYLD-focused proteomic datasets show greater total and unique peptide coverage for OTUD4 than for several other DUBs, including USP10 and USP15 [55]. This enrichment suggests a previously underrecognized association between OTUD4 and CYLD that may be functionally relevant. Whether these distinct ubiquitin-regulatory modes of OTUD4 are functionally coordinated to control the CYLD–BCL3 axis, however, remains unclear.

In this study, we identify a previously unrecognized OTUD4–CYLD–BCL3 regulatory axis in melanoma. OTUD4 coordinates distinct K48- and K63-linked deubiquitinase activities to regulate CYLD stability and BCL3-dependent, noncanonical NF-κB signaling. Disruption of this axis is linked to melanoma growth and progression. Clinically, OTUD4 and CYLD expression progressively decrease during melanoma development. Together, these results reveal a novel mechanism by which a single deubiquitinase integrates chain-specific deubiquitination across multiple substrates to modulate transcriptional programs implicated in melanoma progression.

## Materials and methods

### Cell culture

HEK293T cells were obtained from the American Type Culture Collection (ATCC, USA). Human A375 melanoma cells (IM-H332) were purchased from Xiamen Immocell Biotechnology Co., Ltd. (China). Human H1299 cells (SCSP-589) and A549 cells (SCSP-503) were obtained from the Cell Bank of the Chinese Academy of Sciences (China). All cells were cultured in Dulbecco’s modified Eagle medium (DMEM, 10313039, Gibco, USA) supplemented with 10% fetal bovine serum (FBS, 10099-141 C, Gibco, USA) and 1% penicillin–streptomycin (15140122, Gibco, USA), and maintained at 37 °C in a humidified atmosphere containing 5% CO₂. Routine mycoplasma testing was performed using the Myco-Lumi Luminescent Mycoplasma Detection Kit (C0297, Beyotime, China) to confirm the absence of contamination.

### Antibodies and reagents

Primary antibodies used in this study included anti-Flag antibodies (F3165 and F7425, Sigma-Aldrich, USA), anti-ubiquitin antibody (sc-8017, Santa Cruz Biotechnology, USA), anti-His antibody (ab18184, Abcam, UK), anti-MBP antibody (E8030, New England Biolabs, USA), anti-Cyclin D1 antibody (R380999, Zenbio, China), anti-p50 antibody (F0439, Selleck Chemicals, USA), anti-USP9X antibody (A301-351A, Bethyl, USA), anti-ALKBH3 antibody (09-882, MilliporeSigma, USA), anti-GAPDH antibody (M20006, Abmart, China), HRP-conjugated anti-β-actin antibody (A00730, GenScript, USA), and HRP-conjugated anti-β-tubulin antibody (E021043, EarthOx, USA). Primary antibodies against OTUD4 (25070-1-AP), CYLD (11110-1-AP), BCL3 (23959-1-AP), USP7 (26948-1-AP), and HA (51064-2-AP) were purchased from Proteintech (China). Secondary antibodies included HRP-conjugated goat anti-mouse IgG (H + L) (SA00001-1), HRP-conjugated goat anti-rabbit IgG (H + L) (SA00001-2), CoraLite488-conjugated goat anti-rabbit IgG (H + L) (SA00013-2), and CoraLite594-conjugated goat anti-mouse IgG (H + L) (SA00013-3) (Proteintech, China). Cycloheximide (CHX, 2112, Cell Signaling Technology, USA) and MG132 (S2619, Selleck Chemicals, USA) were used as indicated.

### Plasmids

Full-length human OTUD4 cDNA was used as the template to generate mutant constructs, as previously described [54]. HA-CYLD was obtained from Addgene (15506, USA). The pLV3-CMV-BCL3-3×FLAG-CopGFP-Puro plasmid was purchased from Miaoling (P56496, China). OTUD4 and CYLD truncation mutants, as well as point mutants, were generated by PCR-based site-directed mutagenesis. All constructs were cloned into pENTR-3C or pENTR-4 vectors (Invitrogen, USA) using standard PCR protocols and subsequently transferred into pHAGE-Flag or pHAGE-HA destination vectors by Gateway LR recombination. The coding sequences of shRNA-resistant OTUD4 wild-type (WT) and the catalytically inactive C45A were codon-optimized to evade shRNA targeting, with seven synonymous substitutions introduced into the ORF region corresponding to the shOTUD4-targeted sequence. The shBCL3 or shCYLD-targeted sequence was located in the 3′ UTR to avoid shRNA-mediated knockdown of exogenous BCL3 or CYLD.

### Immunoprecipitation and western blotting

For immunoprecipitation (IP), cells were lysed in Triton lysis buffer containing 1% Triton X-100, 150 mM NaCl, 50 mM Tris-HCl (pH 7.9), and 10% glycerol, supplemented with protease and phosphatase inhibitor cocktails (P1051, Beyotime, China) and 2 mM DTT. Lysates were rotated at 4°C for 15 min, cleared by centrifugation at 15,000 × *g* for 15 min at 4 °C, and the supernatants were used for IP. For Flag IP or HA IP, cleared lysates were incubated overnight at 4 °C with anti-Flag M2-agarose beads (A2220, Sigma-Aldrich, USA) or anti-HA magnetic beads (B26201, Selleck, USA), respectively.

For endogenous IP, lysates were incubated with anti-OTUD4 or anti-CYLD antibodies, or rabbit IgG control, overnight at 4 °C. Immune complexes were captured with Protein A/G magnetic beads (B23202, Selleck, USA) for 4 h.

For denaturing IP, lysates were denatured in SDS-containing buffer, diluted to 0.1% SDS in lysis buffer, and subjected to Flag IP using anti-Flag M2-agarose beads. Beads were washed with lysis buffer, and bound proteins were eluted in Laemmli sample buffer.

For western blotting (WB), proteins were separated by SDS–PAGE, transferred to PVDF membranes, blocked with 5% non-fat milk in TBST, incubated with primary antibodies overnight at 4 °C and with HRP-conjugated secondary antibodies at room temperature, and signals were detected using enhanced chemiluminescence.

### shRNA knockdown and CRISPR/Cas9 knockout

For shRNA-mediated knockdown, target-specific sequences were cloned into the pLKO.1-TRC vector (10878, Addgene, USA). The shRNA constructs used in this study included shOTUD4 #1 (5′-GCT GAT TAT GAA GAG TCA CTA-3′), shOTUD4 #2 (5′-GAT GAA GGT TAT CAG TAC CAT-3′), shCYLD (5′-CCT GGA GGA TTA GGG TAT TTA-3′), shBCL3 #1 (5′-TGC CAC CCA CAT CTT CCA TTT-3′), shBCL3 #2 (5′-CTC GAC ATC TAC AAC AAC CTA-3′), shUSP9X #1 (5′-GAG AGT TTA TTC ACT GTC TTA-3′), shUSP9X #2 (5′-CGA TTC TTC AAA GCT GTG AAT-3′), and shUSP9X #3 (5′-CGC CTG ATT CTT CCA ATG AAA-3′). Lentiviral particles were packaged in HEK293T cells and used to transduce target cells. Transduced cells were selected with puromycin for 3 days to establish stable knockdown cell lines. Knockdown efficiency was verified by WB. A non-targeting shRNA was used as a control.

For CRISPR/Cas9-mediated knockout of OTUD4, single-guide RNAs (sgRNAs) were designed and cloned into the lentiCRISPR v2 vector (52961, Addgene, USA). The sgRNA sequences targeting human OTUD4 were sgOTUD4 #1 (5′-AGG CCA TTC TGA CTT CAA CA-3′) and sgOTUD4 #2 (5′-TCG CCA TGT TGA AGT CAG AA-3′). Lentiviral particles were generated in HEK293T cells and used to transduce target cells, followed by puromycin selection. Single-cell clones were isolated by limiting dilution, expanded, and validated for OTUD4 knockout by WB. All constructs generated in this study were confirmed by DNA sequencing.

### RNA isolation and RT-qPCR

Total RNA was isolated using the RNeasy RNA Isolation Kit (R0026, Beyotime, China). Five hundred nanograms of total RNA were reverse-transcribed into cDNA using the PrimeScript RT Reagent Kit (RR047A, Takara, Japan) according to the manufacturer’s instructions. Reverse transcription quantitative PCR (RT-qPCR) was performed using SYBR Green qPCR Mix (TSE501, Tsingke, China). The primer sequences used for transcript quantification were as follows: *CYLD* forward, 5′-TTC ACT GAC GGG GTG TAC CA-3′; *CYLD* reverse, 5′-CAG GAC CTG CGT AAT CAC TTT C-3′; *β-actin* forward, 5′-TCA TCA CTA TTG GCA ACG AGC GGT TC-3′; *β-actin* reverse, 5′-TAC CAC CAG ACA GCA CTG TGT TGG CA-3′. Relative mRNA levels were normalized to *β-actin*.

### Immunofluorescence

For immunofluorescence (IF), A375 cells expressing the indicated constructs were seeded on sterile round glass coverslips and fixed with 4% paraformaldehyde (P0099, Beyotime, China) for 20 min at 37 °C. Cells were washed three times with TBST and blocked with blocking buffer. Primary antibodies diluted in TBST were incubated with the samples overnight at 4 °C. After four TBST washes, samples were incubated with fluorophore-conjugated secondary antibodies and DAPI for 2 h at 37 °C in the dark. Coverslips were washed five times with TBST, mounted with an antifade mounting medium, and imaged using a fluorescence microscope (Olympus, Japan). Colocalization between OTUD4 and CYLD was quantified using the Pearson correlation coefficient (PCC).

### Generation of lentivirus and stable cell lines

Lentiviral particles were generated in HEK293T cells by co-transfecting pLKO.1, pHAGE, or lentiCRISPR v2 vectors with the packaging plasmids psPAX2 and pMD2.G using polyethyleneimine (PEI, HY-K2014, MCE, USA). Viral supernatants were collected 48–72 h post-transfection, clarified by centrifugation, and passed through 0.45-μm filters. Target cells were transduced with filtered viral supernatant containing polybrene (8 μg/mL) for 12 h. Stable cell lines were selected with puromycin (1 μg/mL) or blasticidin (5 μg/mL) for 3 days and subsequently maintained under selection-free conditions unless otherwise indicated.

For the generation of OTUD4-knockout A375 cells, single-guide RNAs targeting OTUD4 were cloned into the lentiCRISPR v2 vector by annealing complementary oligonucleotides, and knockout cell populations were obtained following lentiviral transduction and puromycin selection.

### Halo-TUBE and Halo-NZF2 pull-downs

Halo-Tag resins (G1915, Promega, USA) were conjugated with recombinant Halo-TUBE (K48-specific) [56] or Halo-NZF2 (K63-specific) [54] proteins, which selectively bind K48-linked or K63-linked polyubiquitin chains and ubiquitinated proteins, respectively. Proteins were extracted from A375 cells using an SDS-denaturing buffer supplemented with a protease and phosphatase inhibitor cocktail and 2 mM DTT, boiled for 10 min, and passed through 0.45-μm filters. The extracts were diluted ten-fold with 1% Triton X-100 lysis buffer and incubated with Halo-resin–bound recombinant proteins (Halo-TUBE or Halo-NZF2) for each sample with rotation at 4 °C overnight. The Halo resins were washed five times, eluted with Laemmli buffer, resolved by SDS–PAGE, and analyzed by WB using the indicated antibodies.

### MBP and His pull-down assays

For maltose-binding protein (MBP) pull-down assays, plasmids encoding MBP alone, MBP-OTUD4 WT, MBP-OTUD4 C45A, His-Flag-CYLD WT, His-Flag-CYLD C598S, and Flag-BCL3 were expressed in *E. coli*. MBP-tagged proteins were purified using amylose resin (E8021, NEB, USA). Purified MBP fusion proteins or MBP alone were immobilized on amylose resin and washed with binding buffer. The resin was then incubated with the indicated Flag-tagged prey proteins at 4 °C with gentle rotation for 12 h. After extensive washing, bound proteins were eluted in Laemmli sample buffer, resolved by SDS–PAGE, and analyzed by WB using the indicated antibodies.

For His pull-down assays, plasmids encoding MBP-OTUD4, His-Flag alone, His-Flag-CYLD WT, or His-Flag-CYLD C598S were expressed in *E. coli*. His-tagged proteins were purified using High Affinity Ni-Charged Resin FF (L00666, GenScript, USA). Purified His-tagged control or fusion proteins were immobilized on Ni-charged resin and washed with binding buffer. The resin was then incubated with the indicated MBP-tagged OTUD4 protein at 4 °C overnight. After extensive washing with buffer containing 20 mM imidazole, bound proteins were eluted in Laemmli sample buffer, resolved by SDS–PAGE, and analyzed by WB using the indicated antibodies.

### Cell proliferation and colony formation assays

For cell proliferation assays, cells were seeded into 96-well plates in complete DMEM. Cell viability of A375 cells was quantified using the Cell Counting Kit-8 (CCK-8) assay (K1018, ApexBio, USA) starting on day 2. Briefly, 10 μL of CCK-8 solution was added to each well and incubated at 37 °C for 1 h, followed by absorbance measurement at 450 nm.

For colony formation assays, cells (1 × 10^3^ cells per well) were seeded into 6-well plates and cultured under standard conditions. Colonies were fixed with 4% paraformaldehyde (P0099, Beyotime, China) for 20 min and stained with 1% crystal violet (C0121, Beyotime, China) at 25 °C for 20 min.

### Mouse xenograft experiments

Male BALB/c nude mice aged 4–5 weeks (Shanghai SLAC Laboratory Animal, China) were randomly assigned to two groups, with group allocation performed in a blinded manner, and used for tumor xenograft experiments. A375 cells stably expressing control or OTUD4 sgRNA (2 × 10^6^ cells) were suspended in 200 μL PBS and subcutaneously injected into the left flank of each mouse. Mice were housed in a specific pathogen-free facility under controlled conditions (24 °C, 45–60% humidity, and a 12 h light/dark cycle). Tumor growth was monitored every 3 days using a digital caliper, and tumor volume was calculated using the formula: volume = length × width^2^/2. At the experimental endpoint, mice were euthanized, and tumors were excised for further analysis. All animal experiments were performed in accordance with relevant ethical guidelines and regulations and were approved by the Biomedical Research Ethics Committee of Hunan Normal University.

### Bioinformatic analysis

Microarray gene expression datasets GSE7929 [57], GSE3189 [58], and GSE19234 [59] were retrieved from the Gene Expression Omnibus (GEO) database. Gene expression profiling and Spearman’s rank correlation analysis in the TCGA cohort were performed using the GEPIA web tool. Comparative gene expression analyses across cancer types were conducted using the Kaplan–Meier Plotter platform (TNMplot) [60]. Analyses of gene expression in sun-exposed and non–sun-exposed skin using GTEx data accessed through the Human Protein Atlas database, as well as immunohistochemical assessment of protein expression in skin tissues, were performed using the Human Protein Atlas database. Protein structure prediction and interaction modeling were performed using AlphaFold 3 [61], and PyMOL was used for structural visualization and for generating protein interaction representations.

### Statistical analysis

All statistical analyses were performed using GraphPad Prism 9 (GraphPad Software, USA). Numerical data were presented as mean ± standard deviation (SD). Spearman’s correlation analysis, Mann–Whitney *U* test, Gehan–Breslow–Wilcoxon test, log-rank test, unpaired two-tailed Student’s *t*-test, and one-way ANOVA followed by Tukey’s multiple comparisons test were used to assess statistical significance, as specified in the figure legends. Blinding was performed during group allocation for mouse xenograft experiments but not for other experiments. Objective measurements were used to reduce potential bias. No samples or experimental data were excluded from experiments or outcome assessment. A *p* < 0.05 was considered statistically significant. Statistical significance was denoted as follows: *p < 0.05; **p < 0.01; ***p < 0.001; ns, not significant.

## Results

### OTUD4 downregulation is associated with melanocytic progression, melanoma aggressiveness, and poor prognosis

**Fig. 1:**
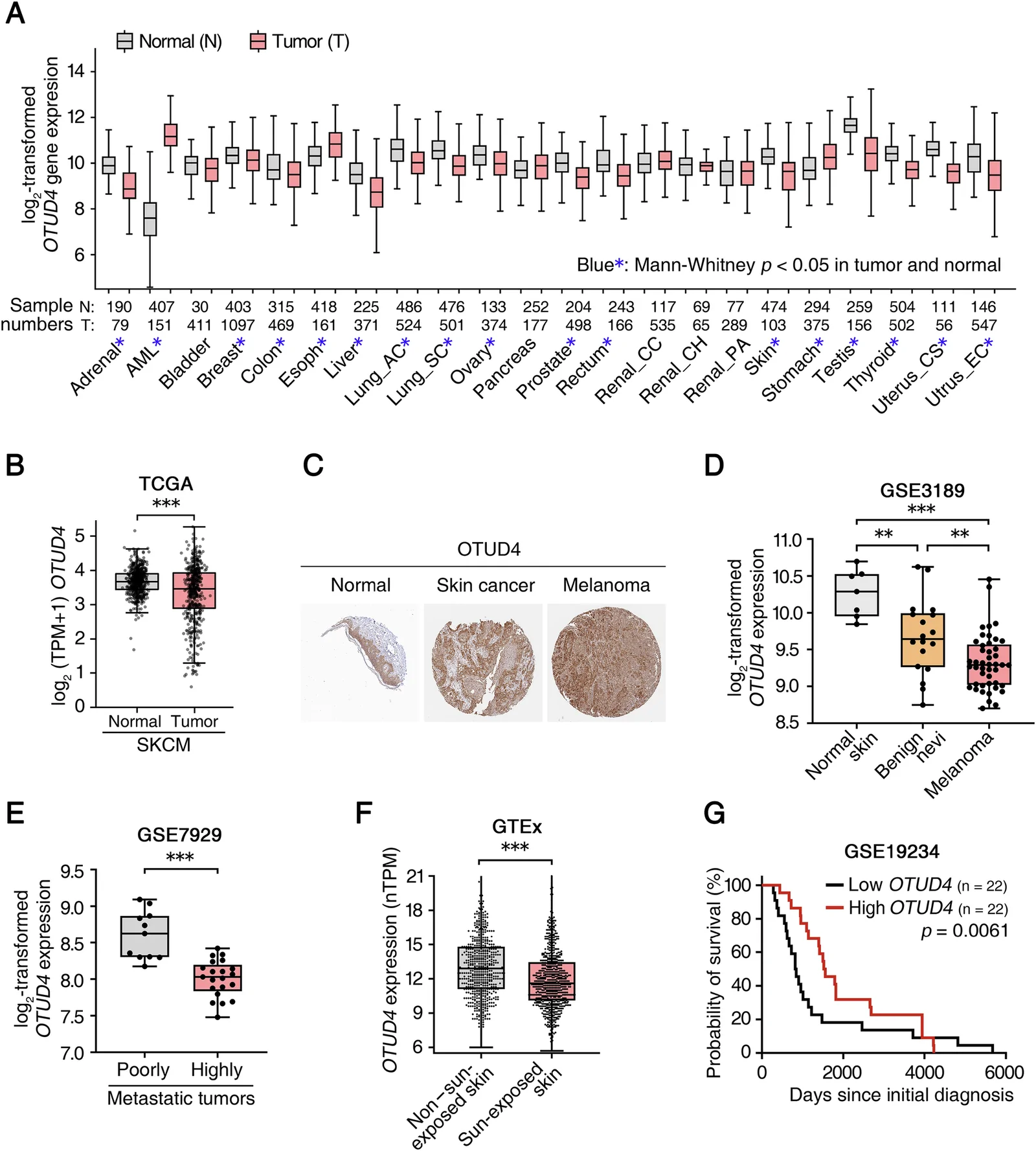
OTUD4 downregulation is associated with melanocytic progression, melanoma aggressiveness, and poor prognosis. **A** Pan-cancer *OTUD4* mRNA levels in normal and tumor tissues from TNMplot based on RNA-seq data. Blue asterisks mark tumor–normal differences (Mann–Whitney *U* test). **B** *OTUD4* mRNA levels in normal skin (*n* = 558) and skin cutaneous melanoma (*n* = 469) from GEPIA. **C** Representative immunohistochemistry images showing OTUD4 protein levels in normal skin, non-melanoma skin cancer, and melanoma tissues from the Human Protein Atlas. **D** *OTUD4* mRNA levels in normal skin (*n* = 7), benign nevi (*n* = 18), and malignant melanoma (*n* = 45) from the GEO dataset GSE3189 (one-way ANOVA followed by Tukey’s multiple comparisons test). **E** *OTUD4* mRNA levels in poorly metastatic and highly metastatic melanoma cell lines from the GEO dataset GSE7929 (two-tailed unpaired Student’s *t* test). **F** *OTUD4* mRNA levels in sun-exposed and non–sun-exposed skin from GTEx data accessed through the Human Protein Atlas database (two-tailed unpaired Student’s *t* test). **G** Kaplan–Meier analysis of disease progression stratified by *OTUD4* expression in the GEO dataset GSE19234 (Gehan–Breslow–Wilcoxon test). Statistical tests are indicated in each panel. Error bars indicate means ± SD. *p < 0.05, **p < 0.01, ***p < 0.001.

To assess OTUD4 expression patterns across cancers, we analyzed pan-cancer transcriptomic datasets, which showed heterogeneous expression among tumor types and frequent downregulation, including in skin cancers (Fig. 1A). Analysis of RNA-seq datasets from the TCGA skin cutaneous melanoma (SKCM) cohort revealed that *OTUD4* mRNA levels were significantly lower in melanoma tissues compared to normal skin (Fig. 1B). Immunohistochemistry from the Human Protein Atlas further revealed reduced OTUD4 protein levels in melanoma relative to adjacent normal skin (Fig. 1C).

To evaluate *OTUD4* expression during melanocytic progression, we analyzed the GSE3189 cohort, which showed a progressive decrease in *OTUD4* expression from normal skin to benign nevi to melanoma (Fig. 1D). *OTUD4* expression was lower in highly metastatic melanoma cells compared to poorly metastatic counterparts (Fig. 1E), and significantly reduced in sun-exposed skin relative to non–sun-exposed skin (Fig. 1F). Reduced *OTUD4* expression was also associated with significantly worse survival in melanoma patients (Fig. 1G). Collectively, these data indicate that progressive downregulation of OTUD4 is associated with melanocytic progression, melanoma aggressiveness, and poor prognosis.

### OTUD4 directly binds CYLD

CYLD is a well-characterized tumor suppressor in melanoma, frequently downregulated in melanoma [10, 62, 63]. Transcriptomic analyses revealed that *OTUD4* and *CYLD* mRNA levels are positively correlated in both normal skin and melanoma samples (Supplementary Fig. 1A, B). *CYLD* expression progressively decreased from normal skin to benign nevi and melanoma (Supplementary Fig. 1C, D), was further reduced in highly metastatic melanoma (Supplementary Fig. 1E), and was lower in sun-exposed skin compared to non-sun-exposed skin (Supplementary Fig. 1F). Reduced CYLD expression was also associated with worse survival in melanoma patients (Supplementary Fig. 1G). These clinical and experimental observations indicate that OTUD4 and CYLD follow parallel expression patterns during melanoma progression.

**Fig. 2:**
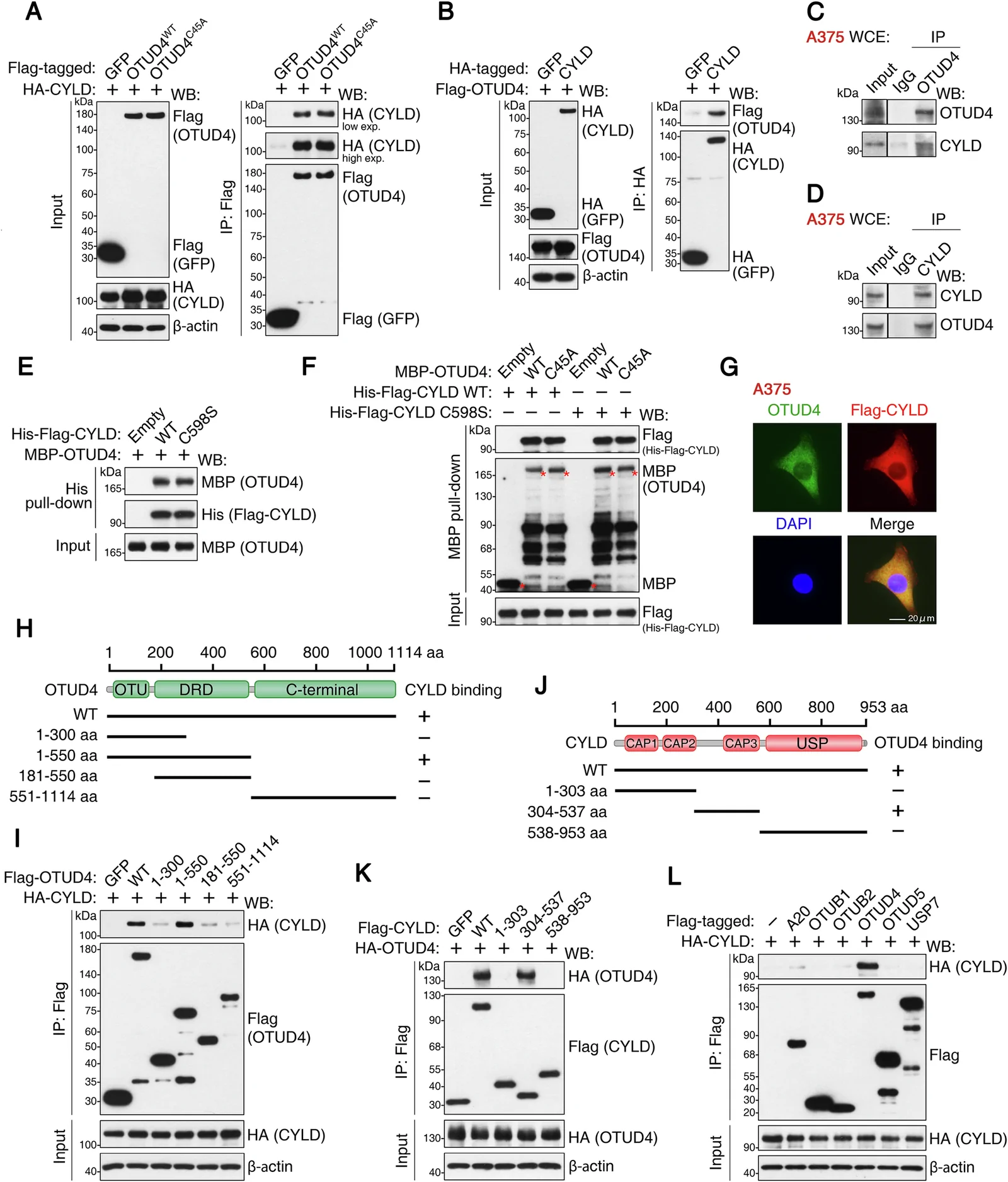
OTUD4 directly binds CYLD. **A** HEK293T cells were transfected with HA-CYLD and Flag-OTUD4 WT or C45A mutant. Flag immunoprecipitation (IP) was performed and analyzed by western blotting (WB). **B** Reciprocal HA IP was performed and analyzed by WB. Endogenous OTUD4–CYLD interaction in A375 cells was analyzed by IP with control IgG, anti-OTUD4 antibody (**C**), or anti-CYLD antibody (**D**), followed by WB. MBP-OTUD4 (WT or C45A) and His-Flag-CYLD (WT or C598S), expressed in *E. coli*, were subjected to His pull-down (**E**) or MBP pull-down (**F**), followed by WB. Red asterisks in (**F**) indicate bands corresponding to MBP alone and full-length MBP-OTUD4. **G** Colocalization of OTUD4 (green) and Flag-CYLD (red) in A375 cells was analyzed by immunofluorescence microscopy. Scale bar, 20 μm. Pearson correlation coefficient (PCC = 0.750 ± 0.004, *n* = 50 cells). **H** Schematic of full-length OTUD4 and its deletion constructs. **I** HEK293T cells transfected with HA-CYLD and Flag-OTUD4 constructs were analyzed by Flag IP and WB. **J** Schematic of full-length CYLD and its deletion constructs. **K** HEK293T cells transfected with HA-OTUD4 and Flag-CYLD constructs were analyzed by Flag IP and WB. **L** HEK293T cells transfected with HA-CYLD and Flag-tagged deubiquitinases (A20, OTUB1, OTUB2, OTUD4, OTUD5, USP7) were subjected to Flag IP and WB. Experiments in **A**–**G**, **I**, **K**, **L** were independently repeated at least twice with similar results.

Re-analysis of published CYLD proteomic datasets [55] revealed that OTUD4 had markedly higher total and unique peptide coverage than several other deubiquitinases, including USP10 and USP15 (Supplementary Fig. 1H). This proteomic enrichment and concordant expression patterns prompted us to investigate whether OTUD4 and CYLD physically associate in melanoma models. Co-immunoprecipitation (co-IP) assays showed that HA-tagged CYLD (HA-CYLD) associated with Flag-tagged OTUD4 (Flag-OTUD4) in HEK293T cells, including the catalytically inactive C45A mutant (Fig. 2A), and this interaction was confirmed by reciprocal immunoprecipitation using HA-CYLD (Fig. 2B). Endogenous OTUD4 co-immunoprecipitated with CYLD in A375 melanoma cells, and reciprocal endogenous co-IP further confirmed the OTUD4–CYLD interaction (Fig. 2C, D). Recombinant protein pull-down assays using purified MBP-OTUD4 and His-Flag-CYLD confirmed direct binding between OTUD4 and CYLD and indicated that the catalytic activity of either protein is not required for this interaction (Fig. 2E, F). Immunofluorescence analysis further showed that OTUD4 and CYLD primarily co-localized in the cytoplasm (Fig. 2G).

To map the interaction interface, OTUD4 truncation mutants were generated and analyzed by co-IP in HEK293T cells. OTUD4 residues 1–550 are required for CYLD association, encompassing the deubiquitinase recruitment domain (DRD; residues 181–550) (Fig. 2H, I). Conversely, a CYLD fragment containing the CAP3 region (residues 304–537) was sufficient to support interaction with OTUD4, whereas the CYLD 1–303 and 538–953 fragments did not show detectable binding (Fig. 2J, K). To assess interaction specificity, CYLD binding was compared across multiple deubiquitinases, including representative members of the OTU family and USP7, a well-characterized DUB. Although weak interactions with A20 (TNFAIP3) were detectable upon extended exposure, OTUD4 showed the strongest association with CYLD (Fig. 2L). Collectively, these data indicate that OTUD4 directly binds CYLD through its N-terminal region, which substantially overlaps with the DRD.

### OTUD4 counteracts K48-linked polyubiquitination of CYLD

**Fig. 3:**
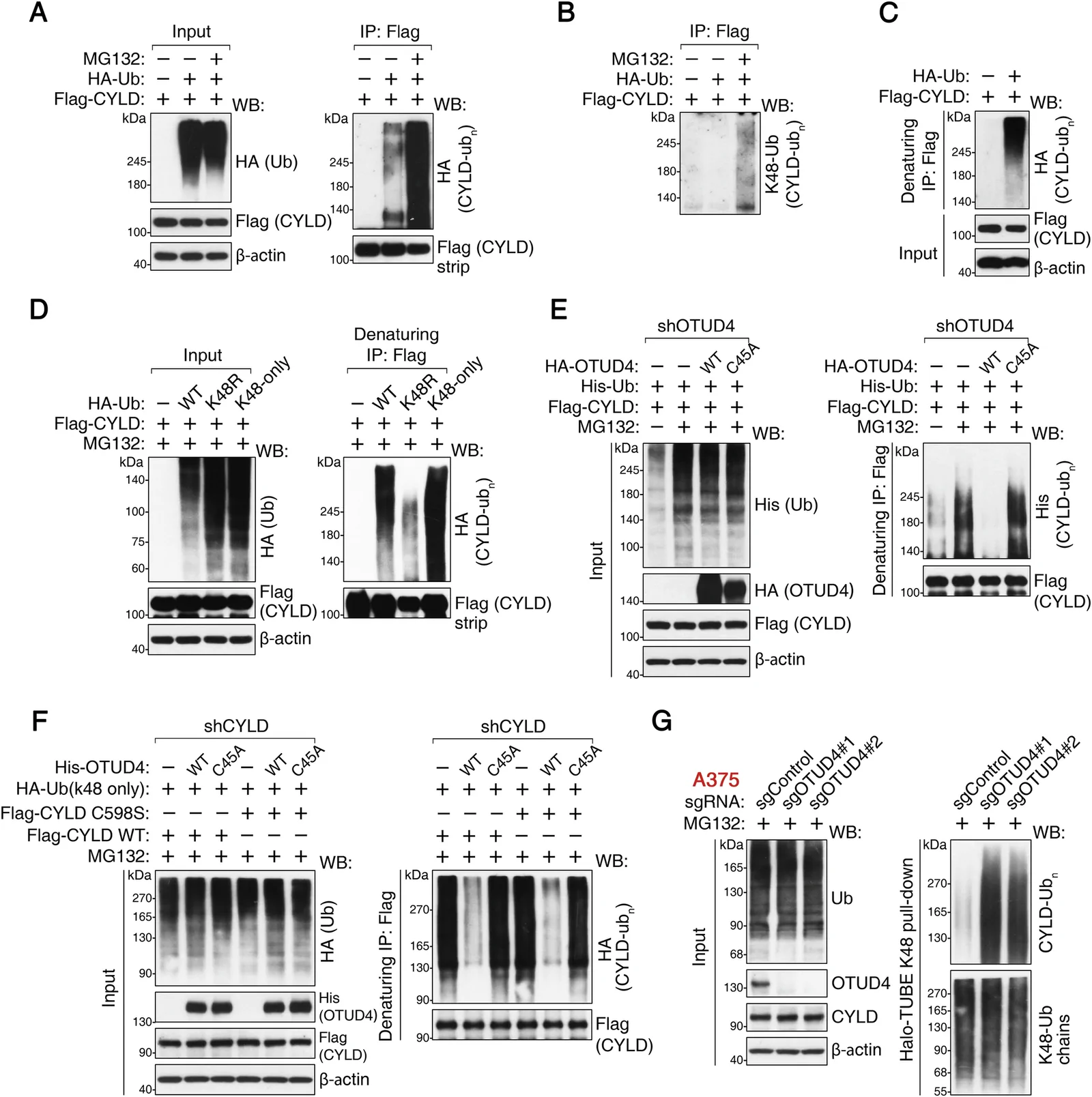
OTUD4 counteracts K48-linked polyubiquitination of CYLD. **A**, **B** HEK293T cells stably expressing Flag-CYLD were transfected with empty vector or HA-ubiquitin (HA-Ub), treated with MG132 as indicated, and subjected to Flag IP. Polyubiquitinated CYLD was detected by WB with anti-HA (**A**) or anti-K48-linked ubiquitin (**B**) antibodies. **C** HEK293T cells transfected with Flag-CYLD alone or together with HA-Ub were subjected to Flag IP under SDS-denaturing conditions. **D** CYLD is predominantly modified by K48-linked polyubiquitin chains. HEK293T cells expressing Flag-CYLD, together with HA-Ub(WT), HA-Ub(K48R), or HA-Ub(K48-only), were treated with MG132 and subjected to a denaturing Flag IP followed by WB. HEK293T cells expressing shRNA against OTUD4, Flag-CYLD, and His-Ub together with shRNA-resistant OTUD4 WT or C45A (**E**), or shRNA against CYLD together with shRNA-resistant Flag-CYLD WT or C598S, HA-Ub(K48-only), and His-OTUD4 WT or C45A (**F**), were subjected to denaturing IP followed by WB. Polyubiquitinated CYLD was detected using anti-His (**E**) or anti-HA (**F**) antibody. **G** Loss of OTUD4 increases endogenous CYLD K48-linked polyubiquitination. OTUD4-knockout A375 cells were subjected to Halo-TUBE enrichment specific for K48-linked polyubiquitin chains. Whole-cell lysates and bound fractions were analyzed by WB using the indicated antibodies. All experiments were independently repeated at least twice with similar results.

To determine whether CYLD is ubiquitinated in cells, Flag-CYLD was co-expressed with HA-ubiquitin in HEK293T cells. Flag IP analysis revealed ubiquitin-conjugated CYLD, which was further increased upon treatment with the proteasome inhibitor MG132 (Fig. 3A). A K48 linkage-specific antibody confirmed that CYLD carries K48-linked polyubiquitin chains (Fig. 3B), a modification associated with proteasomal degradation. Denaturing Flag IP verified that CYLD is covalently ubiquitinated (Fig. 3C), and analysis using ubiquitin mutants showed that K48-linked polyubiquitination represents the predominant ubiquitin species on CYLD (Fig. 3D).

To assess whether OTUD4 regulates CYLD ubiquitination, CYLD modification was examined in the presence of OTUD4. Wild-type (WT) OTUD4, but not the catalytically inactive C45A mutant, markedly reduced CYLD ubiquitination (Fig. 3E). This deubiquitination selectively targeted K48-linked polyubiquitin chains, as confirmed using K48-only ubiquitin mutants. In cells depleted of endogenous CYLD, OTUD4 also reduced K48-linked polyubiquitination of the catalytically inactive CYLD C598S mutant, indicating that CYLD catalytic activity is not required for OTUD4-mediated deubiquitination of CYLD itself (Fig. 3F). Conversely, CRISPR/Cas9-mediated OTUD4 knockout increased endogenous CYLD K48-linked polyubiquitination in A375 cells, as detected by enrichment using K48-specific Halo-TUBE beads (Fig. 3G). Collectively, these data indicate that OTUD4 counteracts K48-linked polyubiquitination of CYLD.

### OTUD4 stabilizes CYLD via a canonical, catalytic deubiquitination mechanism

**Fig. 4:**
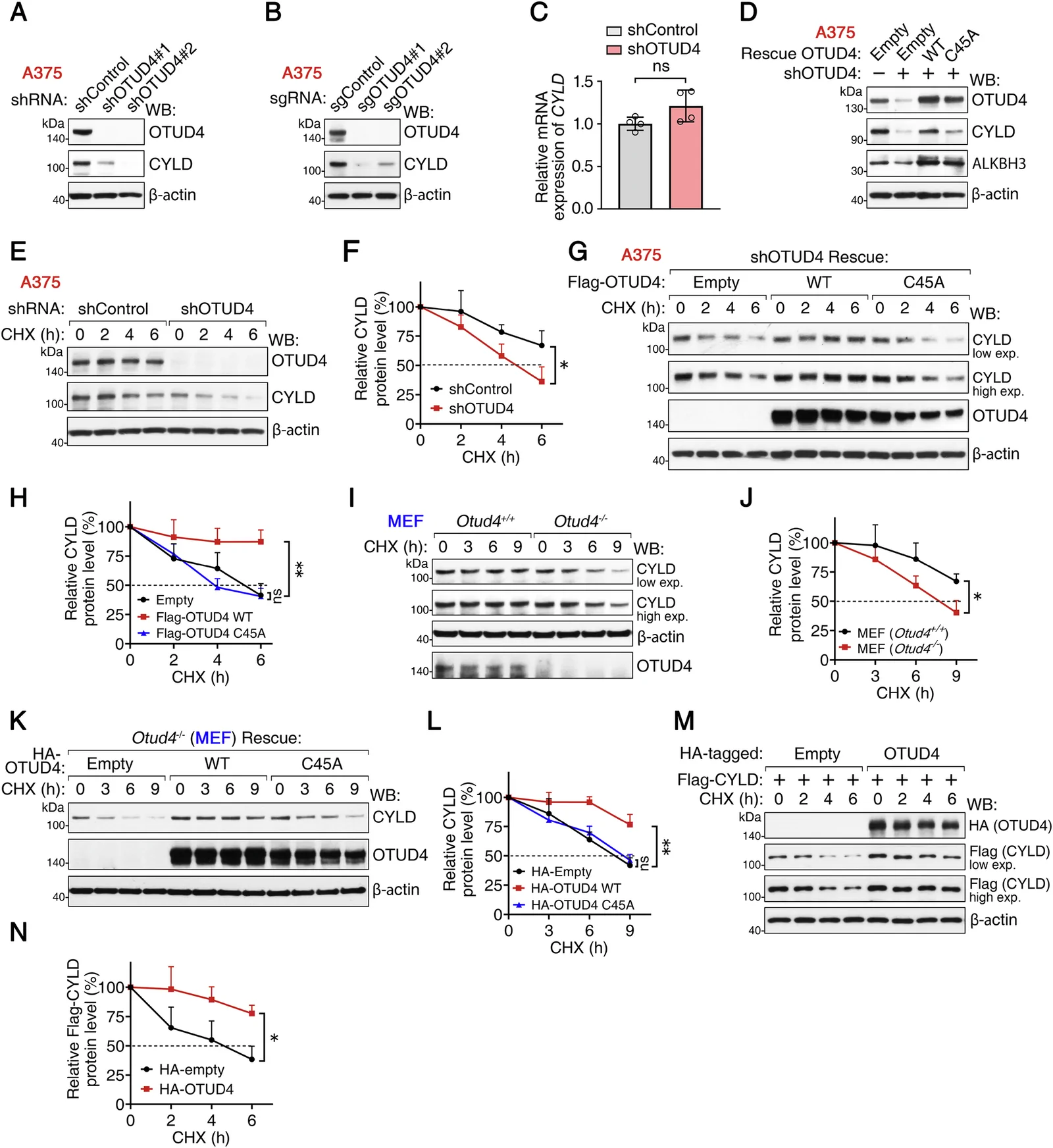
OTUD4 stabilizes CYLD via a canonical, catalytic deubiquitination mechanism. OTUD4 depletion in A375 cells by shRNA (**A**) or sgRNA (**B**) was confirmed by WB. **C** *CYLD* mRNA levels were not altered by OTUD4 depletion, as determined by RT-qPCR. **D** CYLD protein levels were restored by wild-type OTUD4 but not by the catalytic mutant C45A. A375 cells stably expressing Flag-OTUD4 WT or C45A, or empty vector, were transduced with OTUD4 shRNAs and analyzed by WB. A375 cells transduced with control or OTUD4 shRNA were treated with cycloheximide (CHX) and analyzed by WB (**E**), followed by CYLD quantification (**F**). OTUD4 WT, but not C45A, restores CYLD stability. OTUD4-knockdown A375 cells were transduced with Flag-OTUD4 WT or C45A, or control vector, treated with CHX, and analyzed by WB (**G**), followed by CYLD quantification (**H**). OTUD4 WT or OTUD4-knockout MEFs were treated with CHX and analyzed by WB (**I**), followed by quantification of CYLD (**J**). **K**, **L** Reintroduction of OTUD4 WT, but not C45A, rescues CYLD stability in OTUD4-knockout (*Otud4*^−/−^) MEFs. Cells were transduced with HA-OTUD4 WT or C45A, or a control vector, treated with CHX, and analyzed by WB (**K**), followed by quantification of CYLD (**L**). **M**, **N** OTUD4 overexpression stabilizes exogenous CYLD. HEK293T cells expressing Flag-CYLD were transfected with HA-OTUD4, treated with CHX, and analyzed by WB (**M**). Flag-CYLD levels were quantified by densitometry and normalized to β-actin (**N**). Data are mean ± SD from three (**F**, **H**, **J**, **L**, **N**) or four (C) independent experiments. Statistical significance was determined using a two-tailed unpaired Student’s *t* test. *p < 0.05, **p < 0.01. ns not significant. Experiments in **A**, **B**, **D**, **E**, **G**, **I**, **K**, **M** were independently repeated three times with similar results.

To examine the effect of OTUD4 on CYLD protein levels, OTUD4 was depleted in A375 cells using two independent shRNAs, and both shRNAs markedly reduced CYLD protein levels (Fig. 4A). This effect was independently confirmed by CRISPR/Cas9-mediated OTUD4 knockout, which similarly decreased CYLD levels (Fig. 4B). In contrast, CYLD mRNA levels remained unchanged following OTUD4 depletion (Fig. 4C), indicating post-transcriptional regulation. Furthermore, MG132 treatment restored CYLD protein levels in OTUD4-depleted and OTUD4-knockout cells, indicating proteasome-dependent loss of CYLD upon OTUD4 depletion (Supplementary Fig. 2A, B). Re-expression of OTUD4 WT, but not the catalytically inactive C45A mutant, restored CYLD protein levels (Fig. 4D). Notably, the previously reported OTUD4 substrate ALKBH3 [50] was rescued by both OTUD4 WT and C45A, consistent with its regulation via a noncanonical mechanism. To further distinguish CYLD regulation from noncanonical OTUD4 pathways, we assessed the involvement of USP9X and USP7. Knockdown of either USP9X or USP7 did not reduce CYLD protein levels in A375 cells (Supplementary Fig. 2C, D), whereas ALKBH3 levels were selectively decreased upon USP9X depletion. These results indicate that OTUD4 regulates CYLD through a canonical, catalytic mechanism.

Cycloheximide (CHX) chase assays further showed that OTUD4 depletion in A375 cells accelerated CYLD degradation and shortened its half-life (Fig. 4E, F), whereas re-expression of OTUD4 WT, but not C45A, restored CYLD stability (Fig. 4G, H). Consistently, OTUD4 knockout in mouse embryonic fibroblasts accelerated CYLD degradation, an effect that was reversed by OTUD4 WT but not the OTUD4 C45A mutant (Fig. 4I–L). Moreover, OTUD4 overexpression in HEK293T cells slowed the degradation of exogenous Flag-CYLD (Fig. 4M, N). Similar effects were observed in A549 and H1299 cells (Supplementary Fig. 2E, F), indicating that OTUD4 regulates CYLD stability across multiple cellular contexts. Collectively, these data indicate that OTUD4 stabilizes CYLD through a canonical, catalytic deubiquitination mechanism distinct from previously described noncanonical OTUD4 pathways.

### OTUD4 directly binds BCL3

**Fig. 5:**
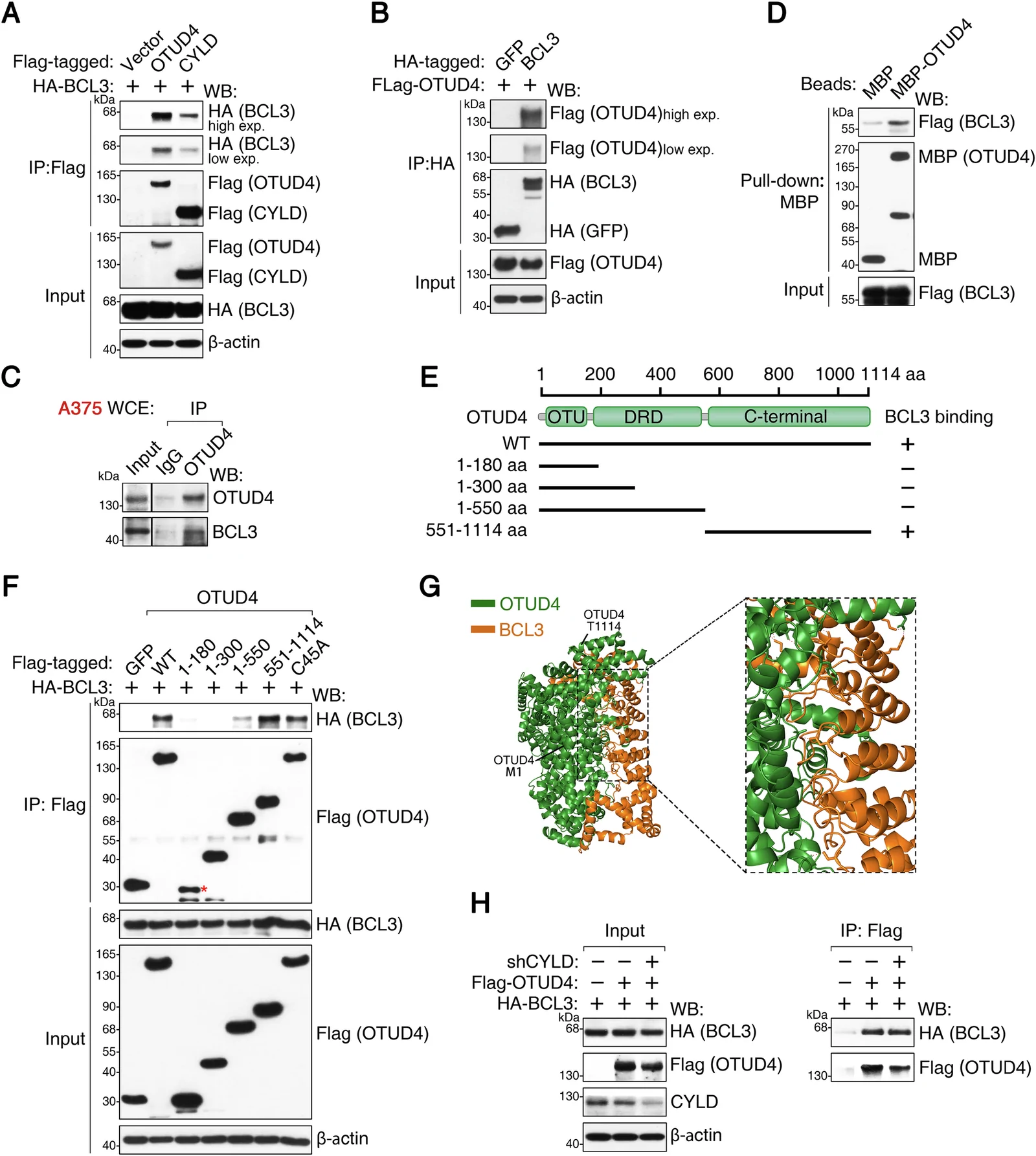
OTUD4 directly binds BCL3. **A** HEK293T cells were transfected with HA-BCL3 and Flag-OTUD4 or Flag-CYLD, followed by Flag IP and WB. **B** Reciprocal HA IP analysis of the OTUD4–BCL3 interaction. HEK293T cells were transfected with Flag-OTUD4 together with HA-GFP or HA-BCL3, followed by HA IP and WB. **C** Endogenous OTUD4–BCL3 interaction in A375 cells. Whole-cell lysates were subjected to IP using anti-OTUD4 antibody or control IgG, followed by WB. **D** MBP or MBP-OTUD4 and His-Flag-BCL3 were expressed in *E. coli* and subjected to MBP pull-down followed by WB. **E** Schematic of full-length OTUD4 and its deletion constructs. **F** HEK293T cells were transfected with HA-BCL3 and the indicated Flag-tagged OTUD4 constructs, followed by Flag IP and WB. **G** AlphaFold 3-predicted structural model of the OTUD4–BCL3 interaction. OTUD4 is shown in green, BCL3 in orange, and the predicted interface is shown at higher magnification on the right. **H** HEK293T cells transfected with HA-BCL3 and Flag-OTUD4 were further transduced with control or CYLD shRNAs, followed by Flag IP and WB. Experiments in **A**–**D**, **F**, **H** were independently repeated twice with similar results.

BCL3 is a well-established substrate of CYLD [9]. Given that OTUD4 binds CYLD and regulates CYLD stability, we next examined whether OTUD4 also interacts with BCL3. Co-IP assays revealed an interaction between OTUD4 and BCL3, which appeared more robust than the interaction with CYLD under the same experimental conditions (Fig. 5A). Reciprocal co-IP further confirmed this association (Fig. 5B). Endogenous OTUD4 was detected in complex with endogenous BCL3 in A375 cells, indicating that this interaction occurs under physiological conditions (Fig. 5C). To determine whether the interaction is direct, MBP pull-down assays using recombinant MBP-OTUD4 and Flag-BCL3 purified from *E. coli* showed direct binding in vitro (Fig. 5D).

To map the interaction interface, deletion mapping analyses showed that OTUD4 constructs containing the C-terminal region (residues 551–C terminus) mediated BCL3 association, whereas N-terminal fragments failed to interact (Fig. 5E, F), consistent with AlphaFold 3 predictions (Fig. 5G). Because CYLD also binds BCL3, we tested whether CYLD contributes to OTUD4–BCL3 binding. CYLD depletion did not detectably reduce this interaction (Fig. 5H). Collectively, these data indicate that OTUD4 binds BCL3 through its C-terminal region, which is distinct from the N-terminal region required for CYLD binding.

### OTUD4 and CYLD cooperate to limit K63-linked polyubiquitination of BCL3

**Fig. 6:**
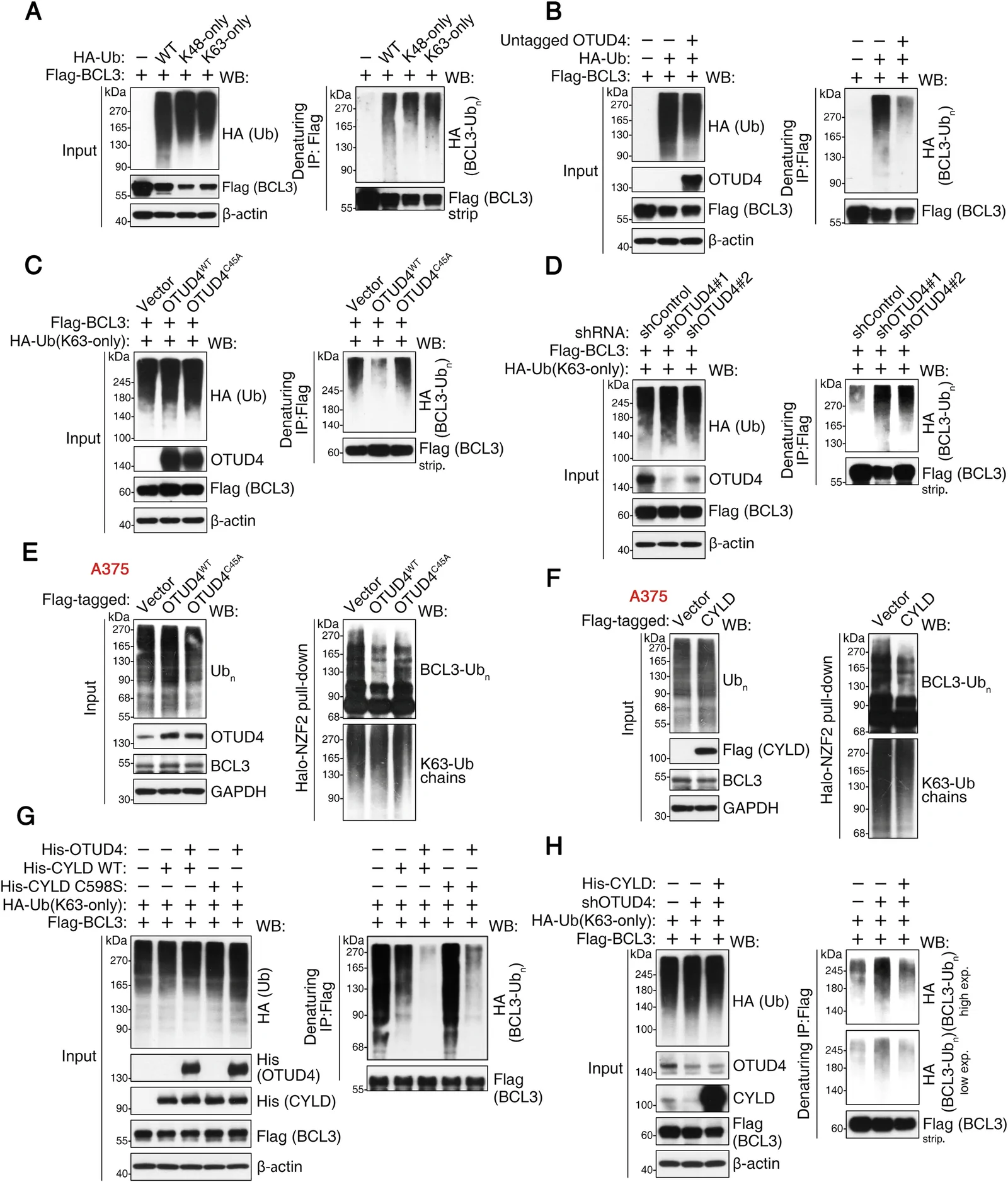
OTUD4 and CYLD cooperate to limit K63-linked polyubiquitination of BCL3. **A** HEK293T cells stably expressing Flag-BCL3 were transfected with HA-Ub(WT), HA-Ub(K48-only), or HA-Ub(K63-only), followed by denaturing Flag IP and WB. **B** HEK293T cells stably expressing Flag-BCL3 were transfected with HA-Ub(WT) together with empty vector or untagged OTUD4, followed by denaturing Flag IP and WB. **C** HEK293T cells stably expressing Flag-BCL3 were transfected with HA-Ub(K63-only) together with OTUD4 WT or the C45A mutant, followed by denaturing Flag IP and WB. **D** HEK293T cells stably expressing Flag-BCL3 and control or OTUD4 shRNAs were transfected with HA-Ub(K63-only), followed by denaturing Flag IP and WB. A375 cells expressing Flag-OTUD4 WT or C45A (**E**) or Flag-CYLD (**F**) were subjected to Halo-NZF2 enrichment specific for K63-linked polyubiquitin chains, and whole-cell lysates and bound fractions were analyzed by WB. **G** HEK293T cells stably expressing Flag-BCL3 were transfected with HA-Ub(K63-only) together with His-CYLD WT or C598S in the absence or presence of His-tagged OTUD4, as indicated, followed by denaturing Flag IP and WB. **H** HEK293T cells stably expressing Flag-BCL3 and OTUD4 shRNA were transfected with HA-Ub(K63-only) and His-CYLD, followed by denaturing Flag IP and WB. All experiments were independently repeated twice with similar results.

To determine whether OTUD4 regulates BCL3 ubiquitination, we first assessed the types of ubiquitin linkage on Flag-BCL3 using chain-restricted ubiquitin constructs. In HEK293T cells, BCL3 was modified by both K48- and K63-linked ubiquitin chains (Fig. 6A). OTUD4 overexpression reduced overall BCL3 ubiquitination (Fig. 6B), with a marked decrease in K63-linked polyubiquitination (Fig. 6C) and a comparatively modest effect on K48-linked chains (Supplementary Fig. 3A). Conversely, OTUD4 depletion increased K63-linked polyubiquitination of BCL3 (Fig. 6D). K63-specific Halo-NZF2 pull-down assays further indicated that OTUD4 WT, but not the catalytically inactive C45A mutant, reduced K63-linked polyubiquitination of endogenous BCL3 in A375 cells (Fig. 6E), without significantly altering total BCL3 protein levels (Supplementary Fig. 3B). These data indicate that OTUD4 preferentially counteracts K63-linked polyubiquitination of BCL3 in a catalytic activity-dependent manner.

To examine how OTUD4 and CYLD regulate BCL3 ubiquitination, we compared K63-linked polyubiquitination of BCL3 in cells expressing CYLD, OTUD4, or both proteins. CYLD reduced K63-linked polyubiquitination of endogenous BCL3 in A375 cells (Fig. 6F) and of exogenous Flag-BCL3 in HEK293T cells (Fig. 6G). Because CYLD has been reported to remove K63-linked polyubiquitin chains from BCL3 in a catalytic activity-dependent manner [9, 64], we compared CYLD WT with catalytically inactive CYLD C598S in the Flag-BCL3 ubiquitination assay. CYLD WT reduced BCL3 K63-linked polyubiquitination more effectively than CYLD C598S, whereas OTUD4 also reduced this modification in cells co-expressing CYLD C598S (Fig. 6G). In OTUD4-deficient cells, re-expression of CYLD attenuated the increase in BCL3 K63-linked polyubiquitination (Fig. 6H). Conversely, OTUD4 retained the ability to reduce BCL3 K63-linked polyubiquitination in CYLD-depleted cells, and co-expression of CYLD further decreased this modification (Supplementary Fig. 3C). Collectively, these data indicate that OTUD4 limits BCL3 K63-linked polyubiquitination through CYLD-dependent and CYLD-independent mechanisms.

### OTUD4 modulates BCL3 nuclear accumulation and Cyclin D1 upregulation in melanoma cells

**Fig. 7:**
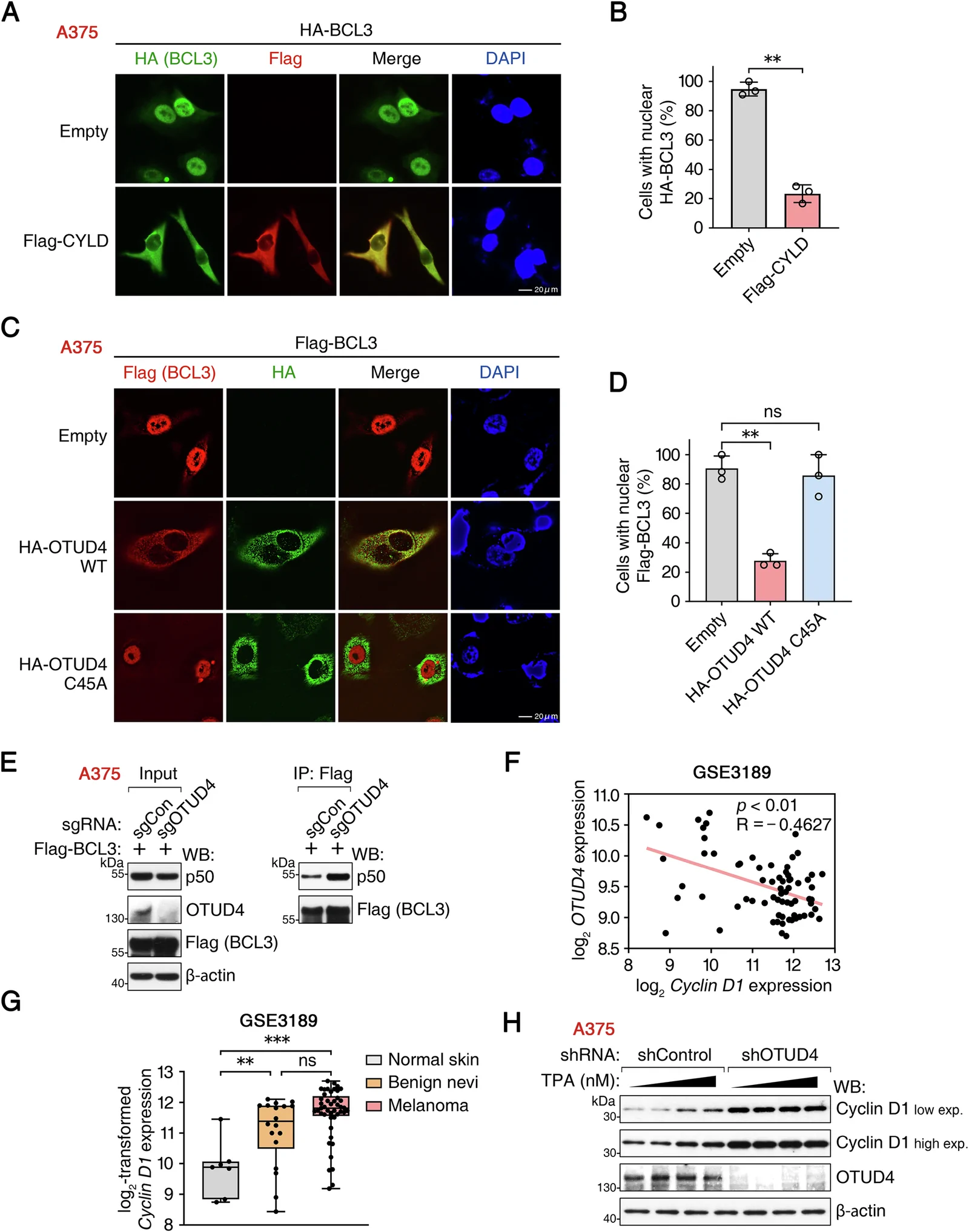
OTUD4 modulates BCL3 nuclear accumulation and Cyclin D1 upregulation in melanoma cells. A375 cells were transfected with HA-BCL3 and Flag-CYLD, and nuclei were visualized by DAPI staining. Representative immunofluorescence images are shown (**A**), and cells with nuclear HA-BCL3 were quantified (**B**). *n* = 30 cells per condition. Scale bar, 20 μm. A375 cells were transfected with Flag-BCL3 together with HA-OTUD4 WT or C45A. Representative immunofluorescence images are shown (**C**), and cells with nuclear Flag-BCL3 were quantified (**D**). Scale bar, 20 μm. **E** OTUD4 knockout enhances the association of BCL3 with p50. A375 cells expressing Flag-BCL3 were subjected to Flag IP followed by WB. **F** OTUD4 expression inversely correlates with the BCL3 target gene *Cyclin D1* in melanoma. Pearson correlation analysis was performed using the GSE3189 dataset (*n* = 70). **G** *Cyclin D1* mRNA expression in normal skin, benign nevi, and melanoma from the GSE3189 dataset was analyzed by one-way ANOVA followed by Tukey’s multiple comparisons test. **H** A375 cells expressing control or OTUD4 shRNA were treated with TPA for the indicated times and analyzed by WB. Data are mean ± SD. Statistical significance was determined using a two-tailed unpaired Student’s *t* test unless otherwise indicated. **p < 0.01, ***p < 0.001. ns, not significant. Experiments were independently repeated three times (**A**, **C**) or twice (**E**, **H**), with similar results.

The CYLD–BCL3 axis was first characterized in normal keratinocytes [9]. To determine whether CYLD also regulates BCL3 localization in melanoma cells, Flag-CYLD was expressed in A375 cells. CYLD overexpression redistributed HA-BCL3 from the nucleus to the cytoplasm and reduced the fraction of cells with nuclear BCL3 (Fig. 7A, B). Similarly, co-expression of OTUD4 WT with Flag-BCL3 reduced the fraction of cells with nuclear Flag-BCL3, whereas the catalytically inactive C45A mutant had minimal effect (Fig. 7C, D). Subcellular fractionation further showed that OTUD4 deficiency increased endogenous BCL3 levels in the nuclear fraction with a corresponding reduction in the cytoplasmic fraction in A375 cells, and this nuclear–cytoplasmic redistribution was more pronounced following 12-O-tetradecanoylphorbol-13-acetate (TPA) treatment (Supplementary Fig. 4A, B), a well-established skin tumor promoter widely used to induce BCL3 nuclear signaling in skin-derived cells [9]. These results indicate that OTUD4 deubiquitinase activity suppresses nuclear accumulation of BCL3 under basal conditions and after TPA stimulation in melanoma cells.

To evaluate downstream consequences of OTUD4-dependent control of BCL3 nuclear localization, we analyzed BCL3 association with NF-κB subunits and Cyclin D1 expression. OTUD4 knockout increased the association of Flag-BCL3 with p50 in A375 cells (Fig. 7E), consistent with increased nuclear BCL3 available for NF-κB complex formation. Analysis of patient-derived transcriptomic data (GSE3189) revealed an inverse correlation between *OTUD4* expression and *Cyclin D1*, a known BCL3 target gene (Fig. 7F), and *Cyclin D1* mRNA levels were higher in melanoma than in benign nevi or normal skin (Fig. 7G), suggesting the clinical relevance of this regulatory relationship. Consistent with these observations, OTUD4 knockdown increased Cyclin D1 protein levels at baseline using two independent shRNAs in A375 cells, and this elevation persisted throughout the time course following TPA treatment (Fig. 7H and Supplementary Fig. 4C). Collectively, these data indicate that OTUD4-dependent regulation of BCL3 ubiquitination limits nuclear accumulation of BCL3 and maintains lower Cyclin D1 levels under basal and inducible conditions in melanoma cells.

### The OTUD4–CYLD–BCL3 axis regulates melanoma cell proliferation and tumor growth

**Fig. 8:**
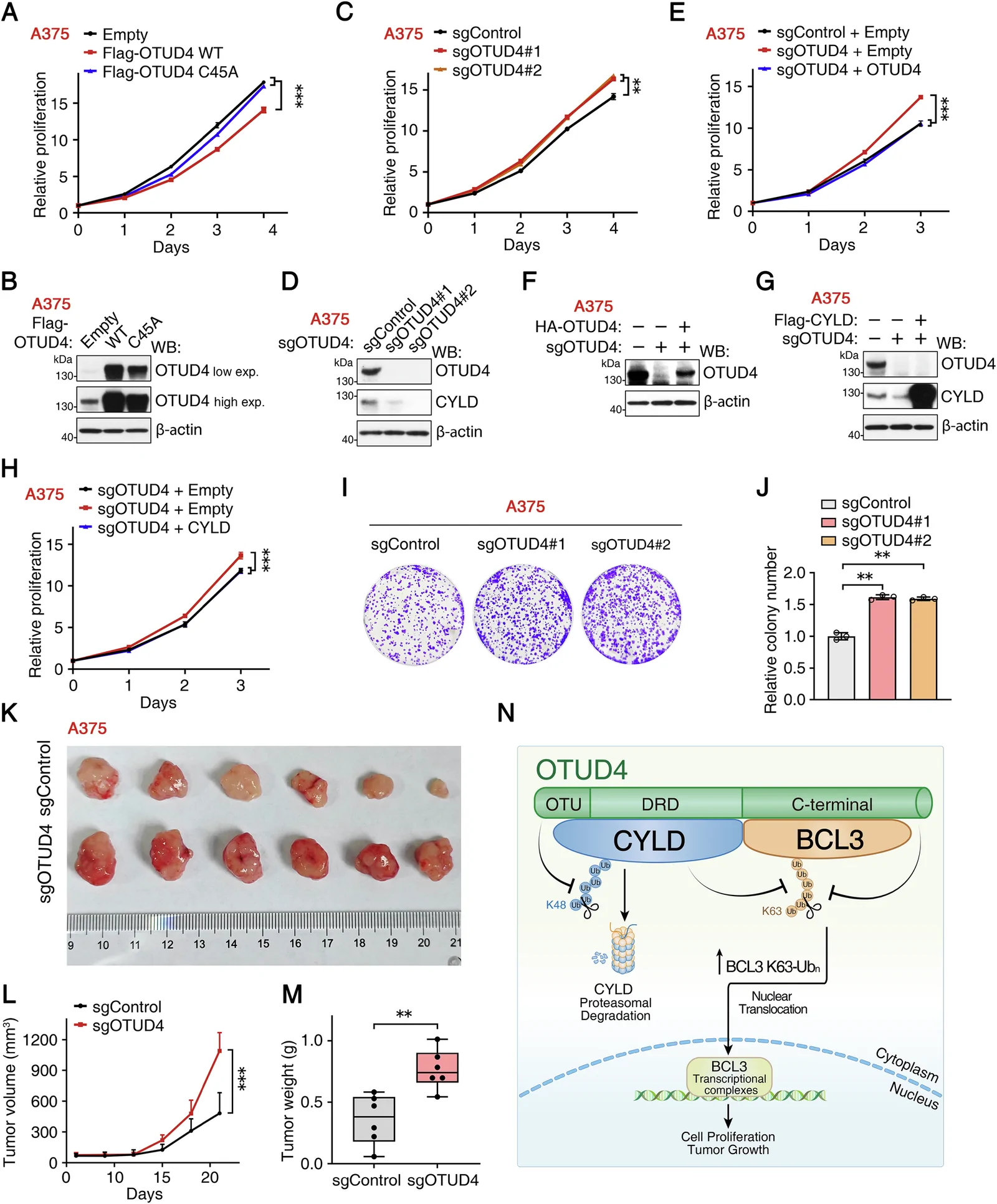
OTUD4–CYLD–BCL3 axis regulates melanoma cell proliferation and tumor growth. **A**, **B** A375 cells were transduced with lentiviral vectors expressing empty Flag vector, Flag-OTUD4 WT, or Flag-OTUD4 C45A, and cell proliferation was measured by CCK-8 assay (**A**). Whole-cell lysates were analyzed by WB (**B**). **C** Increased cell proliferation in OTUD4-knockout A375 cells was measured by CCK-8 assay. **D** WB analysis of whole-cell lysates from OTUD4-knockout A375 cells used in (**C**, **I**). **E**, **F** OTUD4 re-expression suppresses cell proliferation in OTUD4-knockout A375 cells. A375 cells transduced with sgControl or sgOTUD4 were analyzed in the presence or absence of Flag-OTUD4 expression, as indicated. Cell proliferation was measured by CCK-8 assay (**E**). Whole-cell lysates were analyzed by WB (**F**). OTUD4-knockout A375 cells were transduced with empty vector or Flag-CYLD. CYLD re-expression was verified by WB (**G**), and cell proliferation was assessed by CCK-8 assay (**H**). Enhanced clonogenic growth of OTUD4-knockout A375 cells. Representative colony formation images (**I**) and quantification (**J**) are shown. K–M OTUD4 loss promotes melanoma growth in vivo. Control or OTUD4-knockout A375 cells were implanted subcutaneously into nude mice. Representative tumor images (**K**), tumor growth curves (**L**), and tumor weights on day 21 (**M**) are shown (*n* = 6 mice per group). **N** Schematic model showing how the dual deubiquitinase activities of OTUD4 reduce BCL3 K63-linked polyubiquitination, limit BCL3 nuclear accumulation, and suppress melanoma progression. The OTU, DRD, and C-terminal domains correspond to amino acids 1–180, 181–550, and 551–C terminus of OTUD4, respectively. Data are mean ± SD. Statistical significance in **A**, **C**, **E**, **H**, **J** was determined using a two-tailed unpaired Student’s *t* test from biologically independent samples. **p < 0.01, ***p < 0.001. Experiments in **B**, **D**, **F**, **G**, **I** were independently repeated twice with similar results.

To assess the functional contribution of OTUD4 to melanoma growth, we transduced A375 cells with Flag-OTUD4 WT or the catalytically inactive OTUD4 C45A mutant, and cell proliferation was measured using CCK-8 assays. OTUD4 WT significantly reduced cell proliferation, whereas the C45A mutant had no detectable effect (Fig. 8A, B). Conversely, OTUD4-knockout A375 cells showed increased proliferation (Fig. 8C, D), and this increase was reversed by re-expression of OTUD4 (Fig. 8E, F). Similarly, re-expression of CYLD in OTUD4-deficient cells also reduced proliferation (Fig. 8G, H). In parallel, depletion of BCL3 reduced proliferation, whereas re-expression of BCL3 restored proliferation (Supplementary Fig. 5A–C). Furthermore, BCL3 knockdown in OTUD4-deficient cells attenuated the increased proliferation caused by OTUD4 loss (Supplementary Fig. 5D, E). These data indicate that OTUD4 constrains melanoma cell proliferation, with CYLD functioning downstream in this regulatory pathway.

To examine long-term growth capacity, we analyzed clonogenic growth and tumor formation. OTUD4-deficient A375 cells showed enhanced clonogenic growth (Fig. 8I, J). BCL3 knockdown reduced colony formation, and re-expression of BCL3 restored colony formation in BCL3-knockdown cells (Supplementary Fig. 5F–I). BCL3 knockdown also attenuated the increase in clonogenic growth induced by OTUD4 loss (Supplementary Fig. 5J, K). Tumor growth was further assessed using subcutaneous xenografts in male athymic nude (nu/nu) mice, in which tumors derived from OTUD4-knockout A375 cells grew more rapidly and reached greater final volumes and weights than control tumors (Fig. 8K–M). Collectively, these data support a model (Fig. 8N) in which OTUD4 functions as an upstream regulator of the CYLD–BCL3 axis and also directly modulates BCL3, thereby constraining melanoma cell proliferation and tumor growth.

## Discussion

Here, we identify *OTUD4* as a melanoma-associated regulator that is progressively decreased during melanocytic transformation (Fig. 1). OTUD4 directly binds and stabilizes CYLD by removing K48-linked polyubiquitin chains in an activity-dependent manner (Figs. 2–4), and stabilized CYLD further contributes to the suppression of BCL3 ubiquitination. In parallel, OTUD4 counteracts K63-linked polyubiquitin chains of BCL3 independently of CYLD (Figs. 5, 6). Functionally, OTUD4 limits nuclear accumulation of BCL3 and the expression of the BCL3 target gene Cyclin D1 in melanoma cells (Fig. 7). OTUD4 depletion increases melanoma cell proliferation and tumor growth in clonogenic assays and in xenograft models (Fig. 8). Together, these results position OTUD4 within a previously unrecognized OTUD4–CYLD–BCL3 regulatory axis that suppresses melanoma progression (Fig. 8N).

A key mechanistic insight from this study is that OTUD4 directly deubiquitinates and stabilizes CYLD through a canonical, catalytically active deubiquitinase (DUB) mechanism. CYLD is regulated at multiple levels, including transcriptional control in inflammatory and tumor contexts [10, 65], MALT1-dependent proteolytic cleavage during receptor signaling [66, 67], and ubiquitin-mediated degradation catalyzed by E3 ligases such as MIB2 and β-TRCP [37–41]. However, the regulation of CYLD protein stability by deubiquitinases has remained unexplored. Our data indicate that OTUD4 directly binds CYLD via the N-terminal region of OTUD4 and removes K48-linked polyubiquitin chains from CYLD in a catalytic activity–dependent manner, leading to stabilization of CYLD protein levels (Figs. 2–4). In contrast, an activity-independent, noncanonical mode of OTUD4 function was originally characterized in our 2015 *EMBO Journal* study [50], in which the deubiquitinase-recruitment domain (DRD) of OTUD4 recruits other DUBs, including USP7 and USP9X, thereby promoting substrate deubiquitination. Subsequent studies have provided additional evidence for this noncanonical mechanism [51, 52], suggesting that this represents a recurrent mode of OTUD4 regulation. Unlike the noncanonical OTUD4 function, CYLD stabilization requires the intrinsic catalytic activity of OTUD4 (Figs. 2–4), indicating a distinct and previously underexplored mode of DUB–DUB regulation. Domain-mapping analyses further show that CYLD binds to OTUD4 residues 1–550 (Fig. 2H, I), a region encompassing the DRD (residues 181–550). This spatial overlap, together with the lack of effect of USP7 or USP9X depletion on CYLD stabilization (Supplementary Fig. 2C, D), suggests that CYLD binding may favor OTUD4 catalytic activity while limiting DRD-dependent recruitment of other DUBs, thereby pointing to a canonical, catalytic mode of OTUD4 action toward CYLD.

In parallel with the canonical stabilization of CYLD, OTUD4 directly regulates BCL3 through a distinct K63-linked deubiquitination mechanism. The ability of OTUD4 to hydrolyze K63-linked polyubiquitin chains was first described in our 2018 *Molecular Cell* study [53]. K63-linked polyubiquitination of BCL3 promotes nuclear translocation and transcriptional activity [9, 29]. In this study, OTUD4 directly binds BCL3 through the C-terminal region and counteracts BCL3 K63-linked polyubiquitination, limiting nuclear accumulation of BCL3 and downstream transcriptional output, as reflected by Cyclin D1 expression (Figs. 5–7 and Supplementary Fig. 4). This distinction between signaling-linked and degradation-linked ubiquitination is relevant because BCL3 protein levels are frequently elevated in tumors and associated with poor prognosis, including in melanoma [68, 69]. Our data provide the first evidence that BCL3 also undergoes K48-linked polyubiquitination (Fig. 6A), a canonical signal for proteasome-dependent degradation [70]. Although previous studies have identified E3 ligases such as TBLR1 as regulators of BCL3 stability [29, 30], the ubiquitin chain types that control BCL3 stability and signaling remain incompletely characterized. This study connects these observations by distinguishing degradation-linked K48-linked polyubiquitination from signaling-associated K63-linked polyubiquitination of BCL3. In addition, CYLD has been reported to remove K63-linked polyubiquitin chains from BCL3 [9, 29, 64], and OTUD4-mediated stabilization of CYLD may further suppress K63-linked polyubiquitination of BCL3 (Fig. 6). Consistent with a primary role in K63-linked BCL3 regulation, OTUD4 had only a limited and context-dependent effect on BCL3 protein stability: in HEK293T cells, OTUD4 overexpression was associated with a modest reduction in K48-linked polyubiquitination of Flag-BCL3, whereas in A375 cells, OTUD4 depletion did not detectably alter endogenous BCL3 protein levels (Supplementary Fig. 3A, B). Together, these results indicate that OTUD4 limits BCL3 K63-linked polyubiquitination through direct deubiquitination of BCL3 and CYLD stabilization via K48-linked deubiquitination.

Clinically, melanoma datasets show that *OTUD4* and *CYLD* expression progressively decrease from normal skin through benign nevi to melanoma, with a further reduction in highly metastatic tumors (Fig. 1 and Supplementary Fig. 1). Low expression of *OTUD4* and *CYLD* is associated with worse survival in melanoma patients (Fig. 1G and Supplementary Fig. 1G), indicating disruption of an OTUD4–CYLD regulatory axis during melanoma progression. Functionally, loss of OTUD4 reduces CYLD stability, promotes BCL3 nuclear accumulation, and elevates downstream transcriptional targets, including Cyclin D1 (Fig. 7 and Supplementary Fig. 4). These molecular changes are associated with increased cell proliferation, clonogenic capacity, and xenograft tumor growth (Fig. 8 and Supplementary Fig. 5), supporting a model in which melanoma cells benefit from the loss of OTUD4-dependent control over both K48- and K63-linked ubiquitin pathways, thereby enhancing BCL3–NF-κB signaling (Fig. 8N). In contrast, previous studies have demonstrated that OTUD4 regulates canonical NF-κB signaling by modulating K63-linked polyubiquitination of MYD88 and TAK1 [53, 54]. The present study extends this regulatory mechanism to BCL3-dependent, noncanonical NF-κB signaling, revealing a broader role of OTUD4 in the NF-κB pathway. Importantly, CYLD is frequently downregulated in melanoma, where it functions as a tumor suppressor [10, 32, 34, 62, 71]. The coordinated reduction of OTUD4 and CYLD across melanoma datasets indicates a DUB–DUB regulatory relationship in which disruption of interconnected ubiquitin-regulatory nodes releases negative control of BCL3–NF-κB signaling and promotes tumor progression.

In summary, this study identifies an OTUD4–CYLD–BCL3 regulatory axis in melanoma, in which OTUD4 stabilizes CYLD by removing K48-linked polyubiquitin chains and independently counteracts K63-linked polyubiquitination of BCL3. Through these chain-specific activities, OTUD4 restrains BCL3-associated transcriptional programs linked to melanoma progression, consistent with the coordinated downregulation of *OTUD4* and *CYLD* in melanoma patient datasets. Several regulatory questions remain unresolved, including the upstream signals that reduce OTUD4 expression during melanoma progression and whether CYLD feeds back to regulate OTUD4 expression or activity. Previous studies indicate that CYLD-dependent control of BCL3 involves HDAC6 inhibition, increased tubulin acetylation, and perinuclear CYLD relocalization, whereas IKKε suppresses CYLD deubiquitinase activity [72, 73]. In parallel, OTUD4 phosphorylation activates the K63-linked deubiquitinase activity of OTUD4 [53]. These observations indicate that kinase-dependent signaling and subcellular localization may regulate the OTUD4–CYLD–BCL3 axis. Recent studies using small-molecule BCL3 inhibitors further show that pharmacological inhibition of BCL3 reduces cyclin D1 expression and suppresses melanoma cell proliferation, migration, and tumor growth, consistent with a functional contribution of BCL3 to melanoma progression [74, 75]. Future studies using melanoma patient specimens, complementary models, and pharmacological inhibition of BCL3 may clarify how this axis is altered during melanoma progression.

## Supplementary information

Supplemental Figures S1-5 and Legends

Uncropped WB files

## Data Availability

All data supporting the findings of this study are available within the article and its Supplementary Information.
