# Supplementary material for: OTUD4 stabilizes CYLD and counteracts BCL3 K63-linked polyubiquitination to suppress melanoma progression
Source: Cell Death Dis. 2026 Jul 28;17(1):904. doi: 10.1038/s41419-026-09130-0 (PMC13691072; doi:10.1038/s41419-026-09130-0)
Supplement: Supplementary file 1 [file 41419_2026_9130_MOESM1_ESM.pdf]

SUPPLEMENTARY INFORMATION

OTUD4 stabilizes CYLD and counteracts BCL3 K63-linked polyubiquitination to suppress melanoma progression

Yuming He [1,4], Lin Miao [1,4], Yunfang Deng [1], Yuyao Xue [1], Zhaohui Liu [1], Zhiming Sun [1], Chujing He [1], Hao Feng [3], Jiabing Li [1], Yuqiao Cai [1], Yaoyu Gu [1], Ke Li [3] & Yu Zhao [1,2*]

[1] The National & Local Joint Engineering Laboratory of Animal Peptide Drug Development, College of Life Sciences, Hunan Normal University, Changsha, Hunan, 410081, China

[2] Peptide and Small Molecule Drug R&D Platform, Furong Laboratory, Hunan Normal University, Changsha, Hunan, 410081, China

[3] Department of Dermatology, Hunan Provincial People's Hospital (The First Affiliated Hospital of Hunan Normal University), Changsha, Hunan, 410000, China

* Corresponding author: Yu Zhao (smile_zhao@hotmail.com)

[4] These authors contributed equally: Yuming He, Lin Miao.

Running title: OTUD4 stabilizes CYLD and counteracts BCL3 ubiquitination

Keywords: OTUD4; CYLD; BCL3; ubiquitination; melanoma progression

SUPPLEMENTARY FIGURE LEGENDS

Supplementary Fig. 1 Coordinated expression patterns of *CYLD* and *OTUD4* across melanoma-related datasets. A, B *OTUD4* levels positively correlate with *CYLD* expression in normal skin (**A**, GTEx; n = 557; R = 0.49) and melanoma (**B**, TCGA-SKCM; n = 461; R = 0.52). Pearson correlation analysis was performed using GEPIA based on GTEx and TCGA datasets ($p < 0.001$). **C** *CYLD* mRNA levels in normal skin and melanoma based on TNMplot analysis (Mann–Whitney *U* test). **D** *CYLD* mRNA levels in normal skin, benign nevi, and melanoma from the GEO dataset GSE3189 were analyzed by one-way ANOVA followed by Tukey's multiple comparisons test. **E** *CYLD* mRNA levels in poorly metastatic and highly metastatic melanoma cell lines from the GEO dataset GSE7929. **F** *CYLD* mRNA levels in sun-exposed and non–sun-exposed skin samples from GTEx data accessed through the Human Protein Atlas database. **G** Kaplan–Meier survival analysis of melanoma patients stratified by *CYLD* expression using GEPIA. **H** CYLD-associated deubiquitinases identified by re-analysis of published CYLD interactome mass spectrometry datasets [1], showing OTUD4 relative to USP10 and USP15. Data are mean ± SD. Statistical significance was determined using a two-tailed unpaired Student's *t*-test unless otherwise indicated. $^{**}p < 0.01$, $^{***}p < 0.001$. ns, not significant.

Supplementary Fig. 2 OTUD4 stabilizes CYLD independently of USP9X and USP7. A, B A375 cells transduced with OTUD4 sgRNA (A) or shRNA (B) were treated with or without MG132 (20 μM) for 4 h before lysis, and whole-cell lysates were analyzed by western blotting (WB). **C, D** A375 cells transduced with lentiviral vectors expressing control shRNA or shRNAs targeting USP9X (C) or USP7 (D) were analyzed by WB. **E, F** A549 (E) or H1299 (F) cells transduced with control shRNA or OTUD4 shRNA were treated with cycloheximide (CHX), and whole-cell lysates were analyzed by WB. All experiments were independently repeated at least twice with similar results.

Supplementary Fig. 3 OTUD4 regulates BCL3 polyubiquitination. A HEK293T cells stably expressing Flag-BCL3 were transfected with HA-Ub(K48-only) with or without OTUD4, followed by denaturing Flag IP and WB. Polyubiquitinated BCL3 was detected using an anti-HA antibody. **B** A375 cells stably expressing control shRNA or OTUD4 shRNAs (#1 and #2) were analyzed by WB using the indicated antibodies. **C** HEK293T cells stably expressing Flag-BCL3 were transduced with CYLD shRNA and transfected with HA-Ub(K63-only) and His-OTUD4, with or without His-CYLD re-expression, followed by denaturing Flag IP and WB. All experiments were independently repeated at least twice with similar results.

Supplementary Fig. 4 OTUD4 depletion increases nuclear accumulation of endogenous BCL3. A, B A375 cells stably expressing control sgRNA or OTUD4 sgRNAs (#1 and #2) were left untreated (A) or treated with TPA (B). Cytoplasmic and nuclear fractions were prepared, and BCL3 distribution was analyzed by WB using the indicated antibodies. GAPDH and histone H3 were used as cytoplasmic and nuclear markers, respectively. The red asterisk denotes endogenous BCL3. **C** OTUD4 knockdown increases Cyclin D1 protein levels. A375 cells transduced with control shRNA or OTUD4 shRNAs (#1 and #2) were analyzed by WB. All experiments were independently repeated at least twice with similar results.

Supplementary Fig. 5 BCL3 is required for melanoma cell proliferation and clonogenic capacity. A Proliferation of A375 melanoma cells transduced with control shRNA or two independent BCL3 shRNAs (#1 and #2) was measured by CCK-8 assay over the indicated time course. **B** BCL3 knockdown efficiency in A375 cells was verified by WB. **C** Proliferation of BCL3-depleted A375 cells reconstituted with HA-BCL3 or empty vector was assessed. **D, E** A375 cells transduced with sgControl or sgOTUD4 were retransduced with shControl or shBCL3, as indicated. Cell proliferation was measured by CCK-8 assay (D). Whole-cell lysates were analyzed by WB (E). **F, G** Clonogenic growth of A375 cells expressing control shRNA or BCL3 shRNAs (#1 and #2) was evaluated by colony formation assay (F), with quantification shown in (G). **H, I**

Clonogenic growth of BCL3-depleted A375 cells reconstituted with HA-BCL3 or empty vector was assessed by colony formation assay (H), with quantification shown in (I). **J, K** Clonogenic growth of cells from (D) was assessed by colony formation assay (J), with quantification shown in (K). Data are mean ± SD from three biologically independent samples. Statistical significance was determined using a two-tailed unpaired Student's *t*-test. ***$p < 0.001$. ns, not significant.

References

1. Elliott PR, Leske D, Hrdinka M, Bagola K, Fiil BK, McLaughlin SH, et al. SPATA2 Links CYLD to LUBAC, Activates CYLD, and Controls LUBAC Signaling. Mol Cell. 2016;63:990-1005.

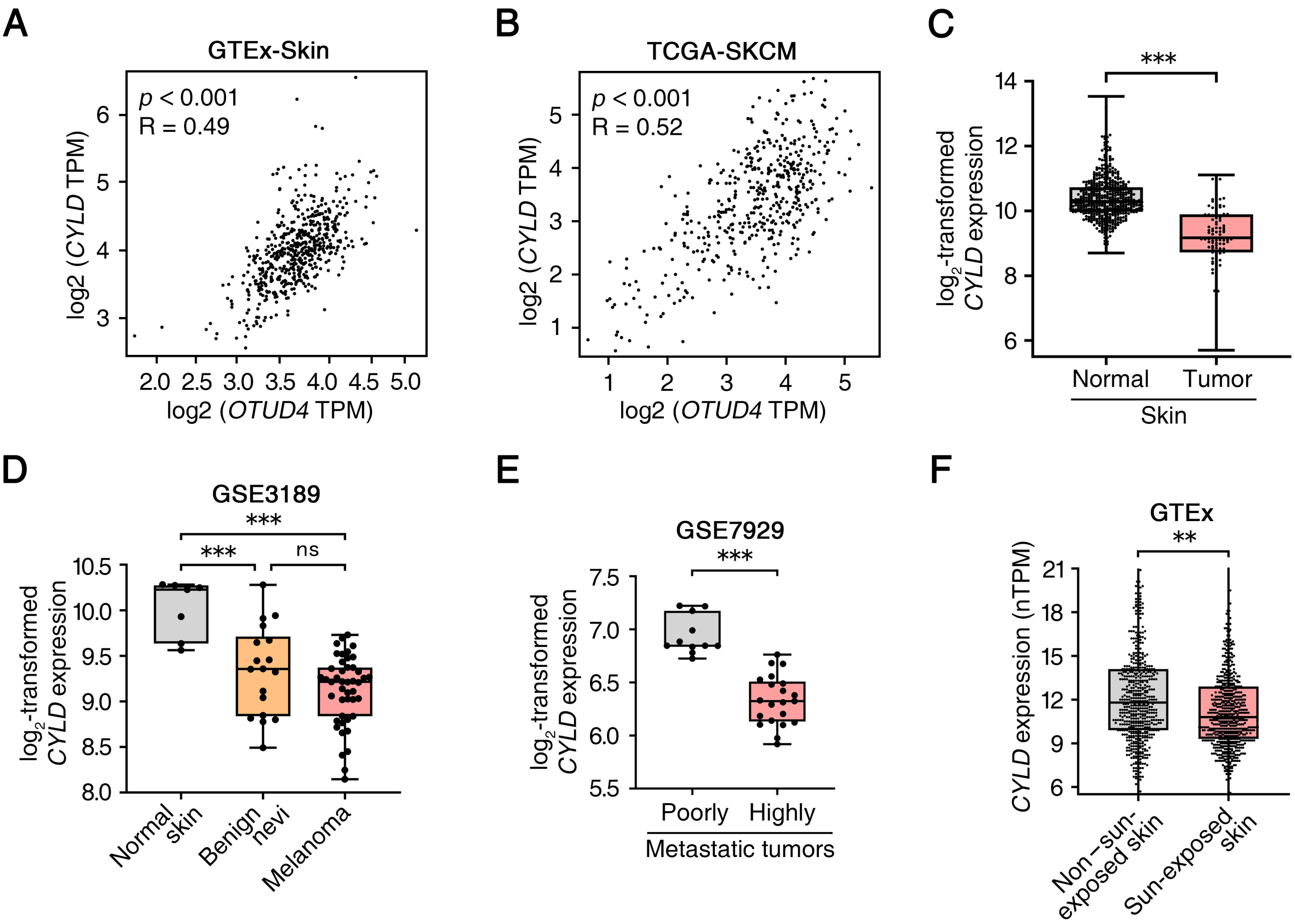

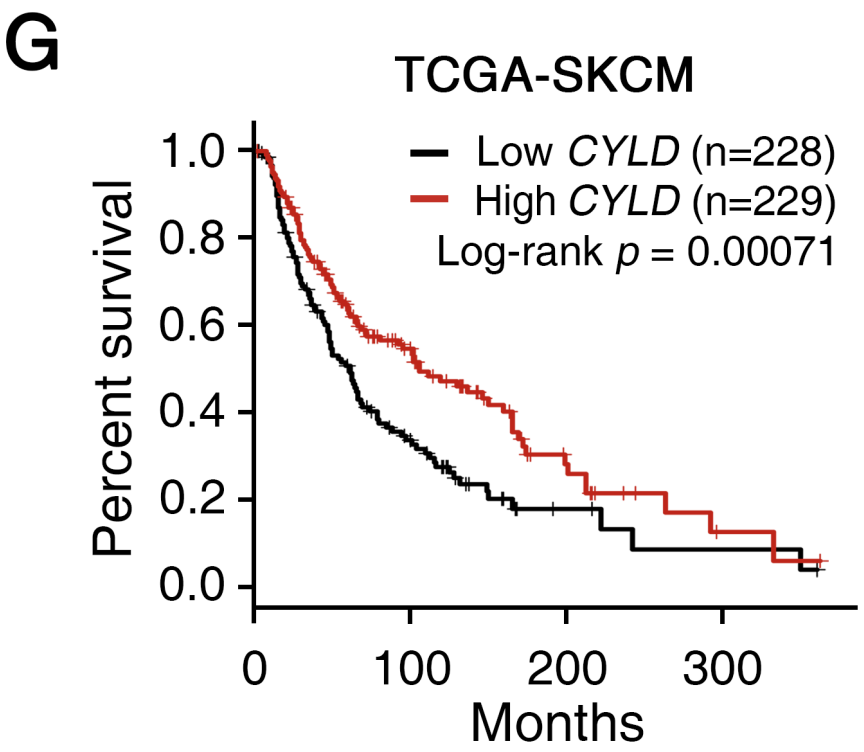

H

Gene name	Peptide count	Unique peptides
OTUD4	30	30
USP10	6	6
USP15	14	14

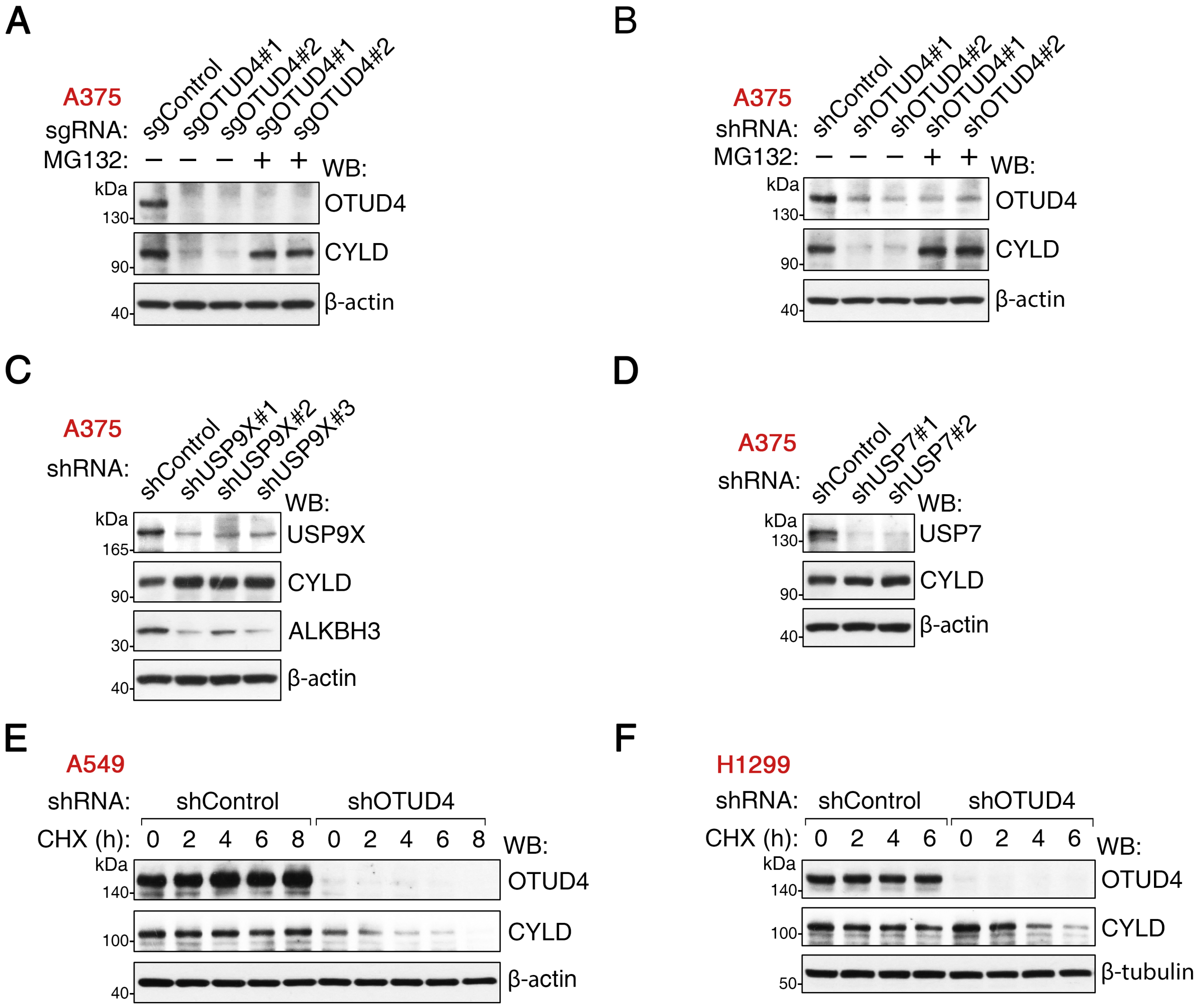
A
A375
sgRNA: sgControl sgOTUD4#1 sgOTUD4#2 sgOTUD4#1 sgOTUD4#2
MG132: – – – + +
WB:
kDa
130
90
40
OTUD4
CYLD
β-actin
B
A375
shRNA: shControl shOTUD4#1 shOTUD4#2 shOTUD4#1 shOTUD4#2
MG132: – – – + +
WB:
kDa
130
90
40
OTUD4
CYLD
β-actin
C
A375
shRNA: shControl shUSP9X#1 shUSP9X#2 shUSP9X#3
WB:
kDa
165
90
30
40
USP9X
CYLD
ALKBH3
β-actin
D
A375
shRNA: shControl shUSP7#1 shUSP7#2
WB:
kDa
130
90
40
USP7
CYLD
β-actin
E
A549
shRNA: shControl shOTUD4
CHX (h): 0 2 4 6 8 0 2 4 6 8
WB:
kDa
140
100
40
OTUD4
CYLD
β-actin
F
H1299
shRNA: shControl shOTUD4
CHX (h): 0 2 4 6 0 2 4 6
WB:
kDa
140
100
50
OTUD4
CYLD
β-tubulin

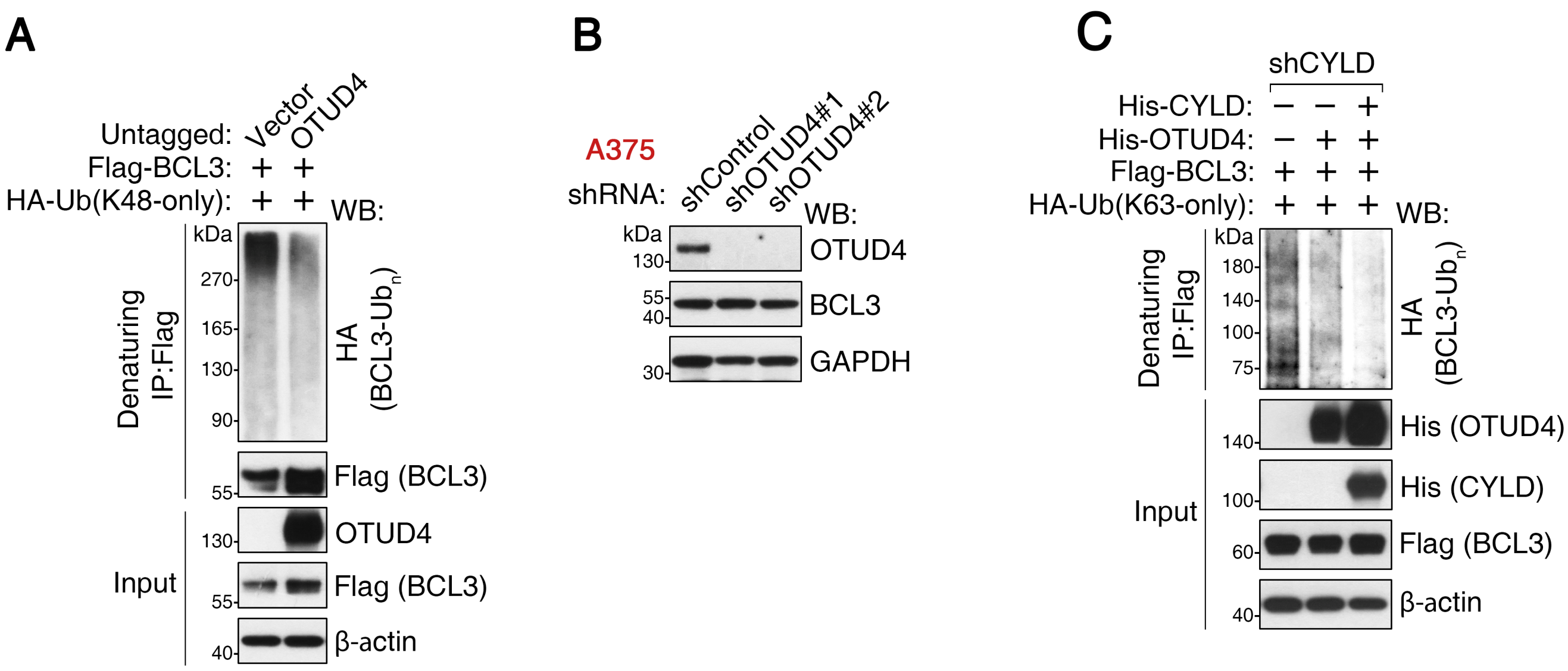
A
Untagged: Vector OTUD4
Flag-BCL3: + +
HA-Ub(K48-only): + +
WB:
Denaturing IP:Flag
kDa
270
165
130
90
HA (BCL3-Ubn)
55
Flag (BCL3)
Input
130
OTUD4
55
Flag (BCL3)
40
β-actin
B
A375
shRNA: shControl shOTUD4#1 shOTUD4#2
WB:
kDa
130
OTUD4
55
40
BCL3
30
GAPDH
C
shCYLD
His-CYLD: − − +
His-OTUD4: − + +
Flag-BCL3: + + +
HA-Ub(K63-only): + + +
WB:
Denaturing IP:Flag
kDa
180
140
100
75
HA (BCL3-Ubn)
Input
140
His (OTUD4)
100
His (CYLD)
60
Flag (BCL3)
40
β-actin

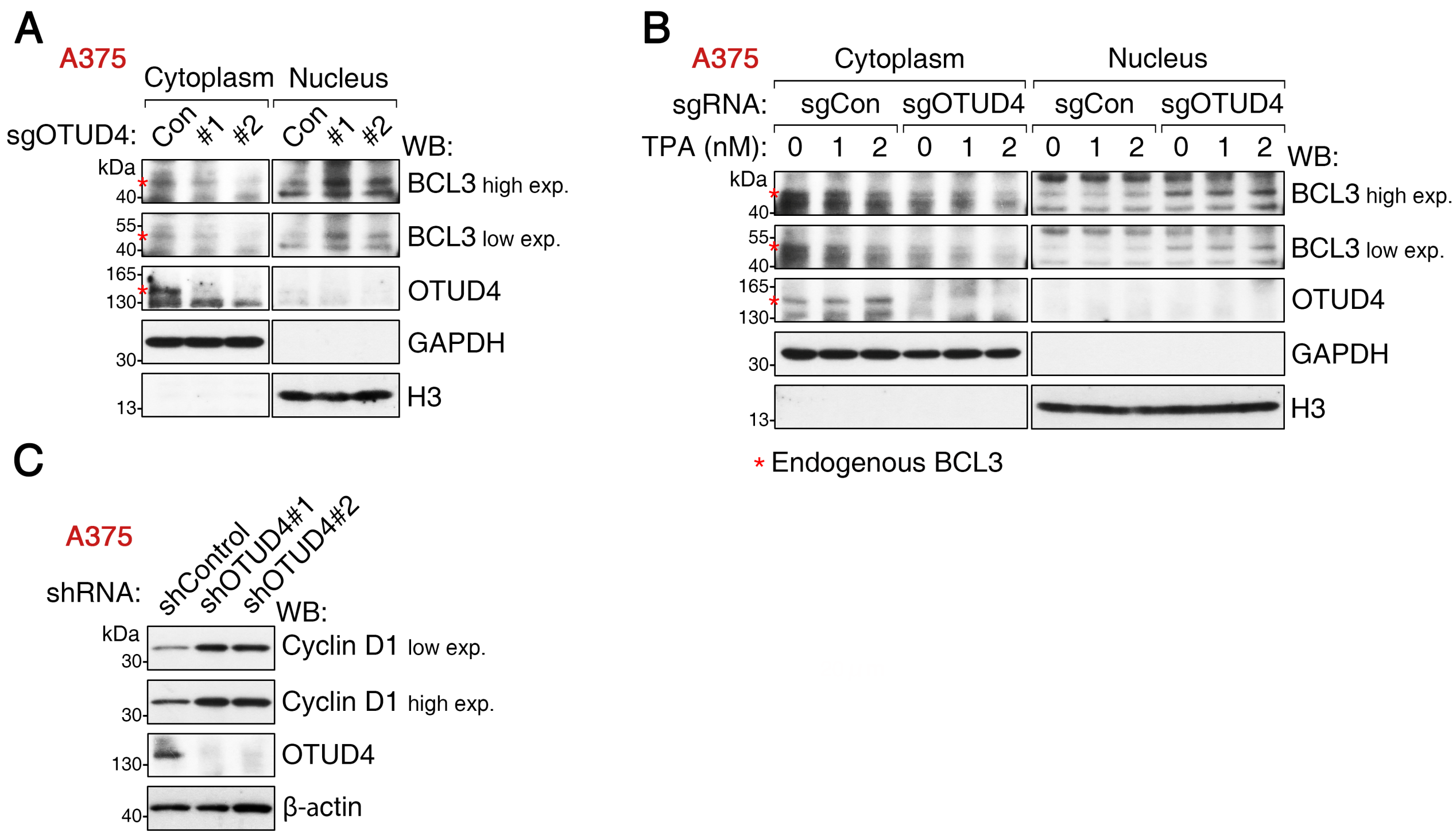
A
A375
Cytoplasm Nucleus
sgOTUD4: Con #1 #2 Con #1 #2
WB:
kDa
40
55
40
165
130
30
13
BCL3 high exp.
BCL3 low exp.
OTUD4
GAPDH
H3
B
A375
Cytoplasm
Nucleus
sgRNA: sgCon sgOTUD4 sgCon sgOTUD4
TPA (nM): 0 1 2 0 1 2 0 1 2 0 1 2
WB:
kDa
40
55
40
165
130
30
13
BCL3 high exp.
BCL3 low exp.
OTUD4
GAPDH
H3
* Endogenous BCL3
C
A375
shRNA: shControl shOTUD4#1 shOTUD4#2
WB:
kDa
30
30
130
40
Cyclin D1 low exp.
Cyclin D1 high exp.
OTUD4
β-actin

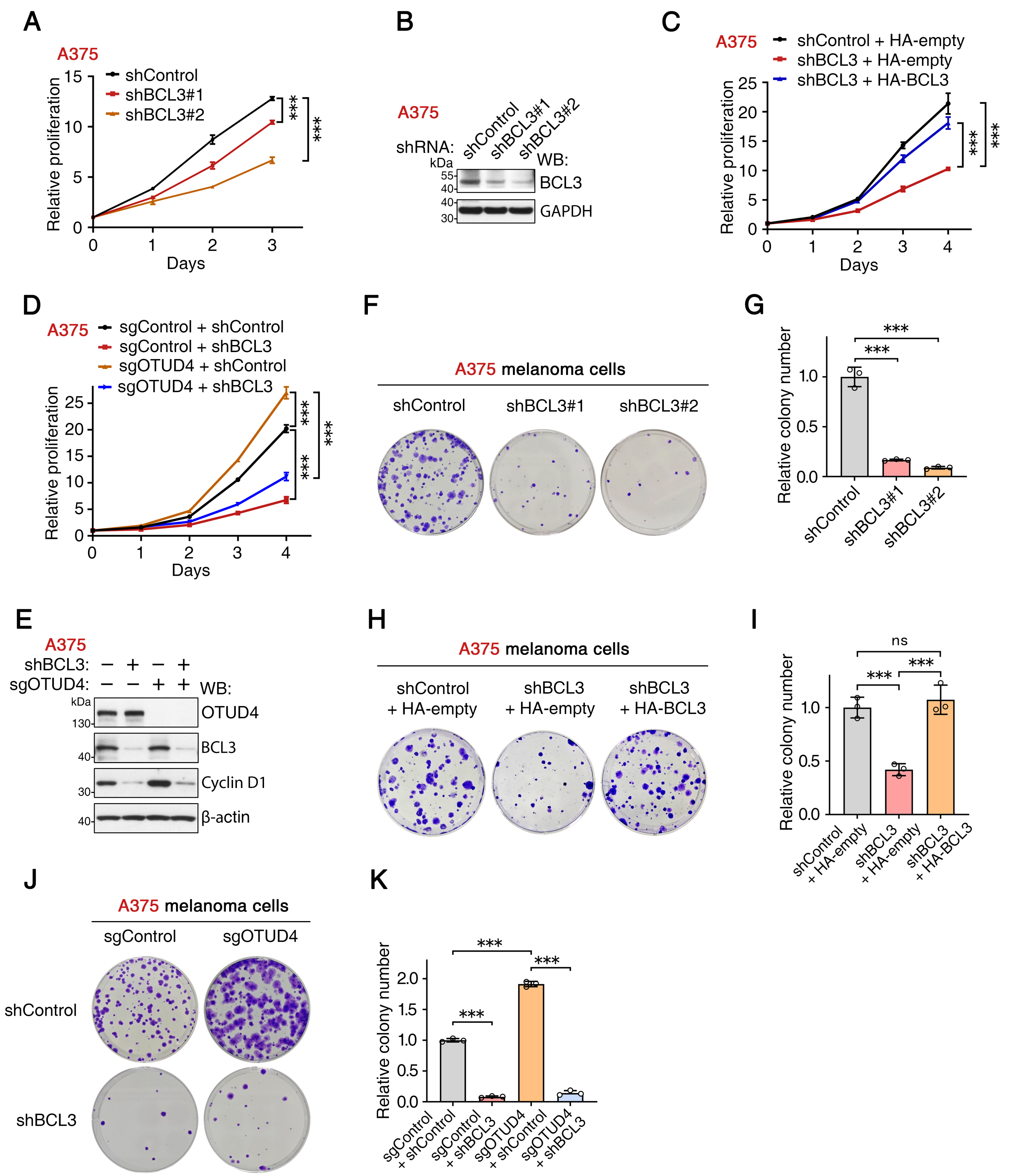
A
A375
shControl
shBCL3#1
shBCL3#2
Relative proliferation
Days
***
B
A375
shRNA:
shControl
shBCL3#1
shBCL3#2
kDa
WB:
BCL3
GAPDH
C
A375
shControl + HA-empty
shBCL3 + HA-empty
shBCL3 + HA-BCL3
Relative proliferation
Days
D
A375
sgControl + shControl
sgControl + shBCL3
sgOTUD4 + shControl
sgOTUD4 + shBCL3
Relative proliferation
Days
F
A375 melanoma cells
shControl
shBCL3#1
shBCL3#2
G
Relative colony number
shControl
shBCL3#1
shBCL3#2
E
A375
shBCL3:
sgOTUD4:
WB:
OTUD4
BCL3
Cyclin D1
β-actin
H
A375 melanoma cells
shControl + HA-empty
shBCL3 + HA-empty
shBCL3 + HA-BCL3
I
ns
Relative colony number
J
A375 melanoma cells
sgControl
sgOTUD4
shControl
shBCL3
K
Relative colony number
sgControl + shControl
sgControl + shBCL3
sgOTUD4 + shControl
sgOTUD4 + shBCL3
