# Supplementary material for: OTUD4 stabilizes CYLD and counteracts BCL3 K63-linked polyubiquitination to suppress melanoma progression
Source: Cell Death Dis. 2026 Jul 28;17(1):904. doi: 10.1038/s41419-026-09130-0 (PMC13691072; doi:10.1038/s41419-026-09130-0)

Figure 2A

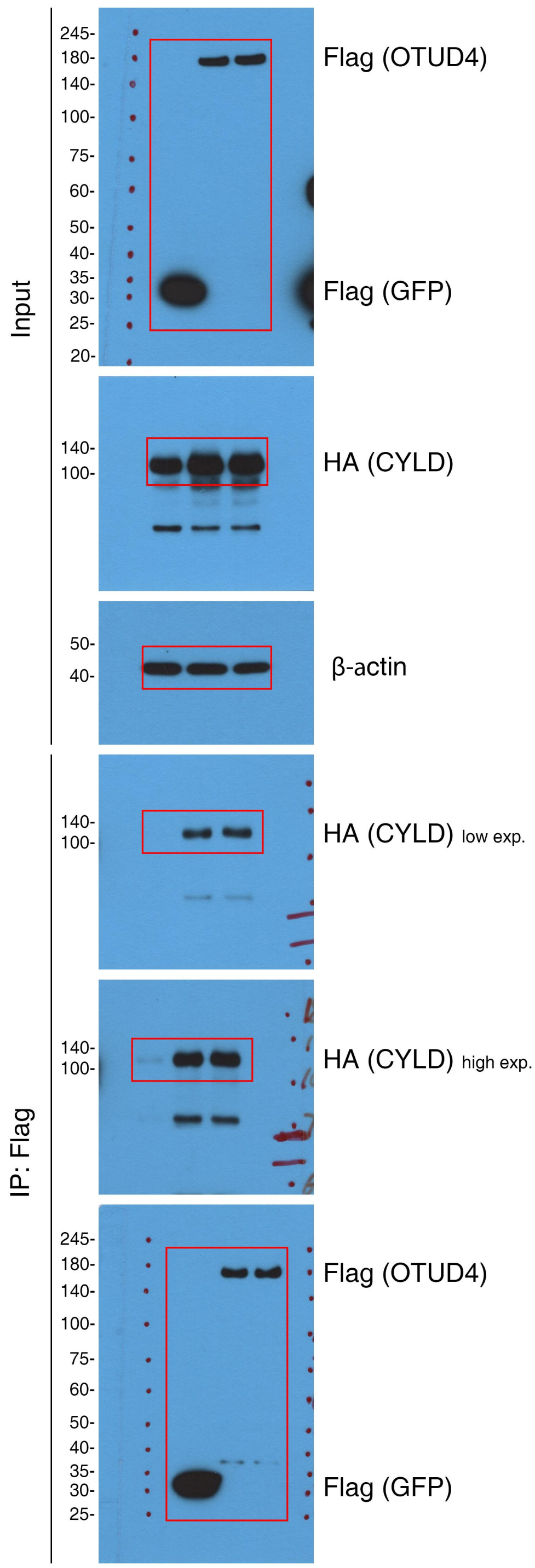

Figure 2B

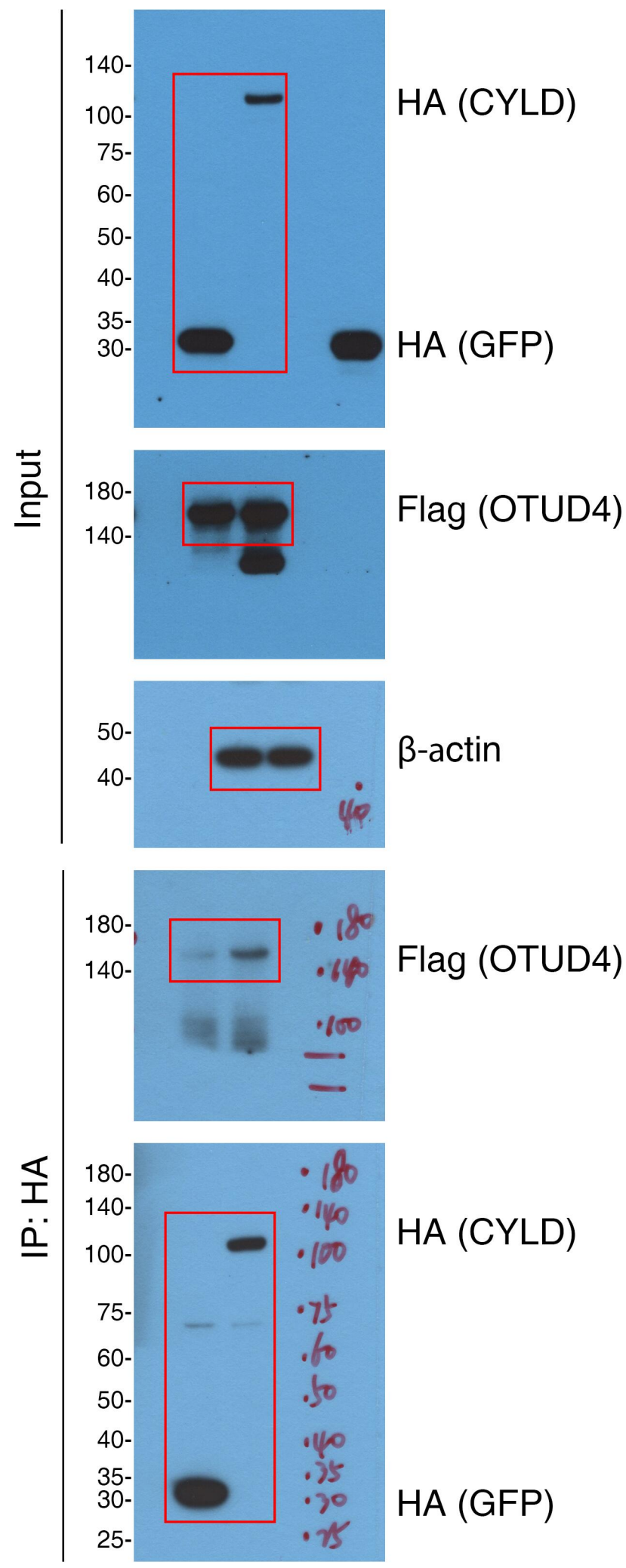

Figure 2C

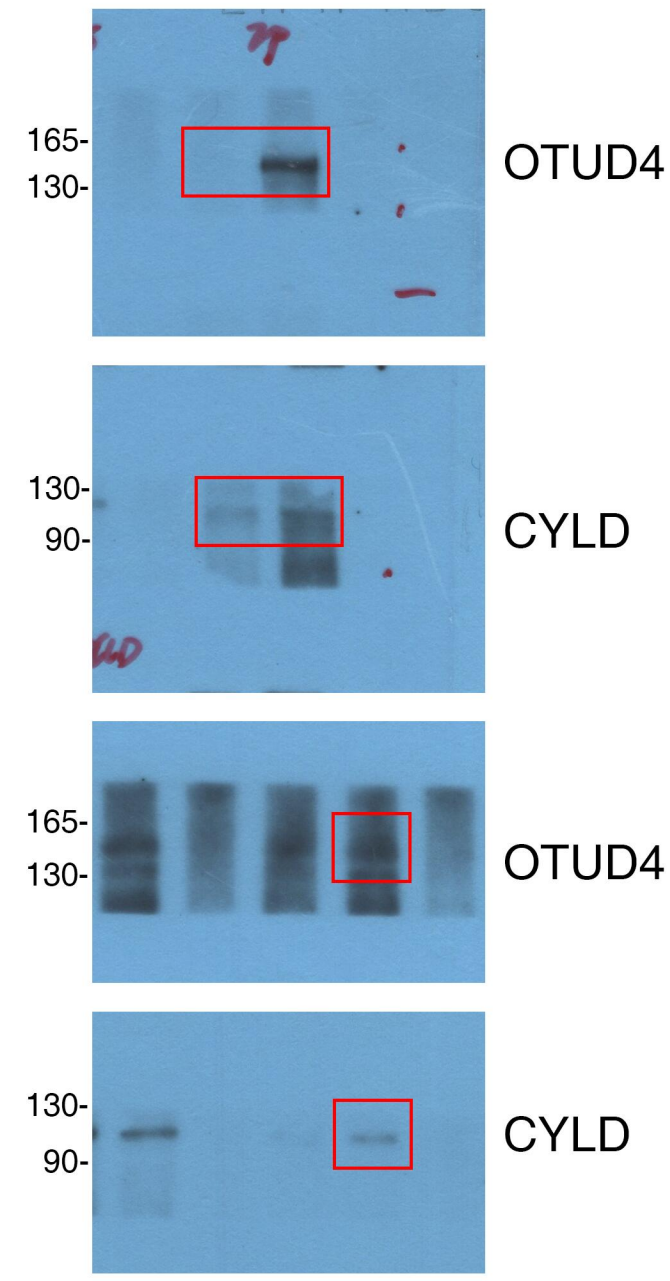

Figure 2D

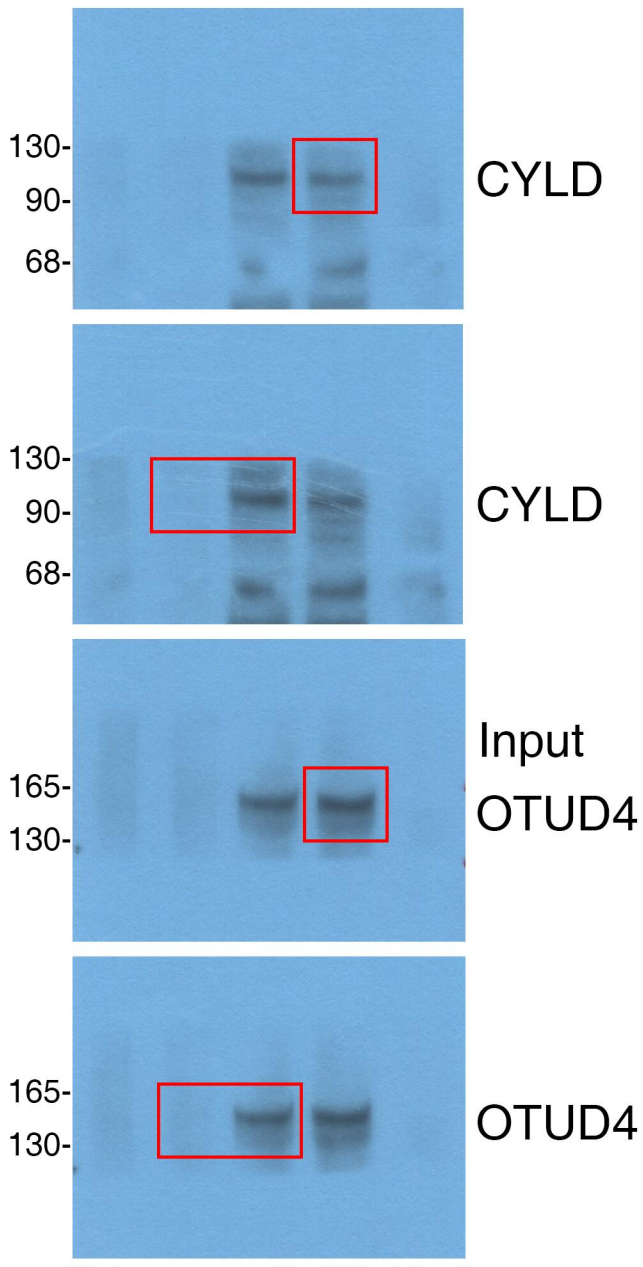

Figure 2E

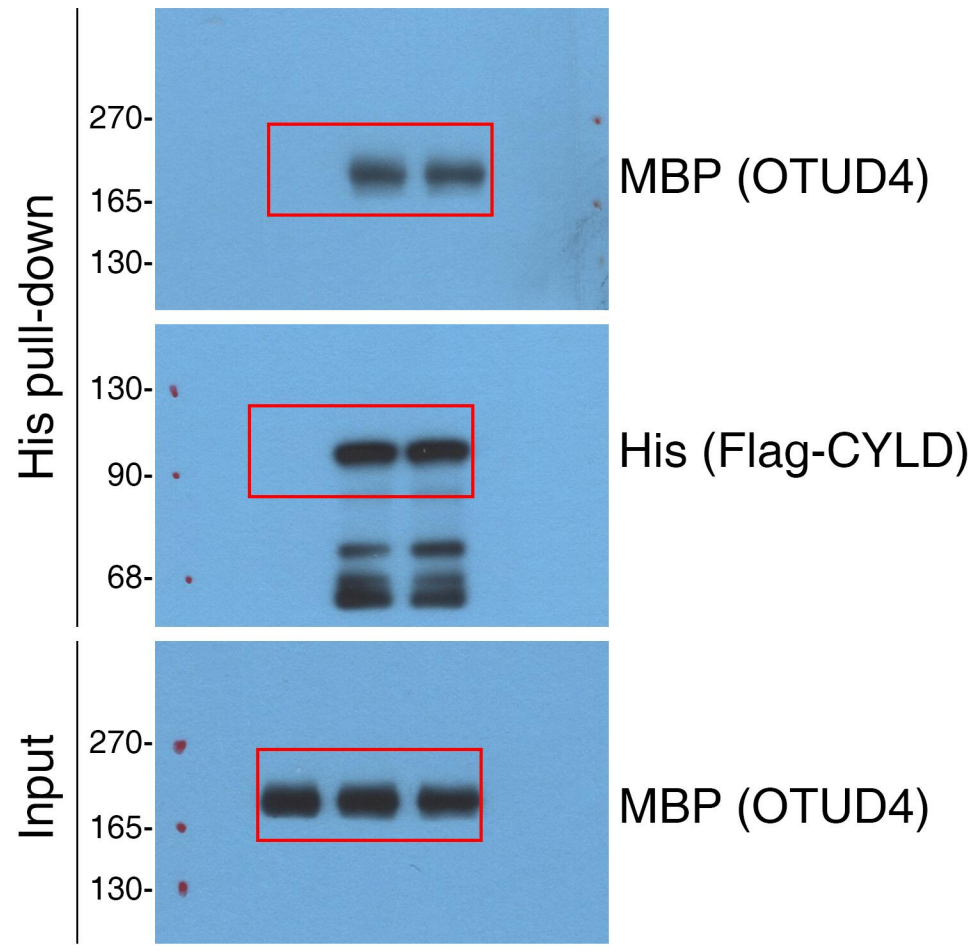

Figure 2F

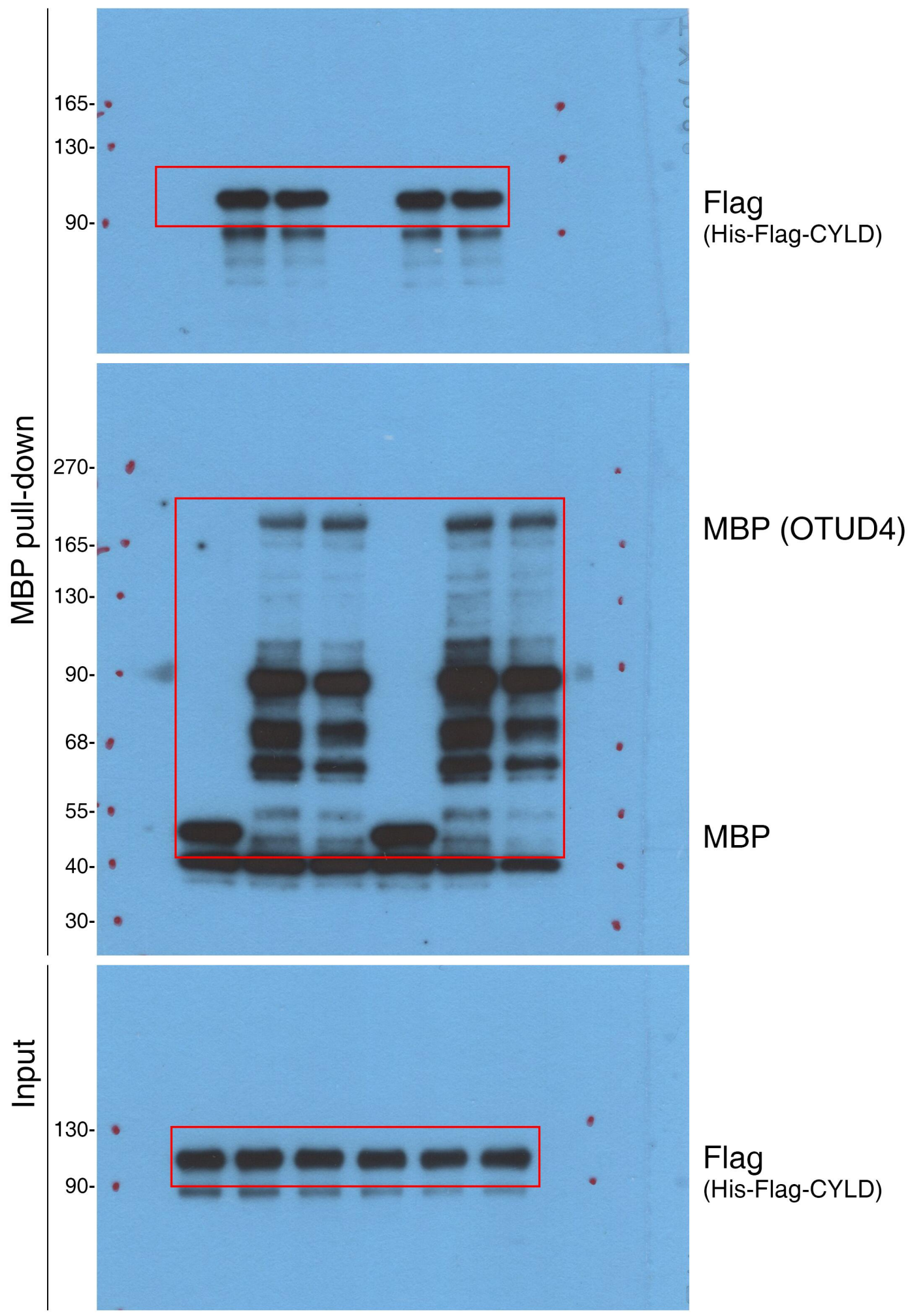

Figure 2I

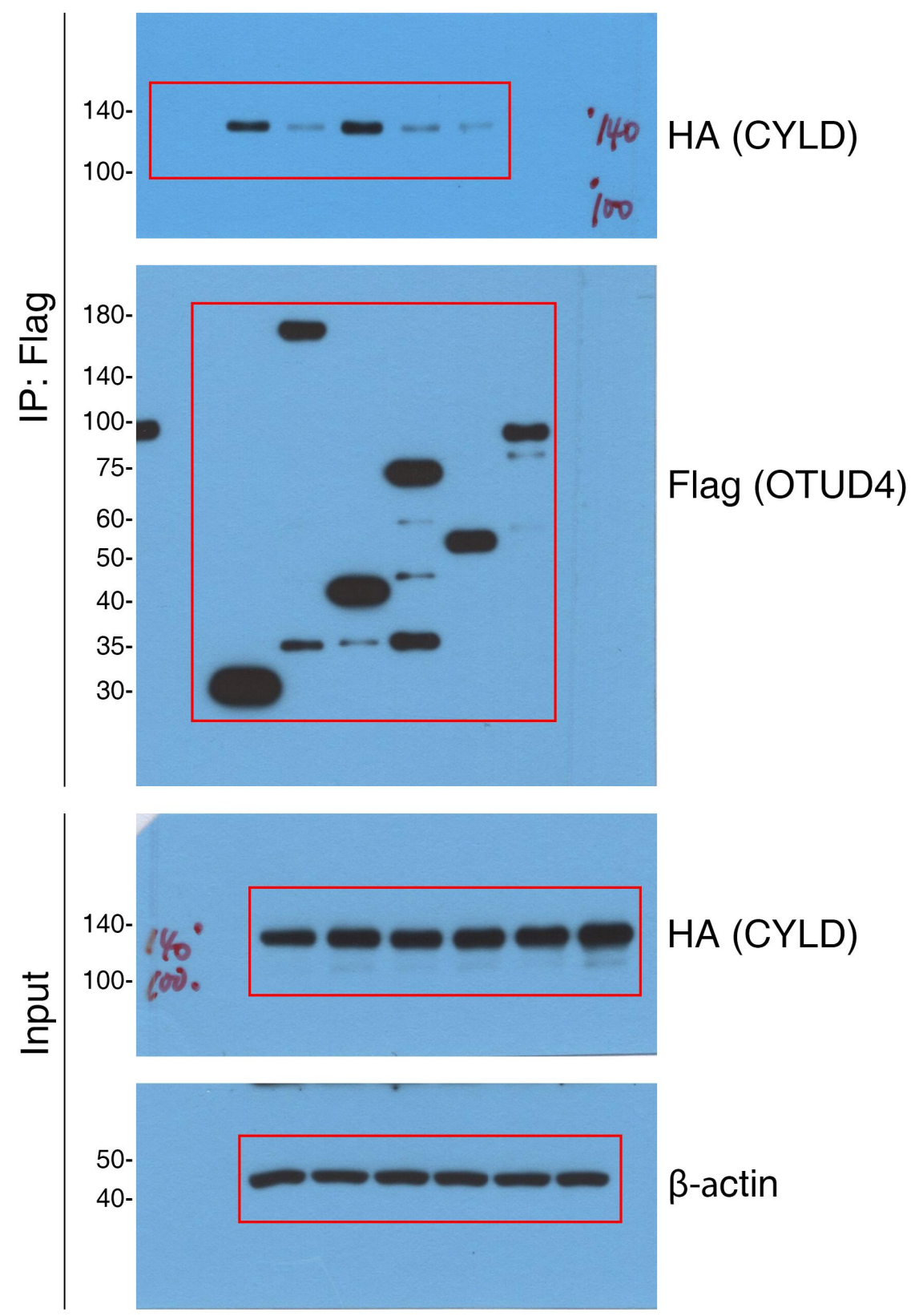

Figure 2K

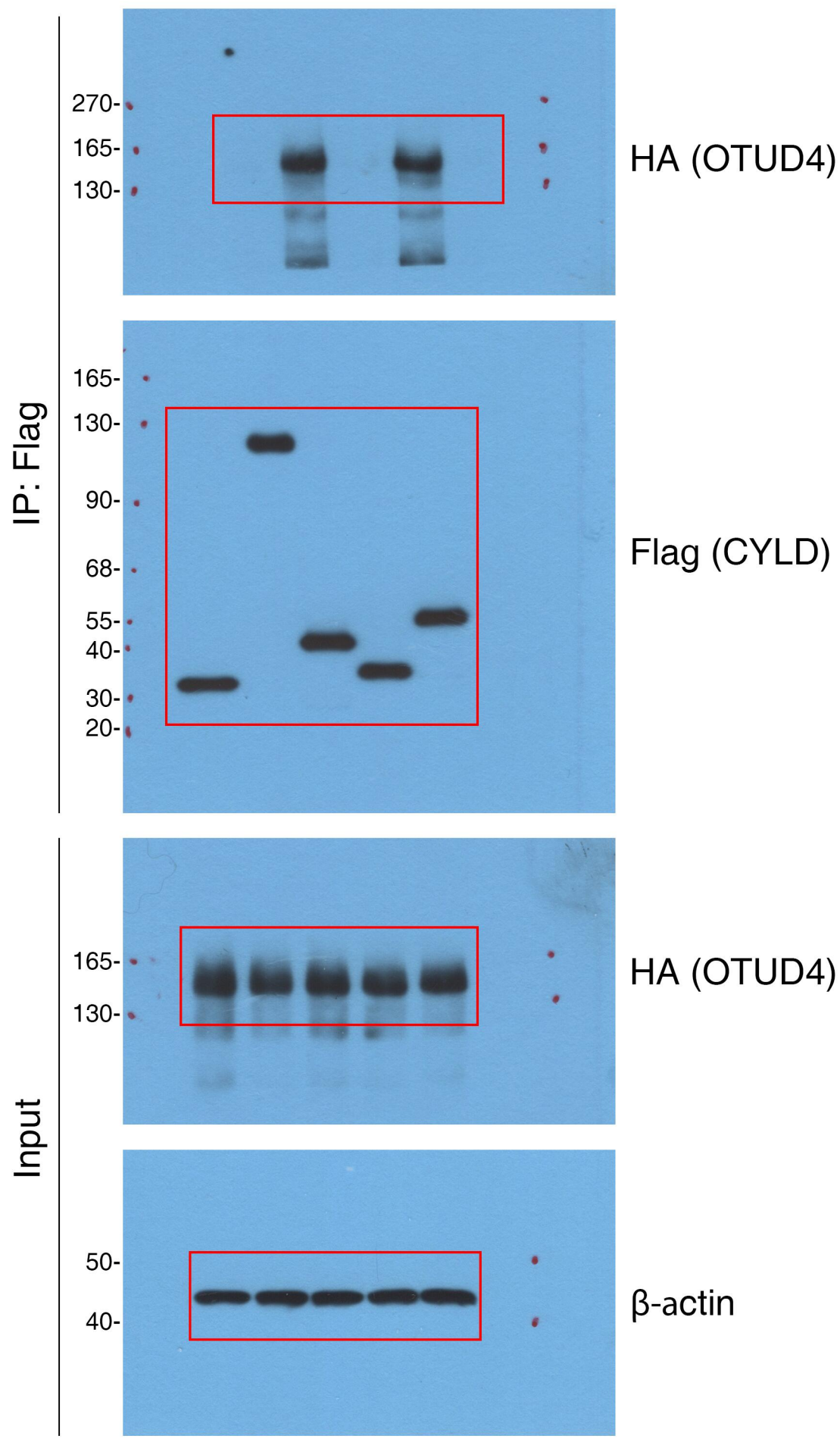

Figure 2L

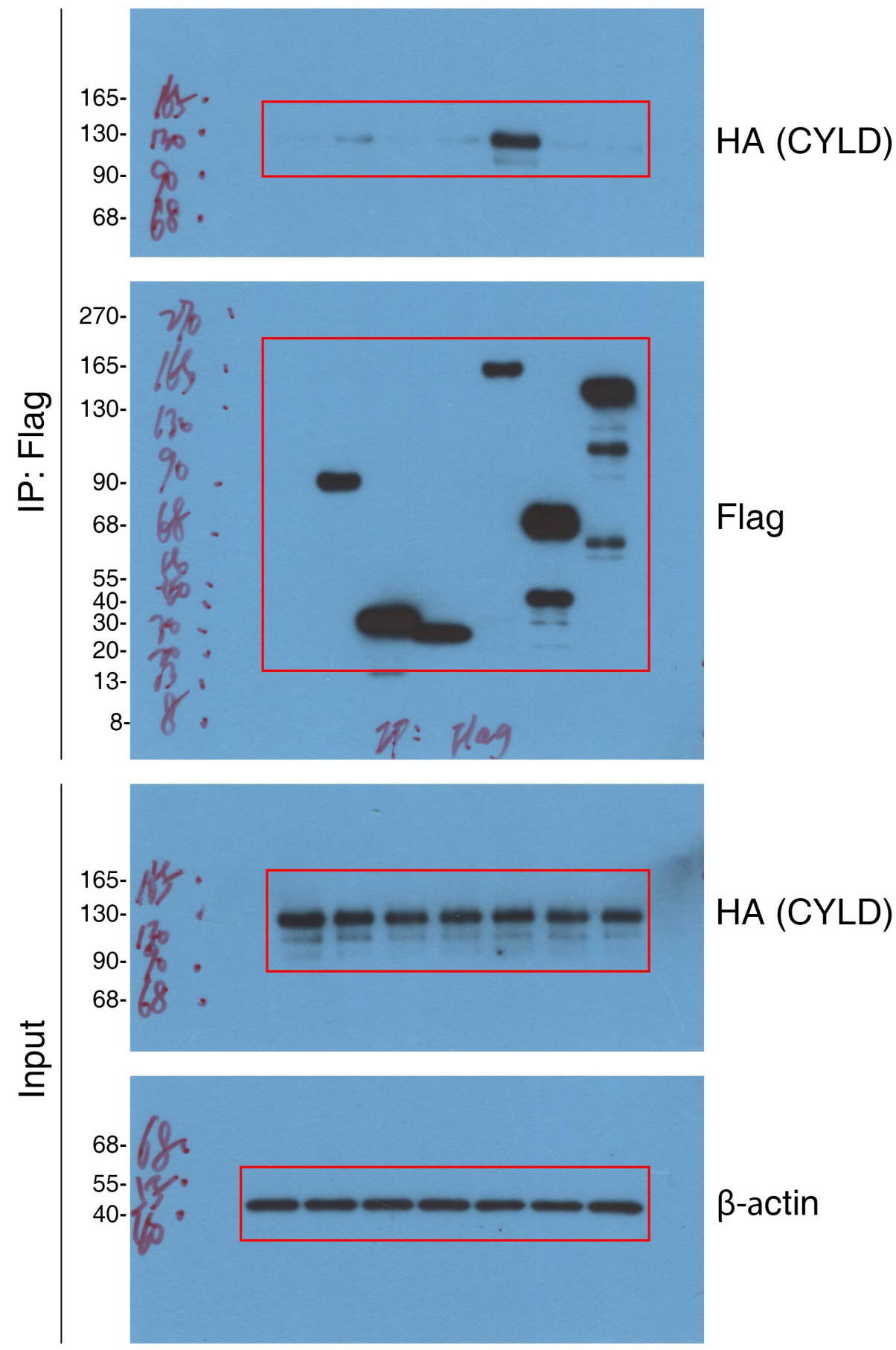

Figure 3A

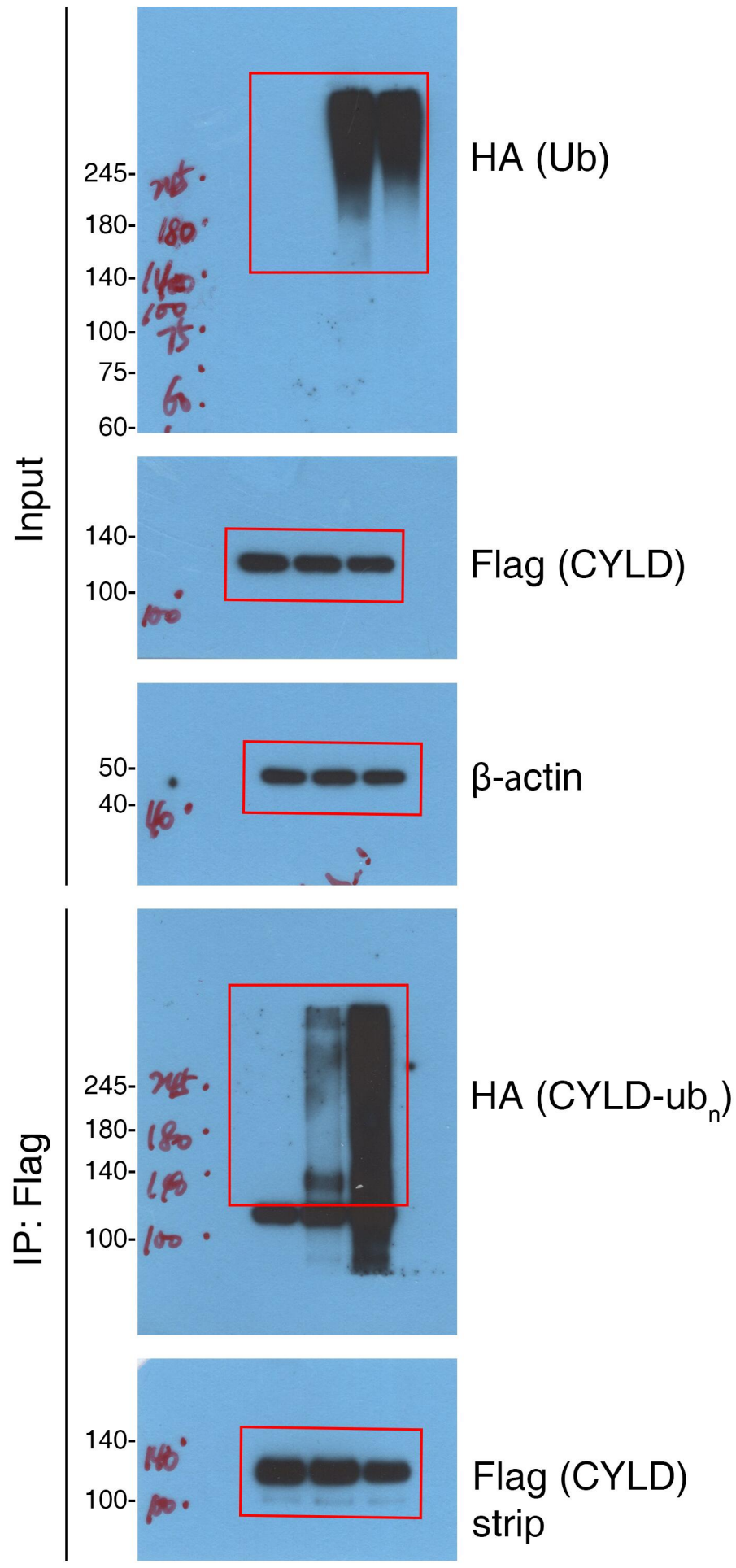

Figure 3B

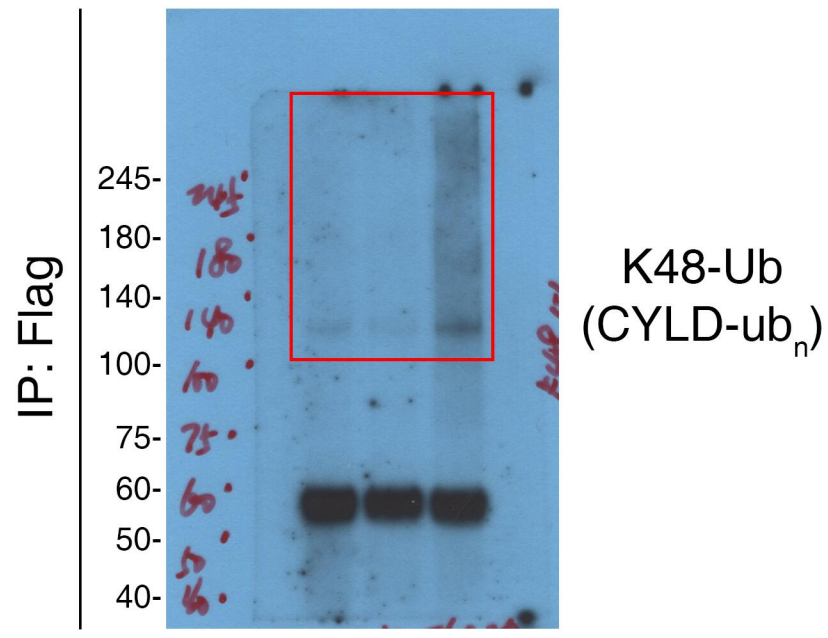

Figure 3C

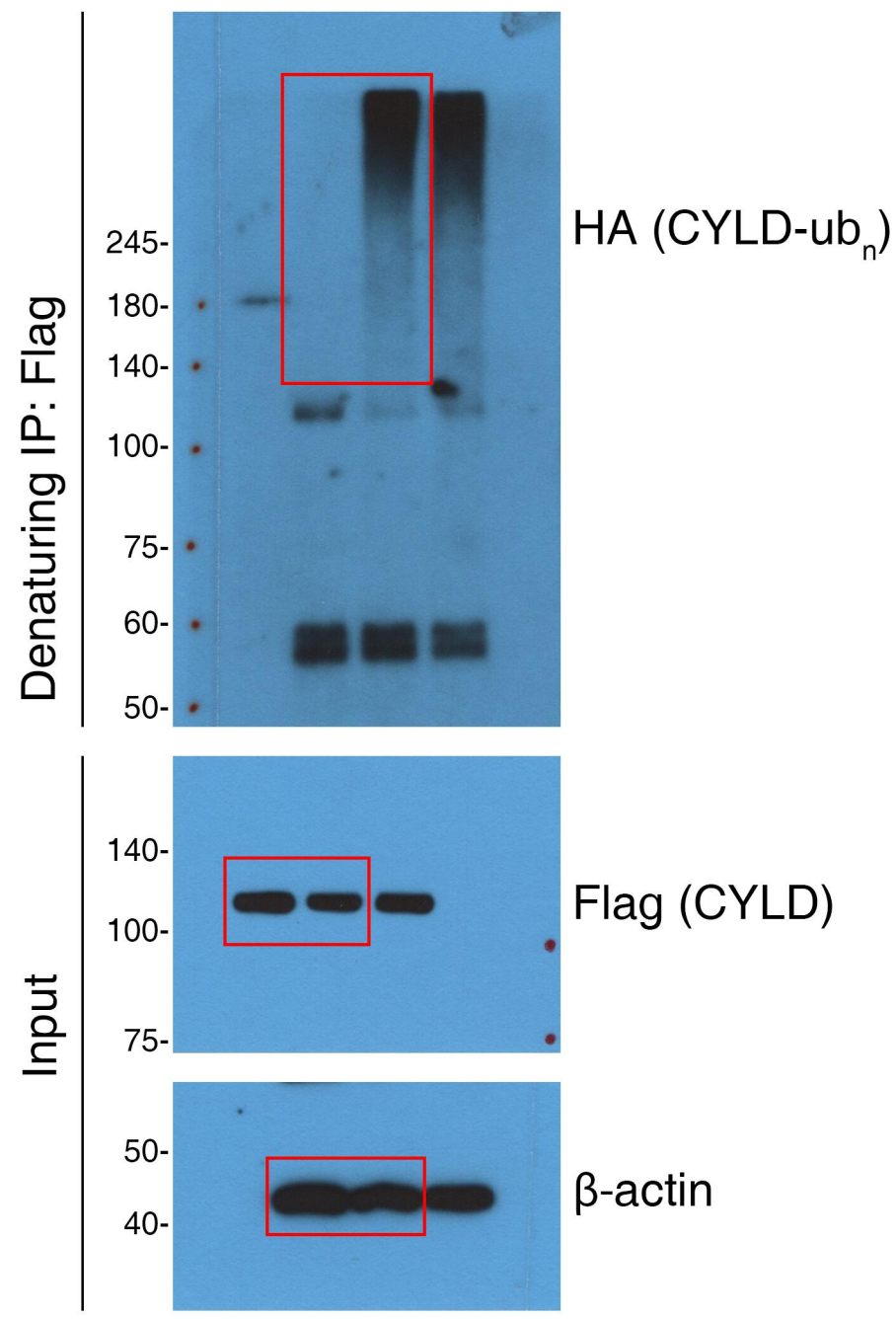

Figure 3D

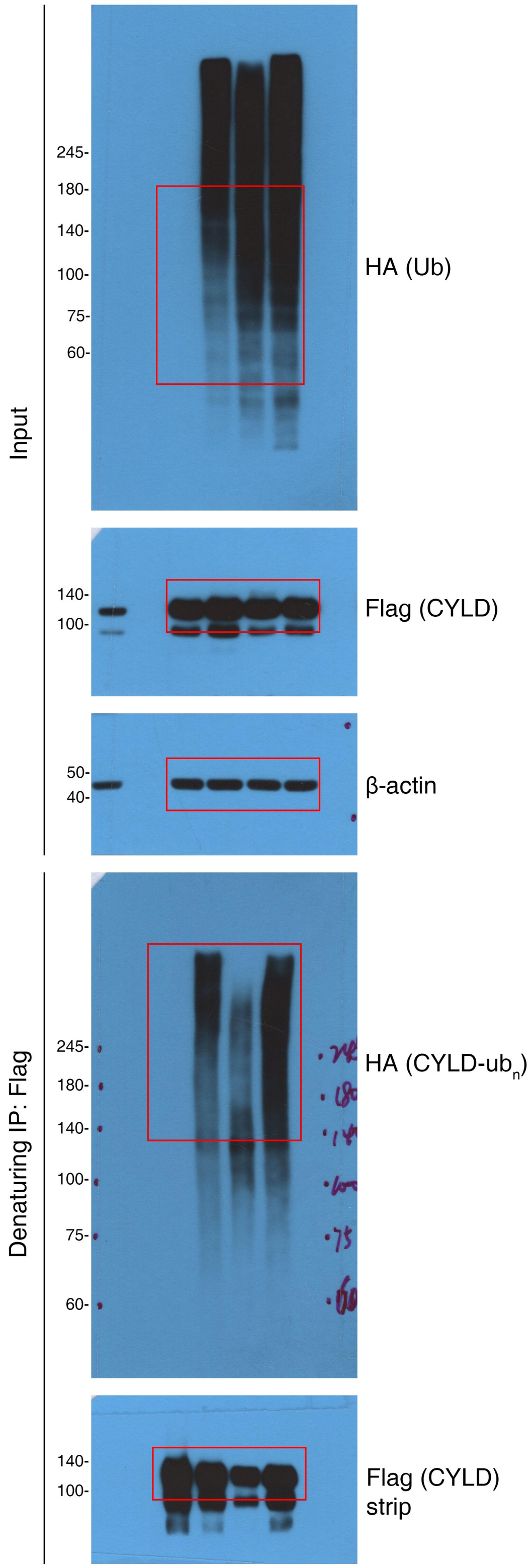

Figure 3E

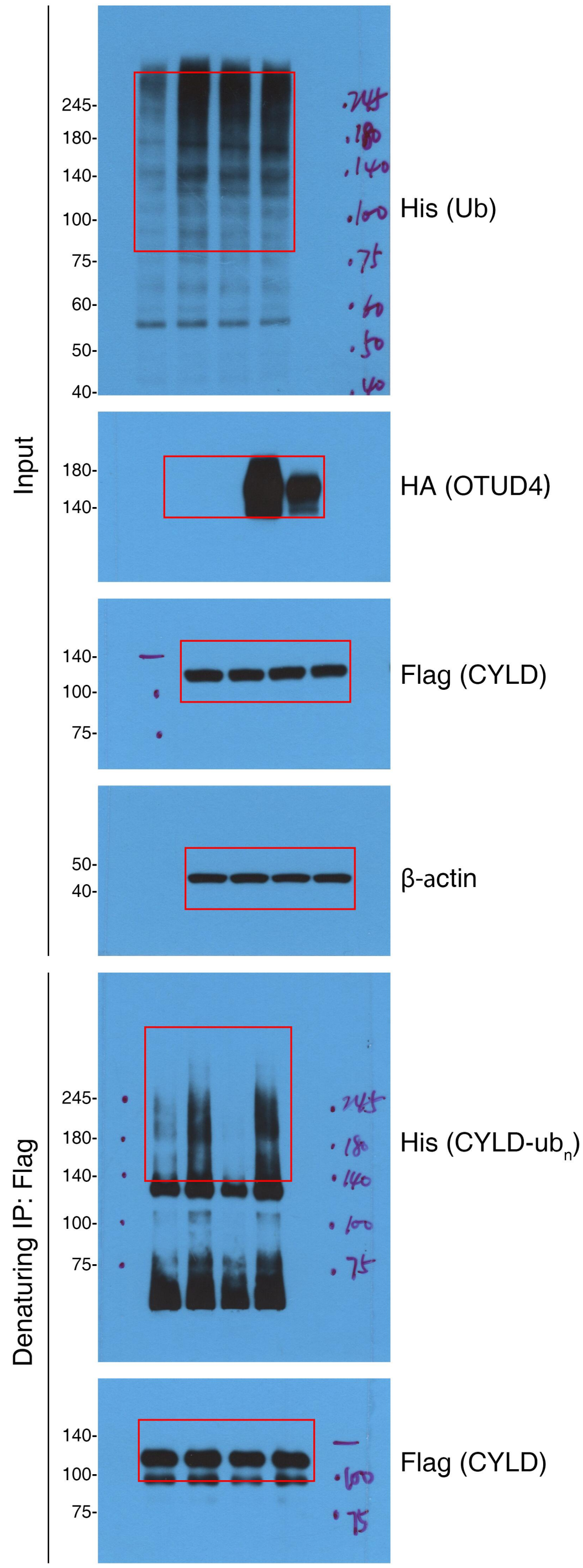

Figure 3F

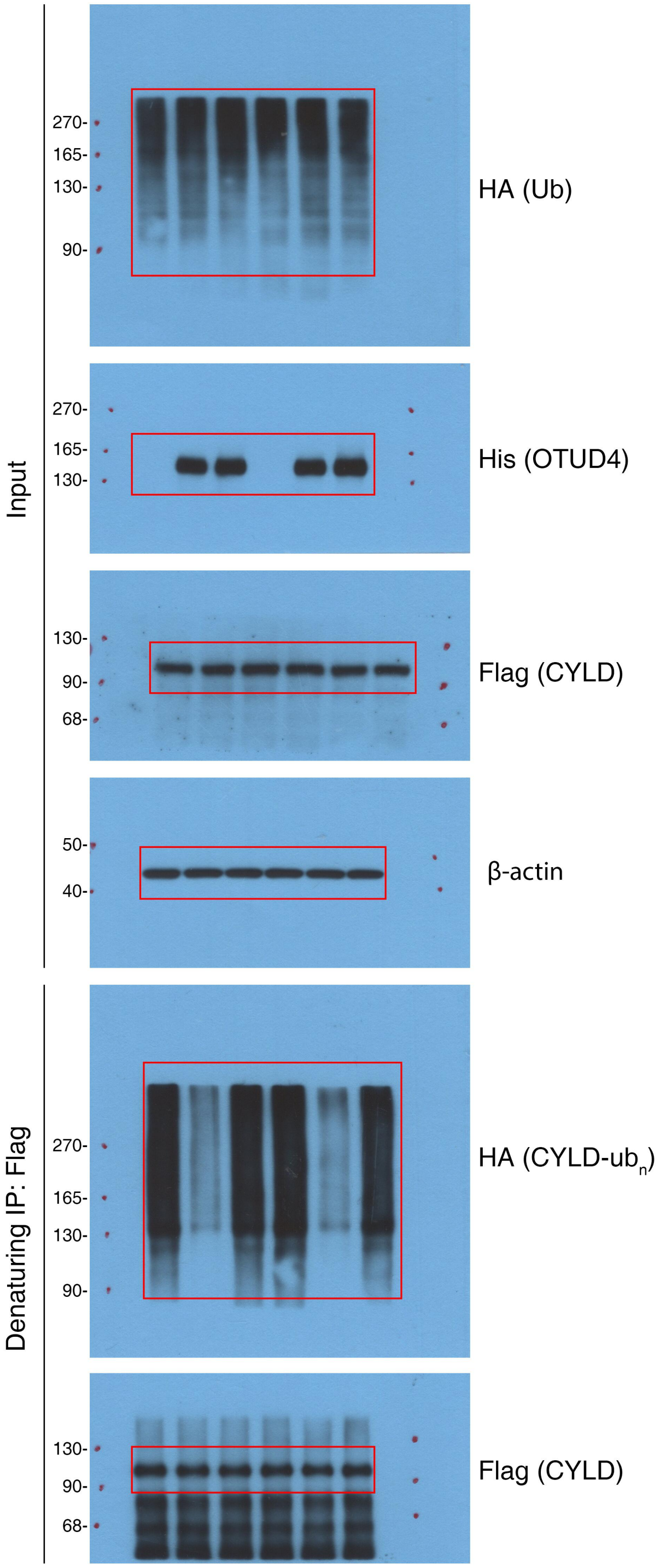

Figure 3G

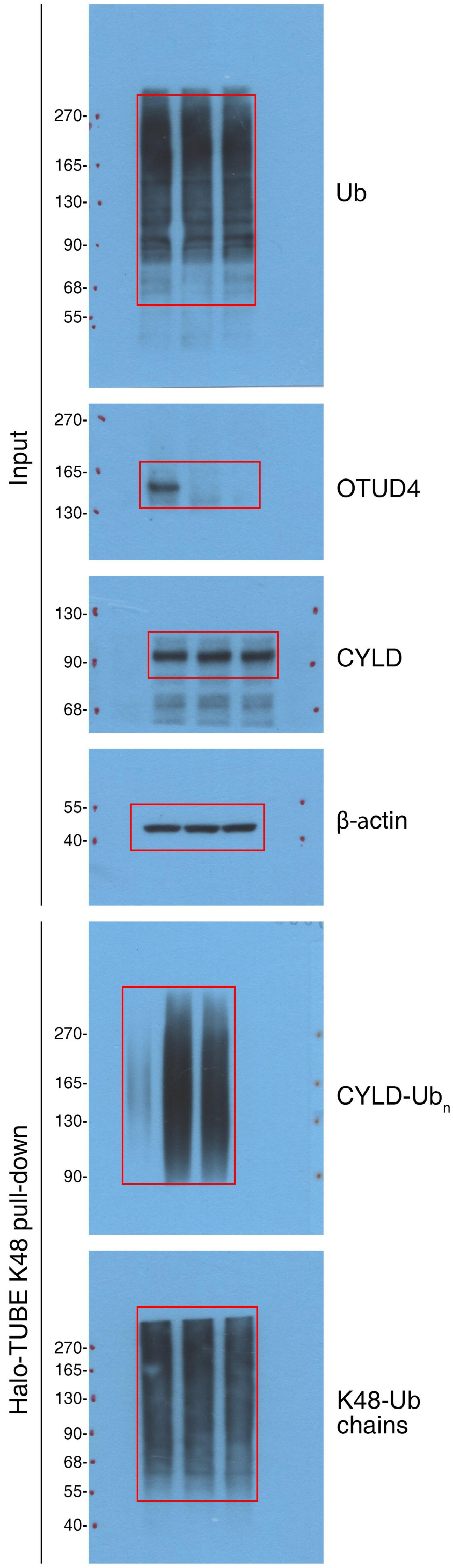

Figure 4A

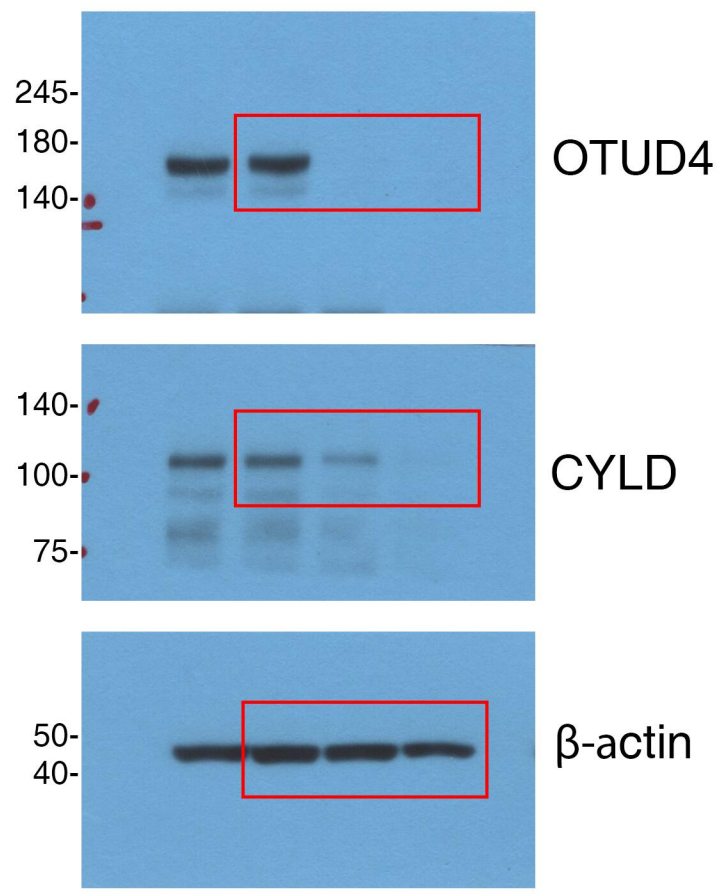

Figure 4B

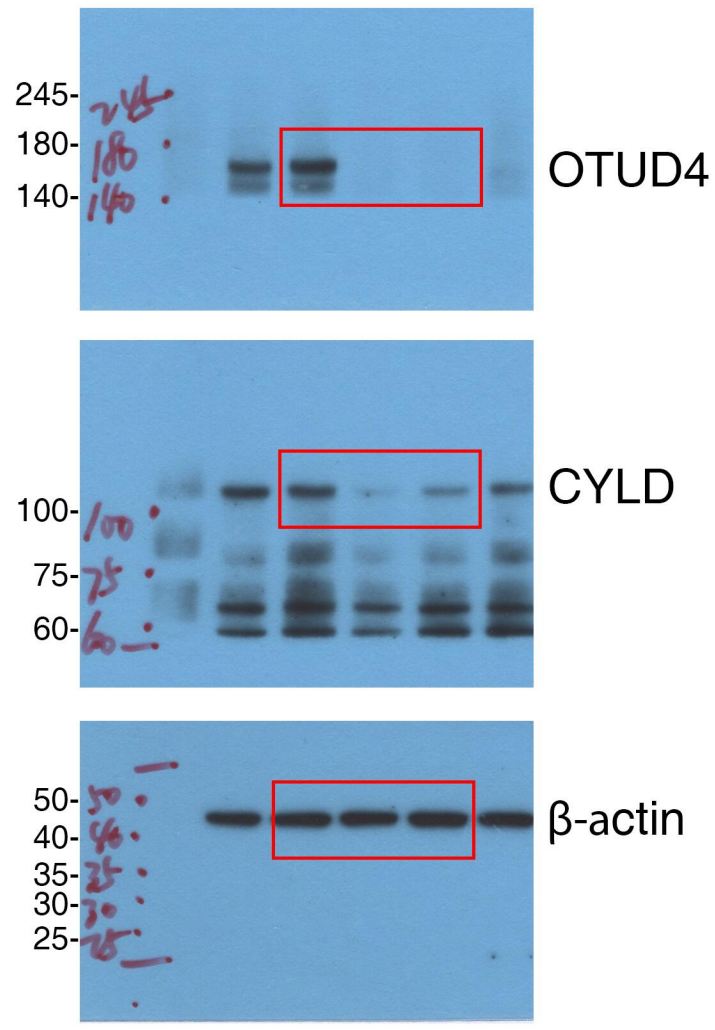

Figure 4D

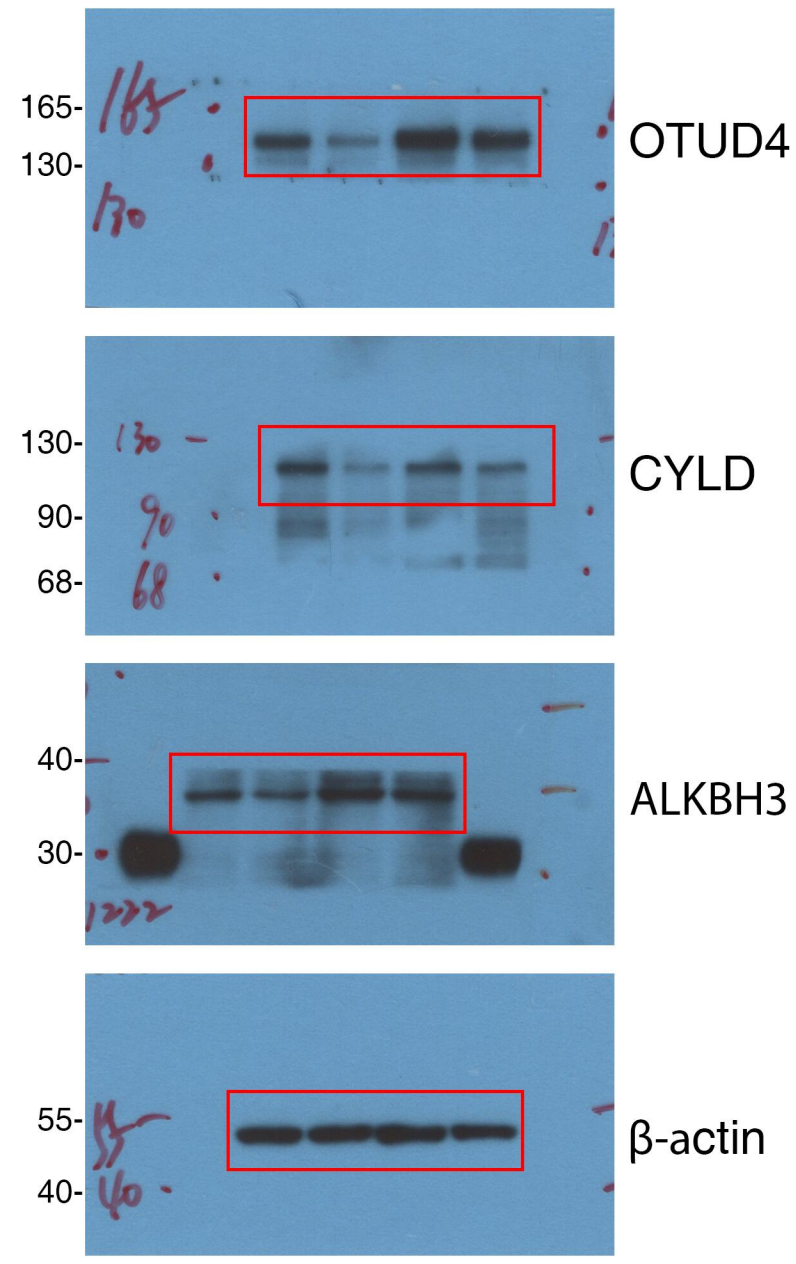

Figure 4E

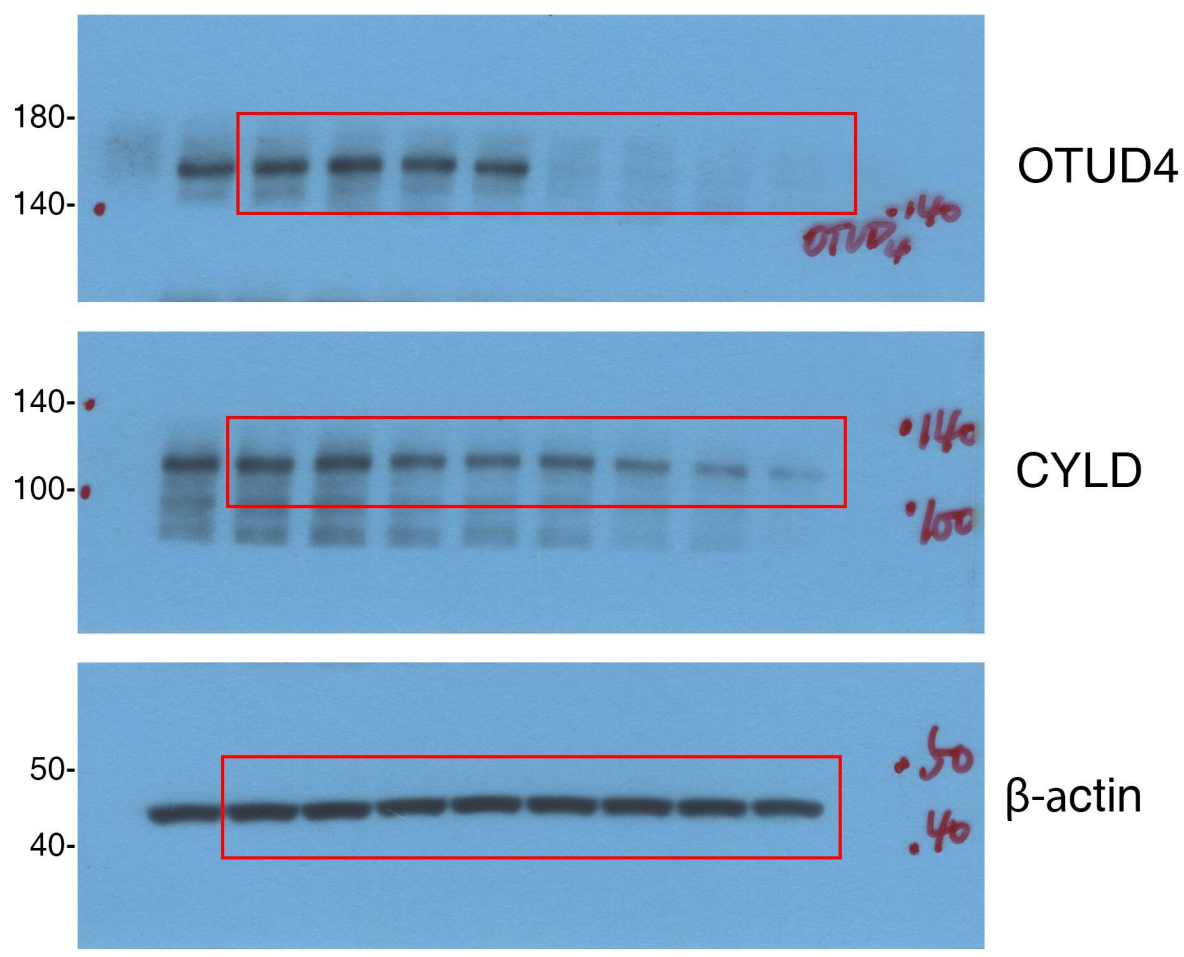

Figure 4G

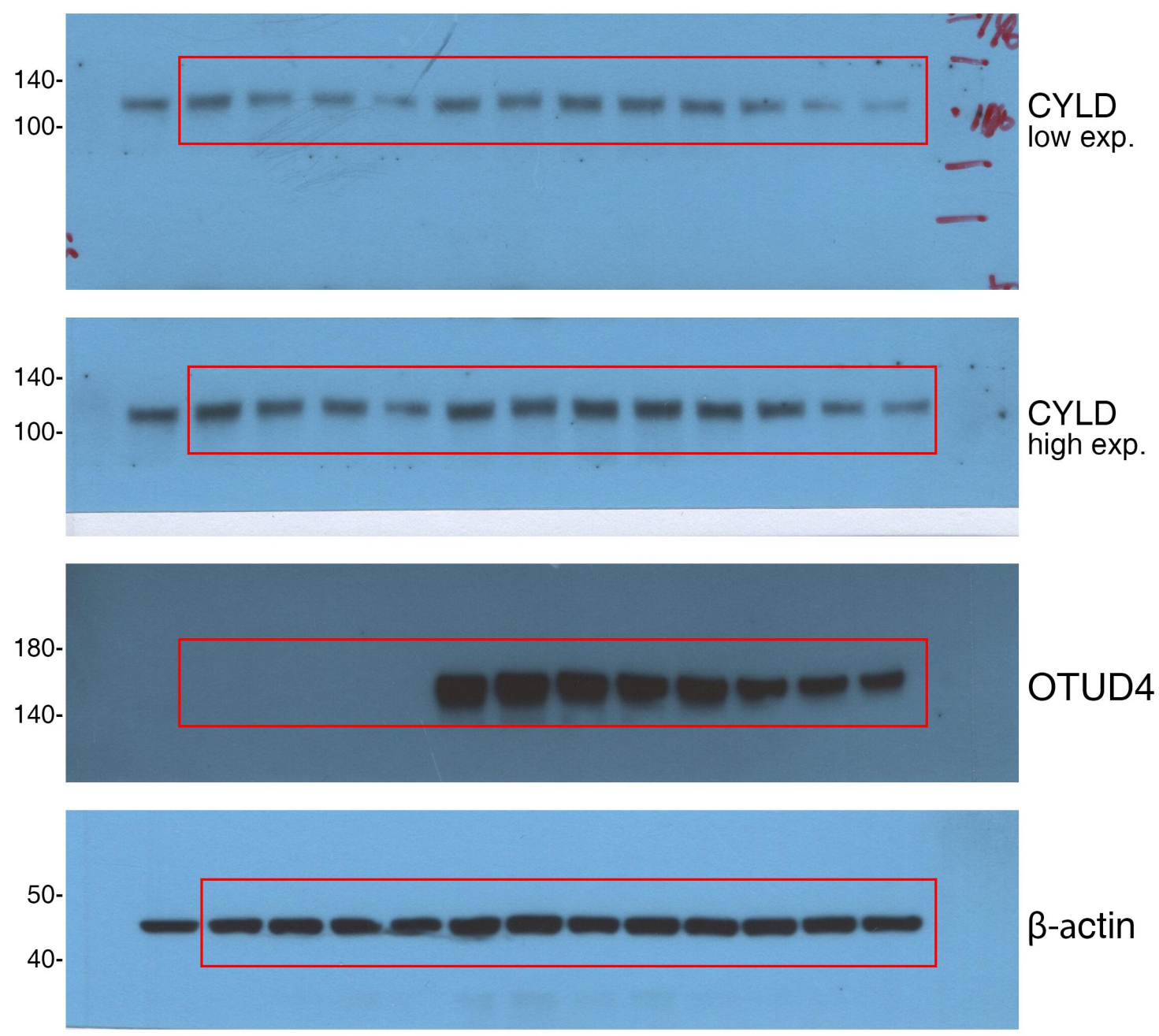

Figure 4I

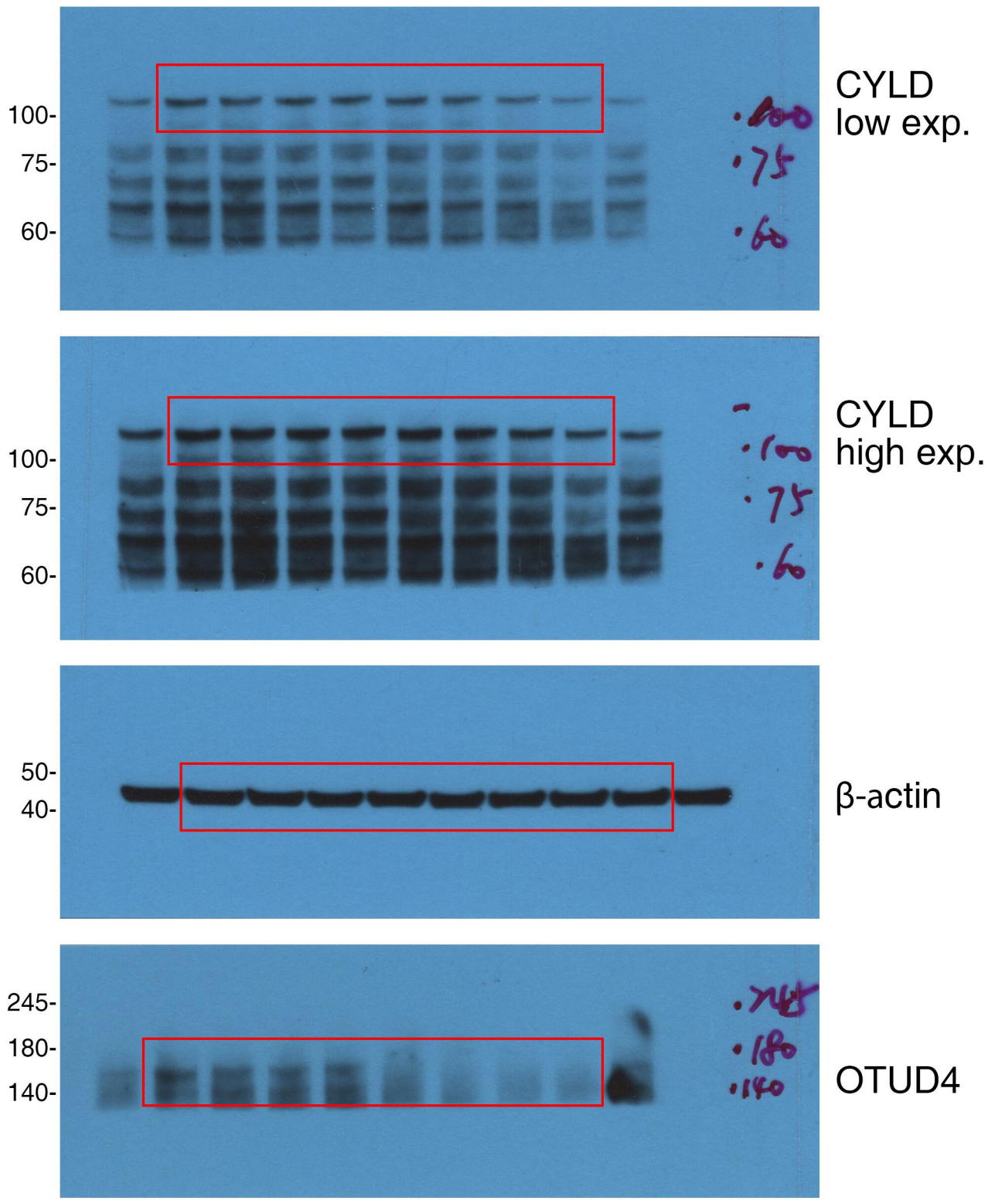

Figure 4K

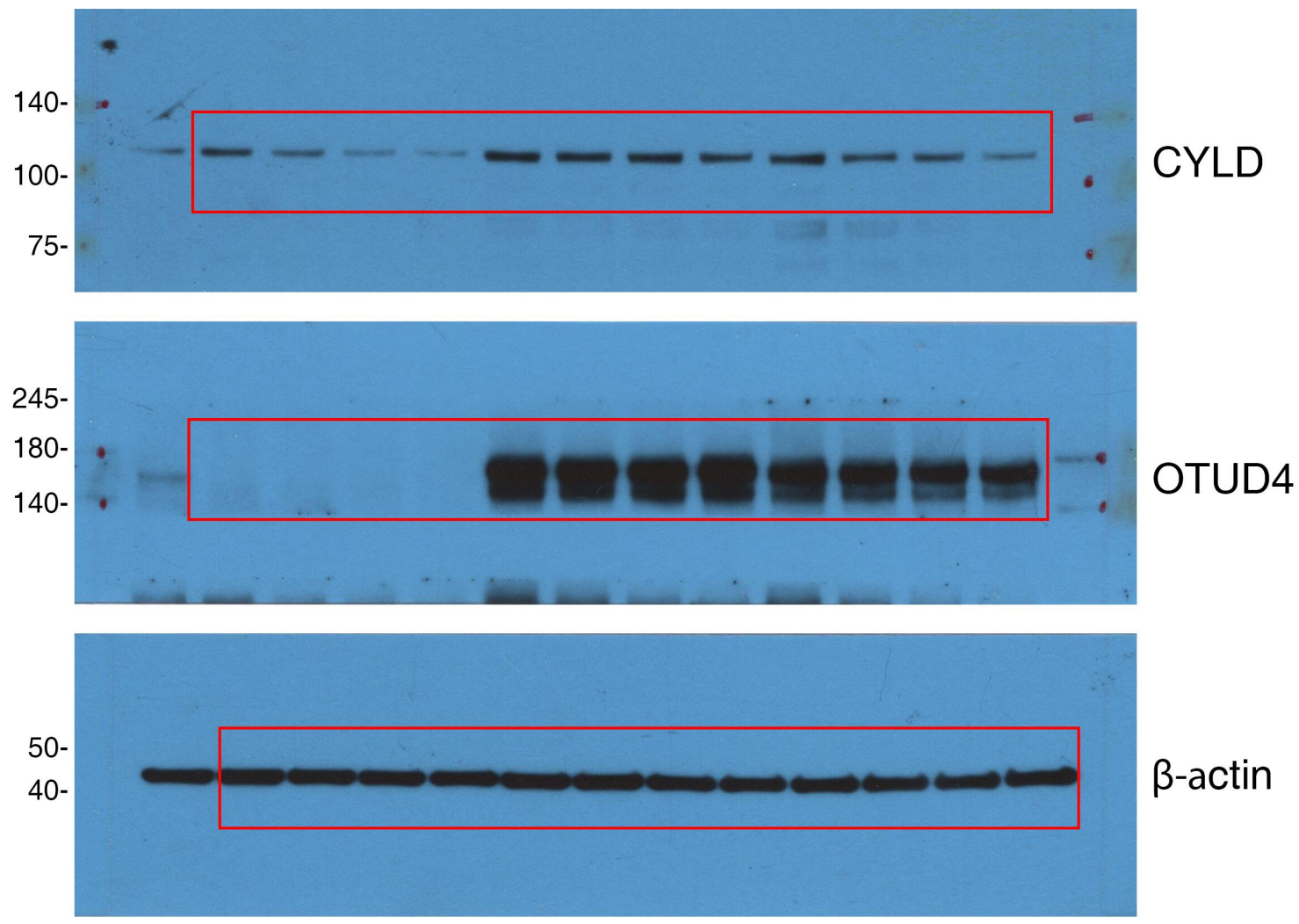

Figure 4M

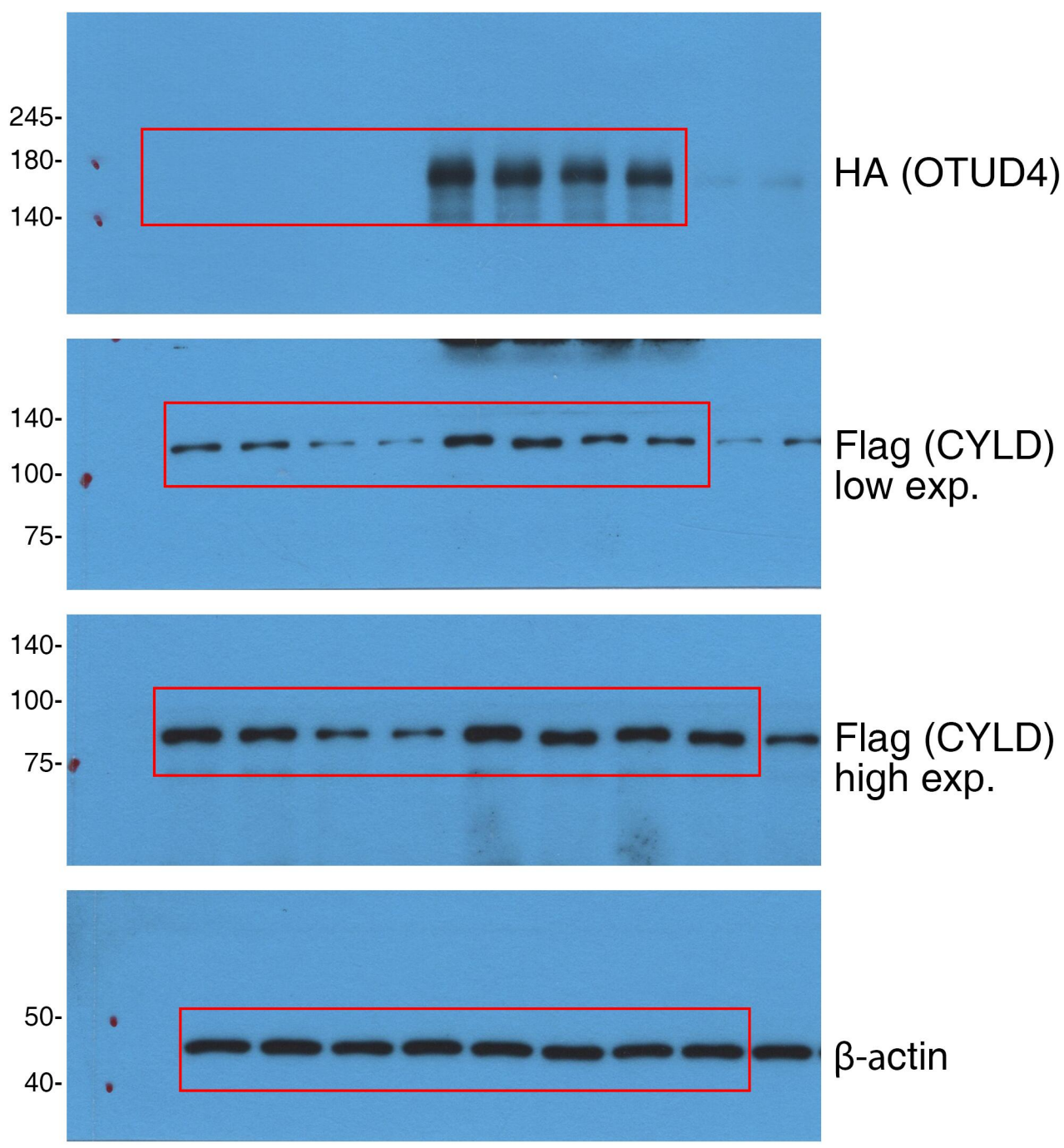

Figure 5A

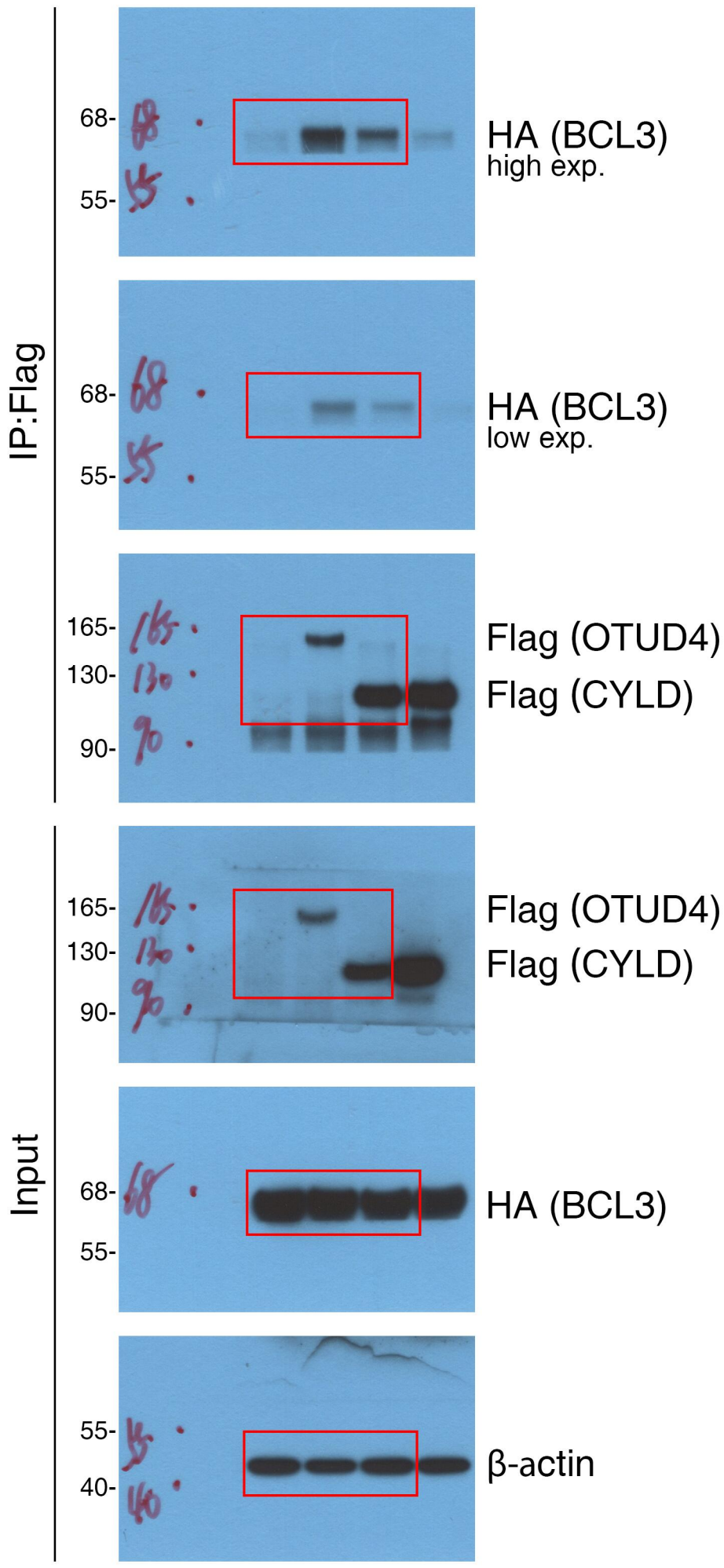

Figure 5B

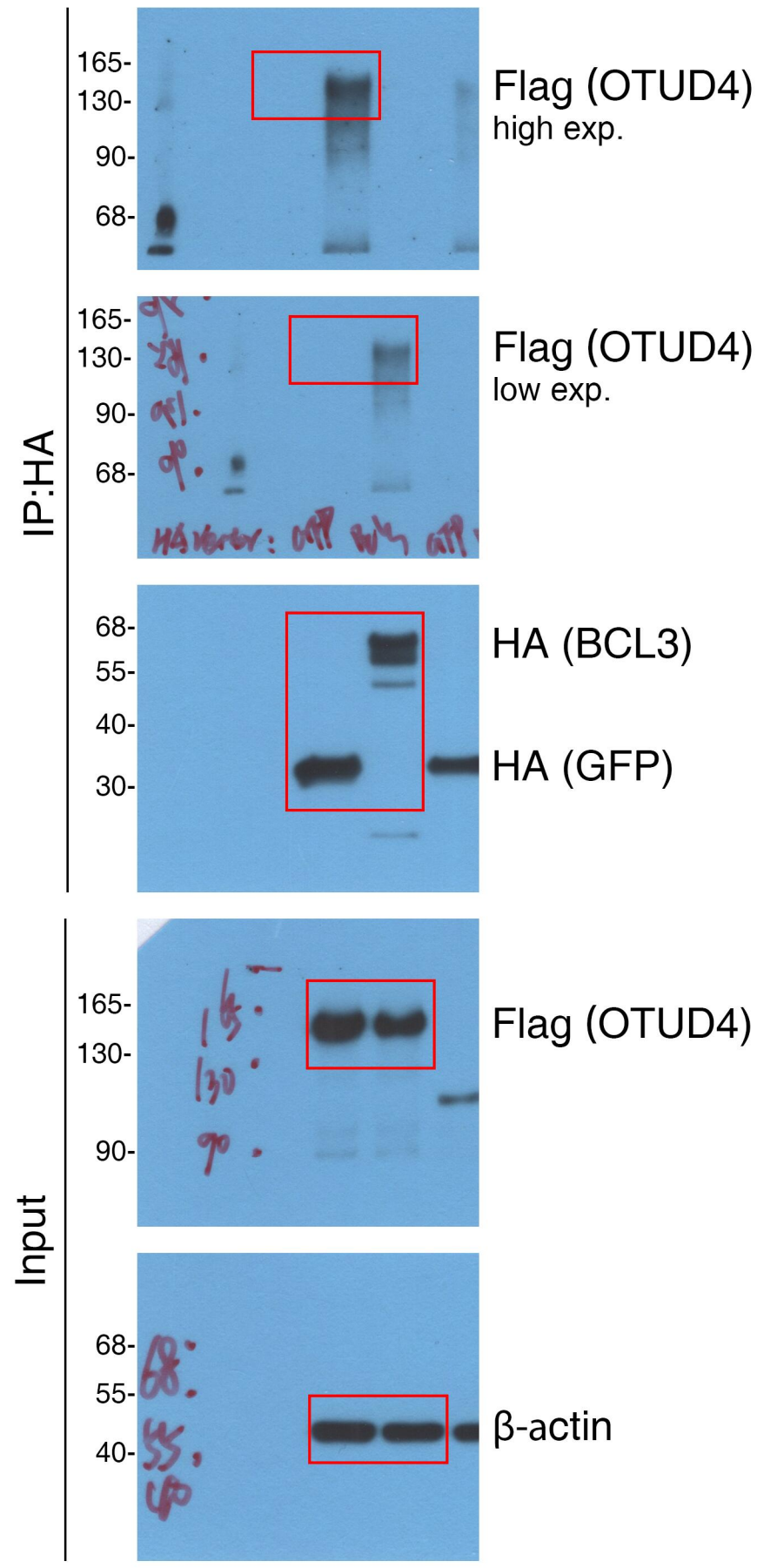

Figure 5C

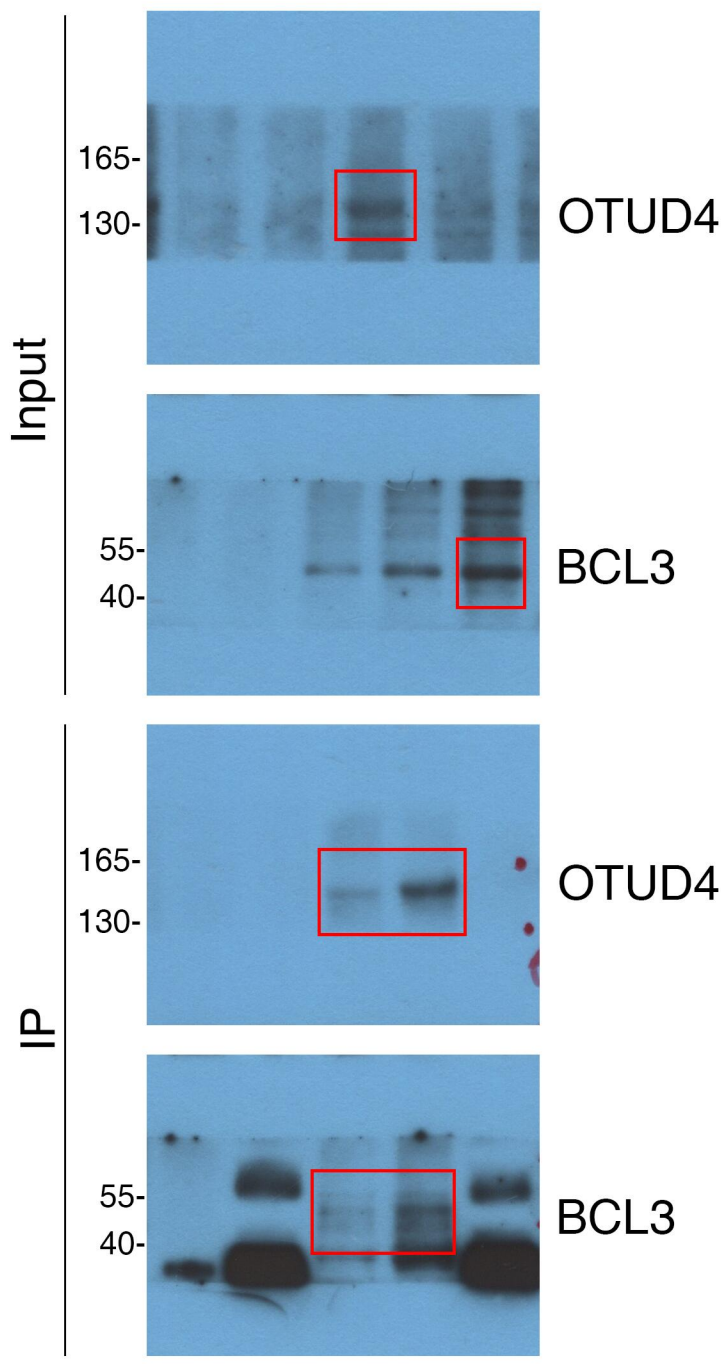

Figure 5D

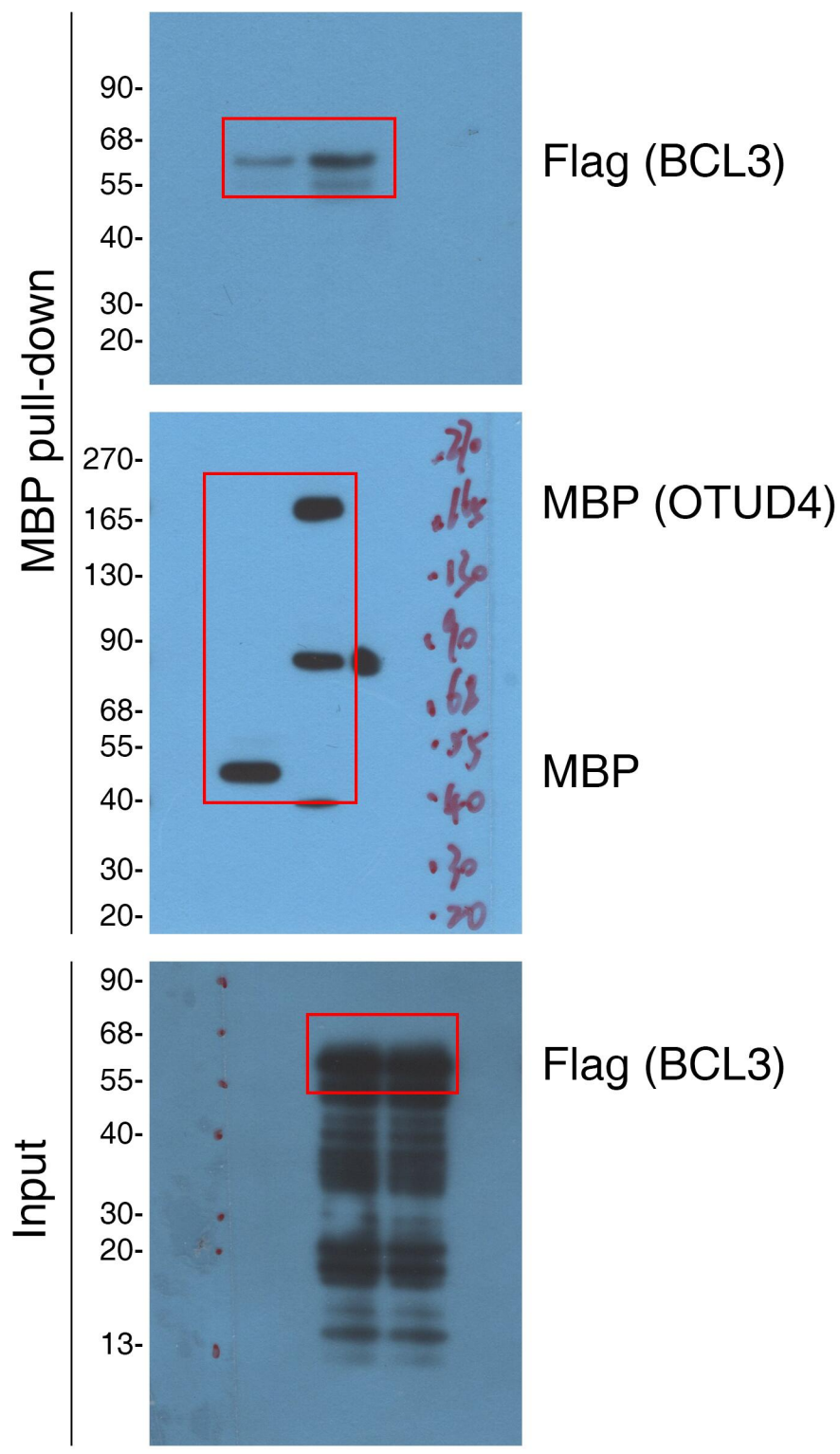

Figure 5F

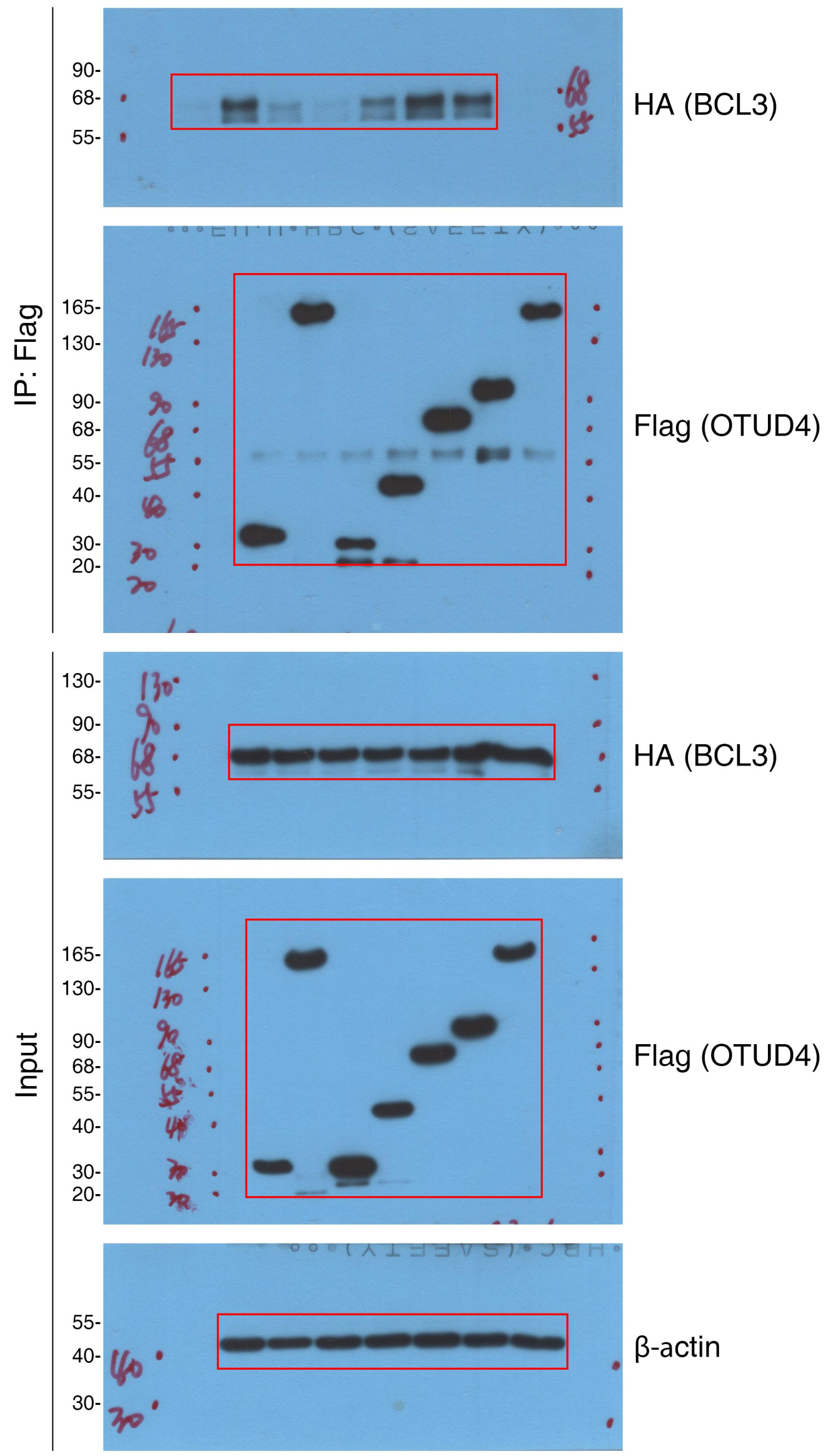

Figure 5H

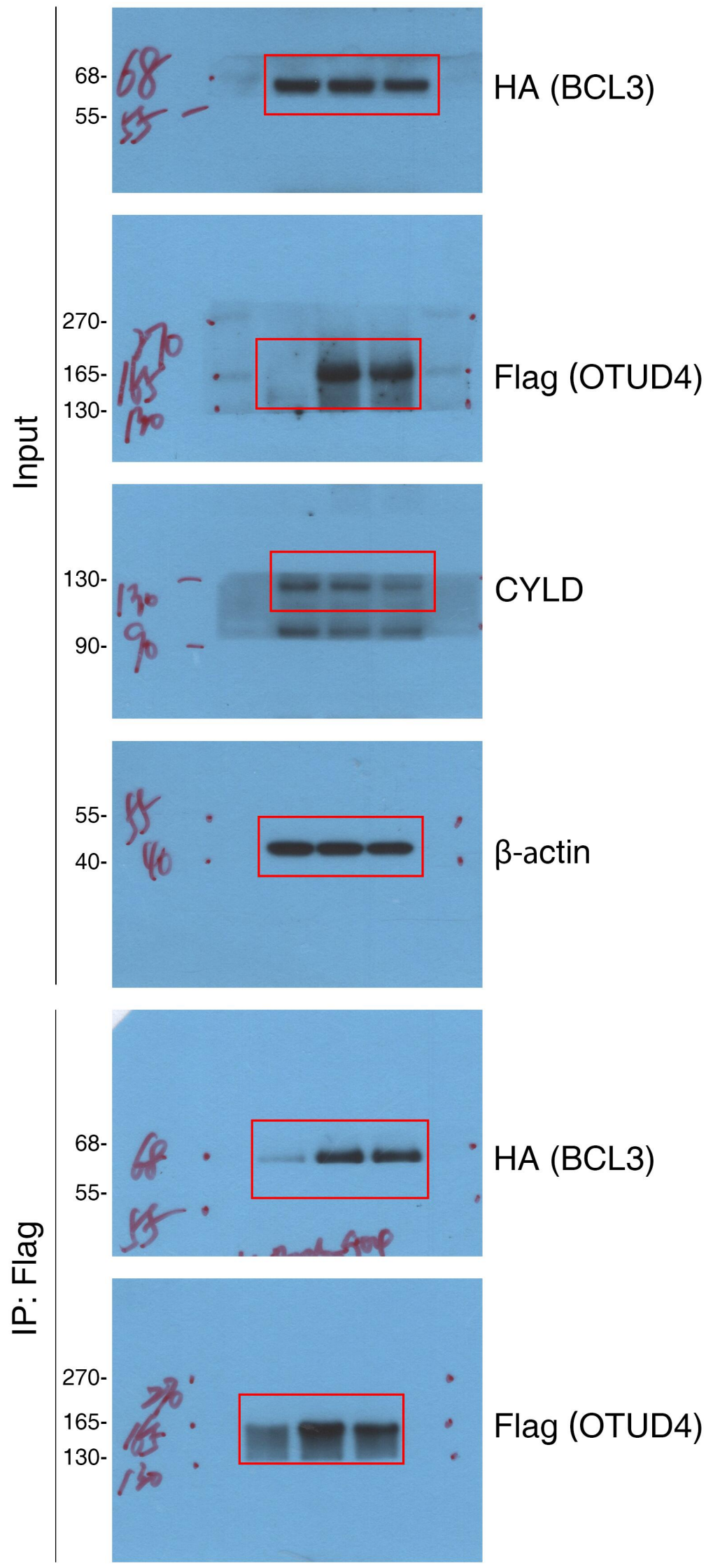

Figure 6A

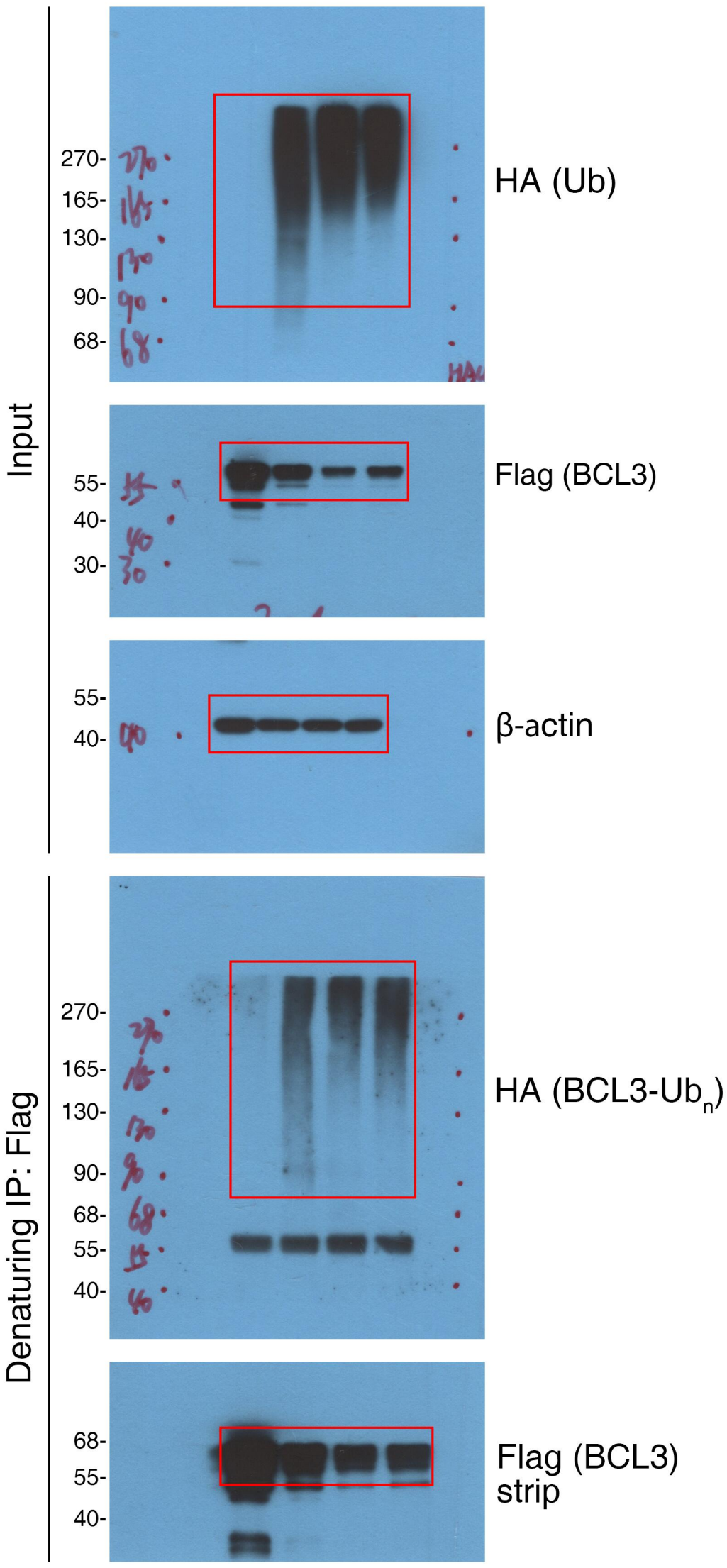

Figure 6B

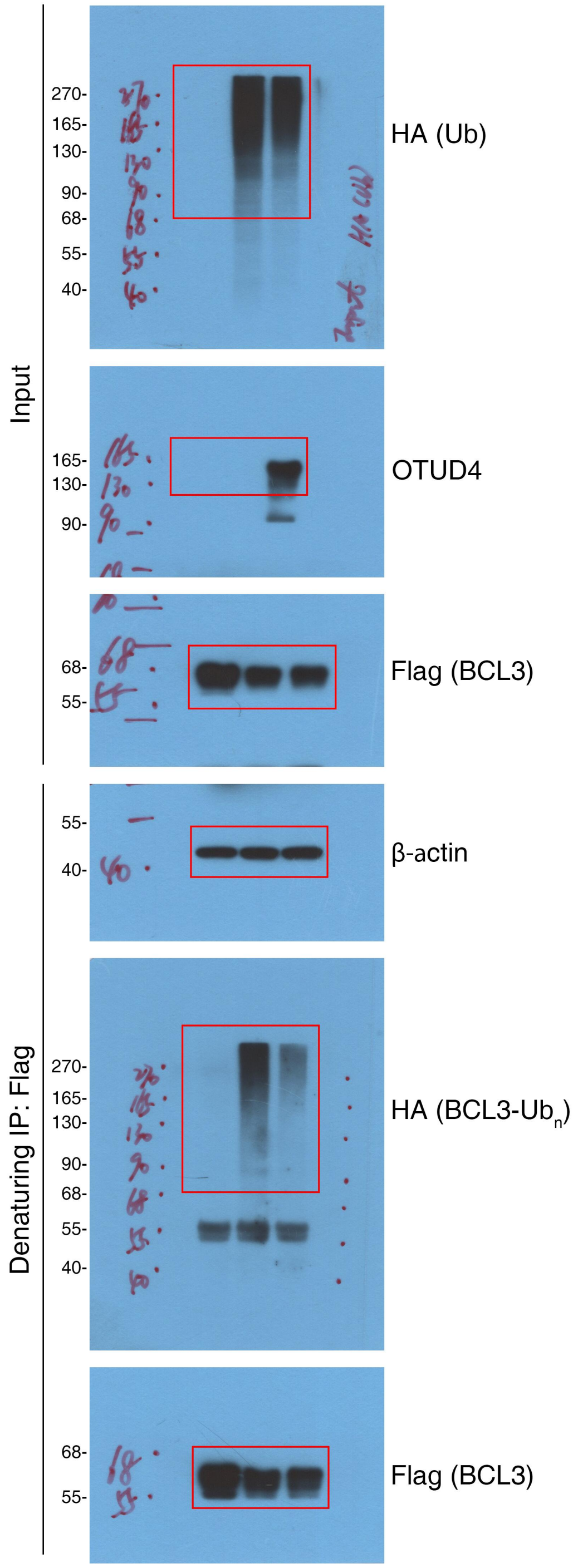

Figure 6C

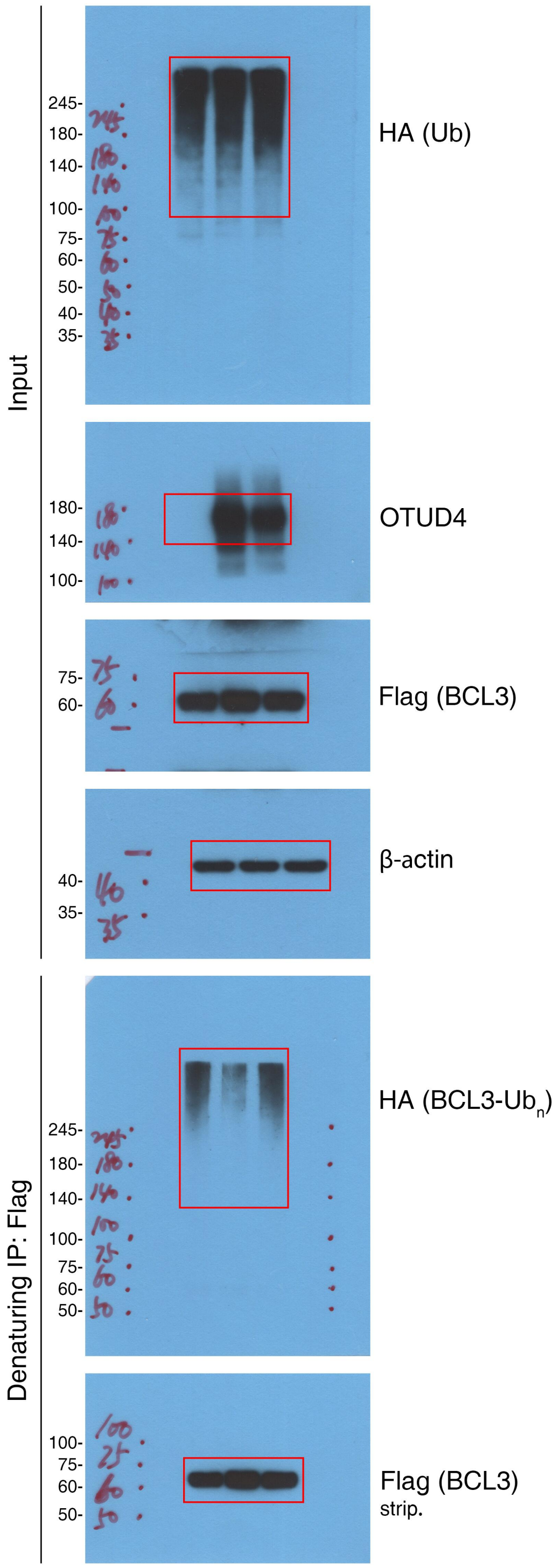

Figure 6D

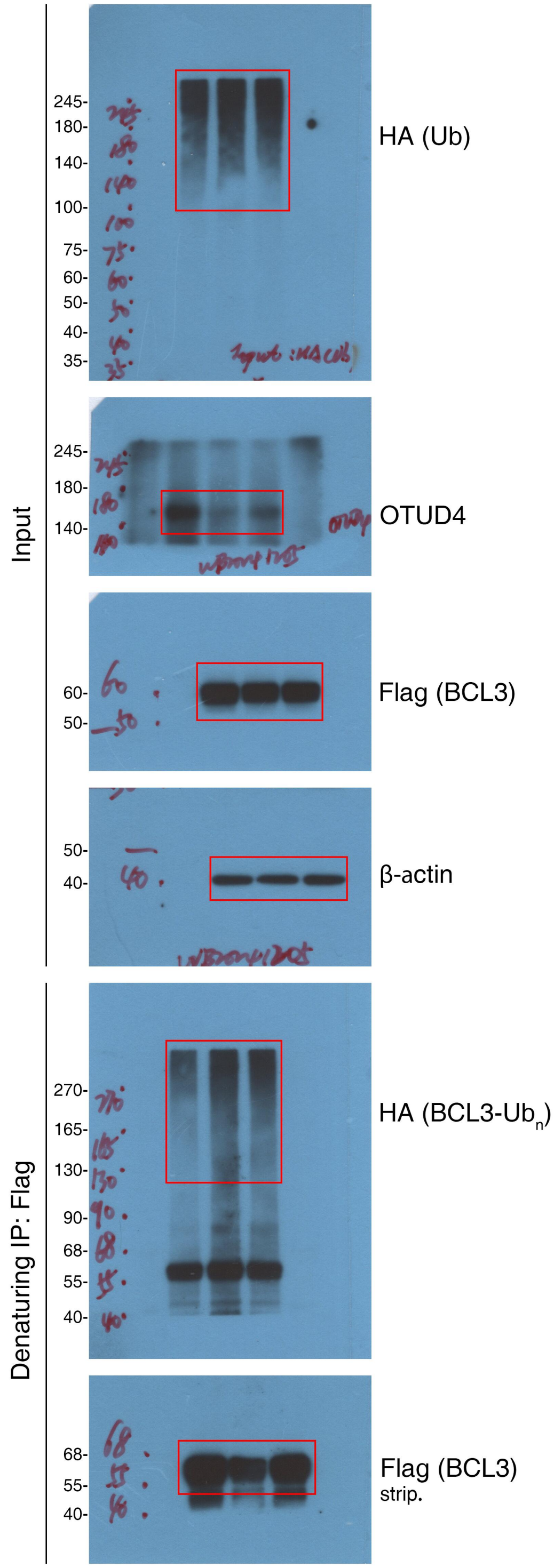

Figure 6E

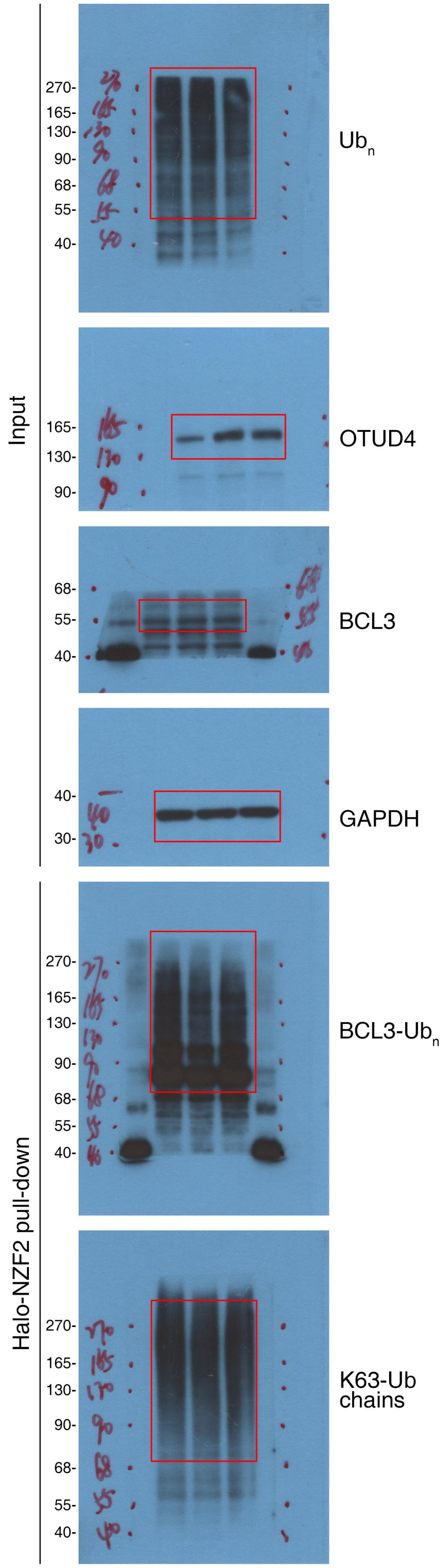

Figure 6F

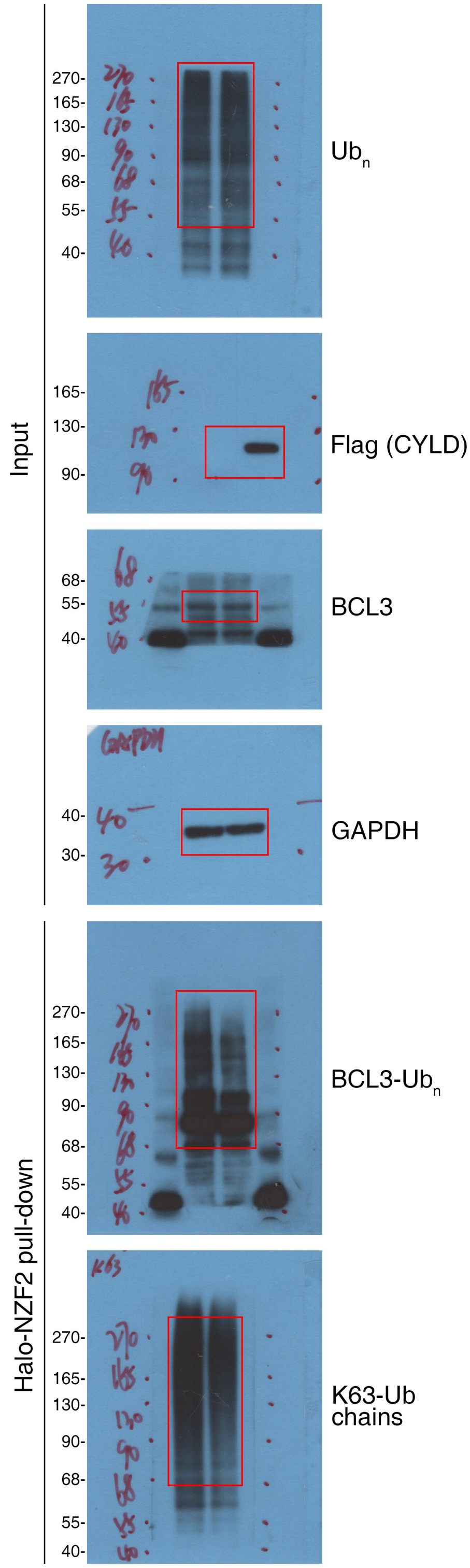

Figure 6G

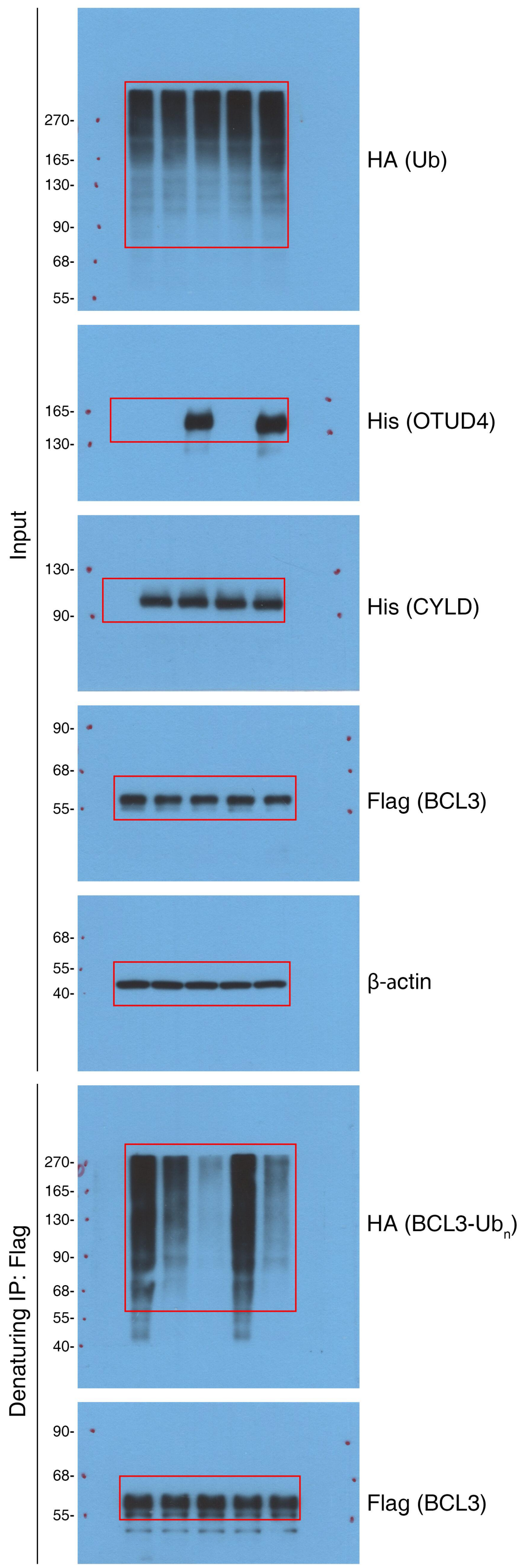

Figure 6H

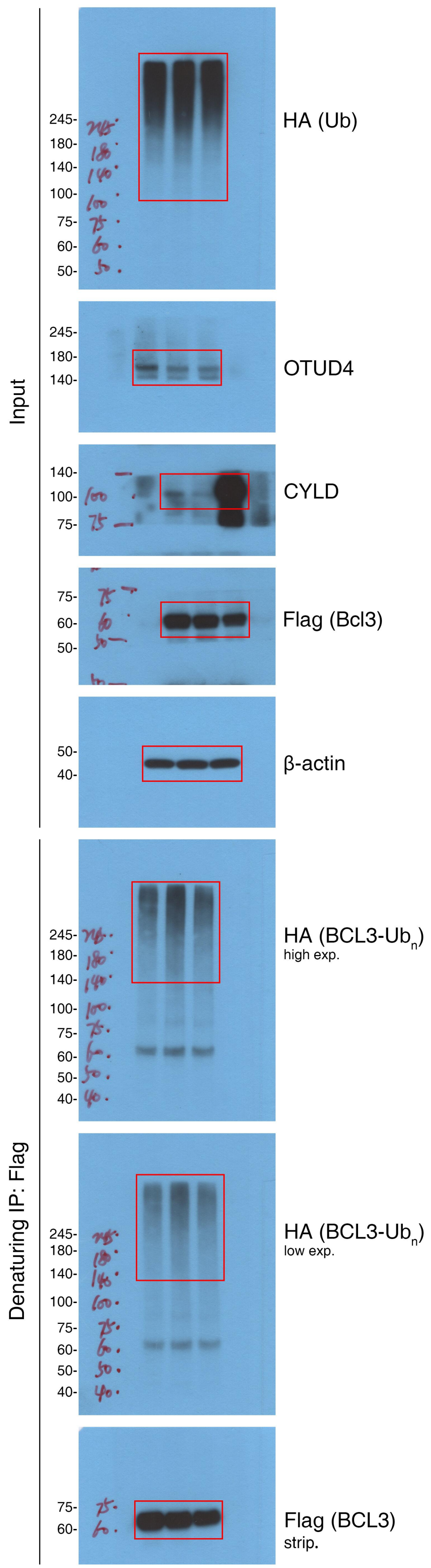

50-

Figure 7E

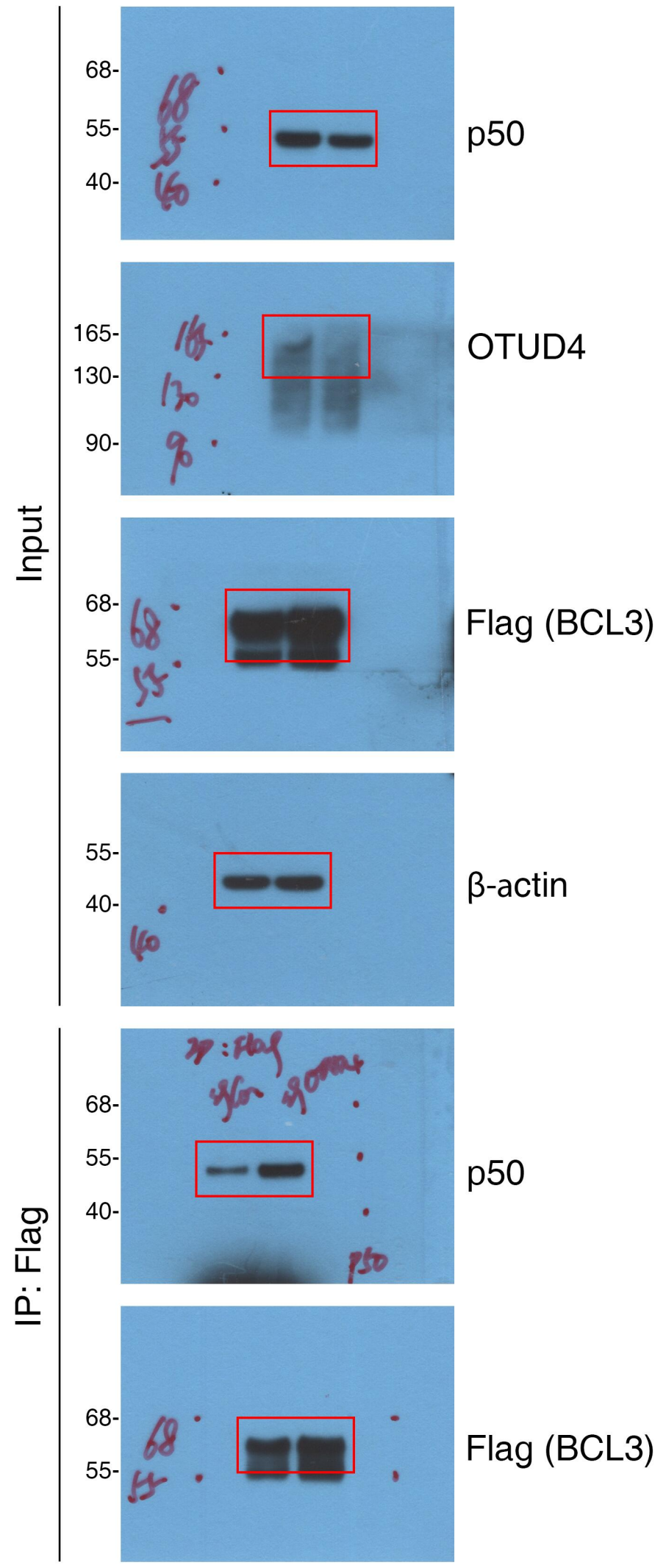

Figure 7H

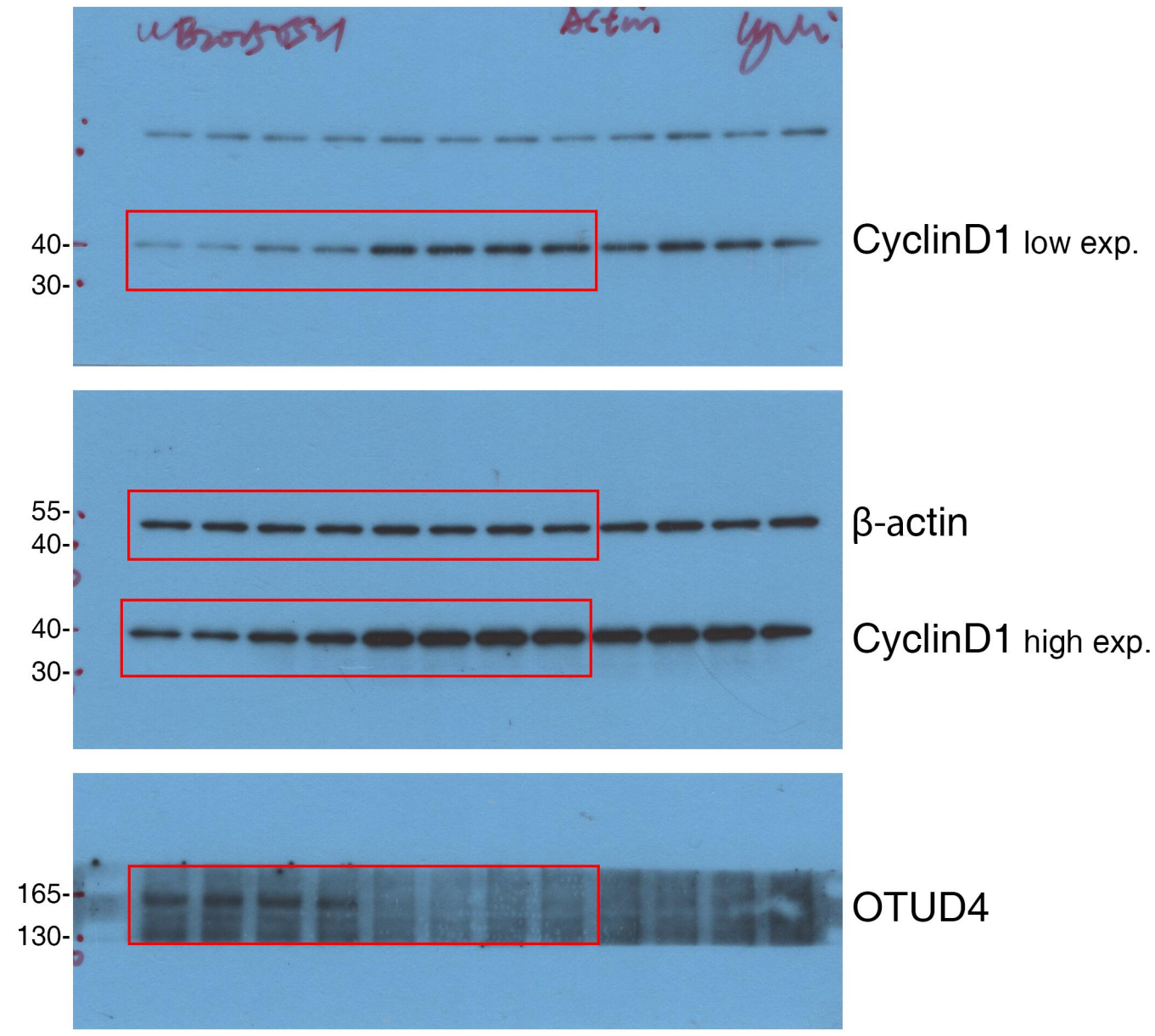

Figure 8B

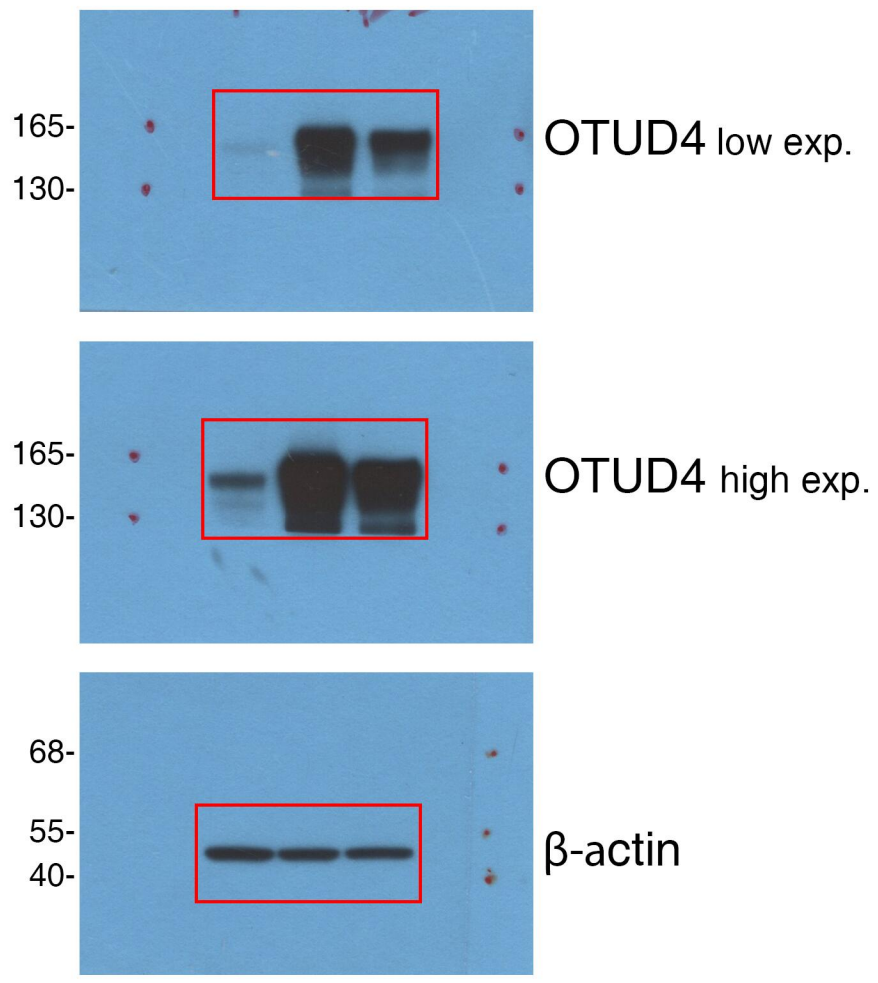

Figure 8D

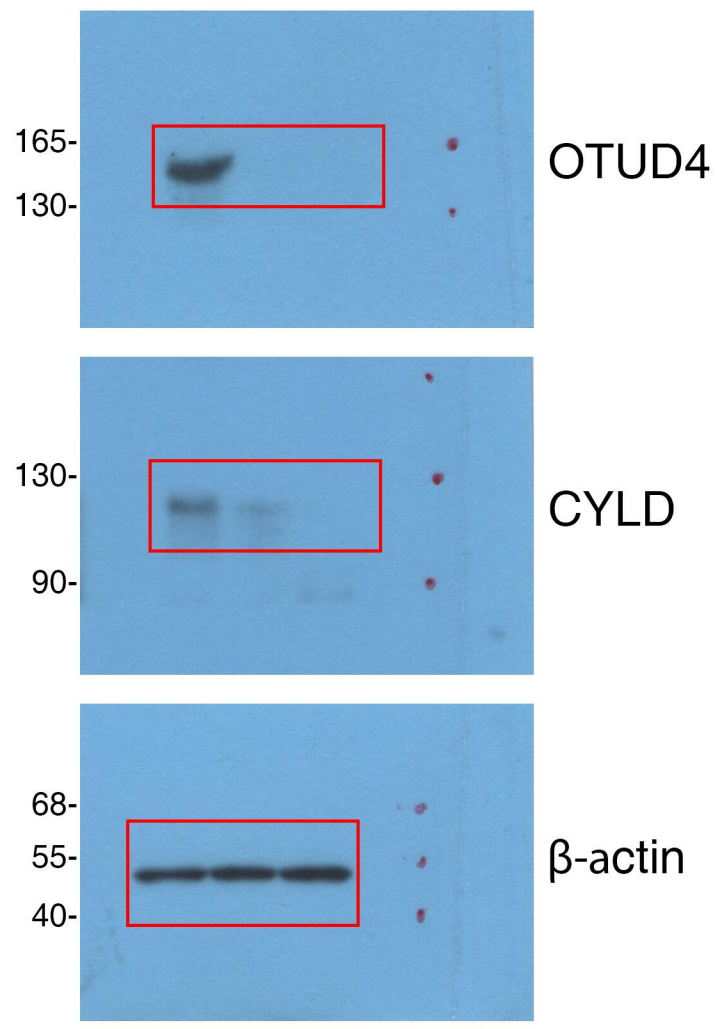

Figure 8F

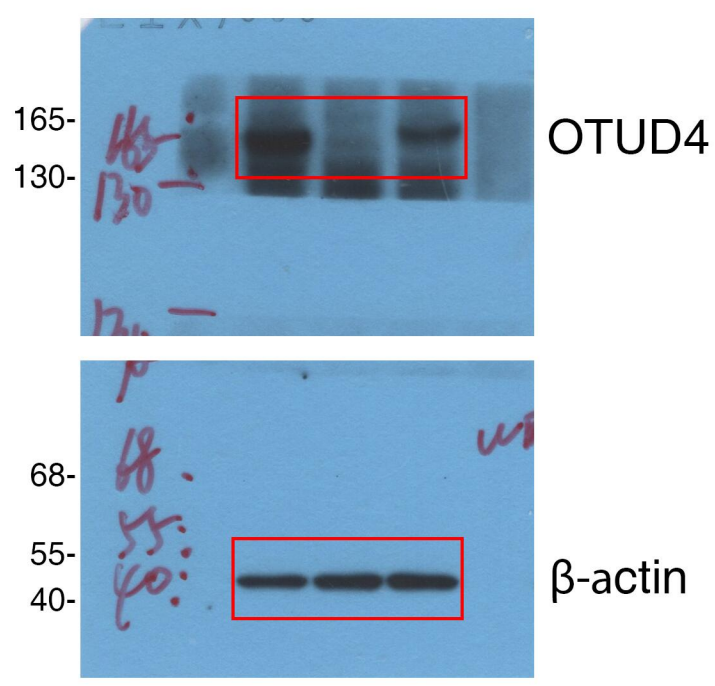

Figure 8G

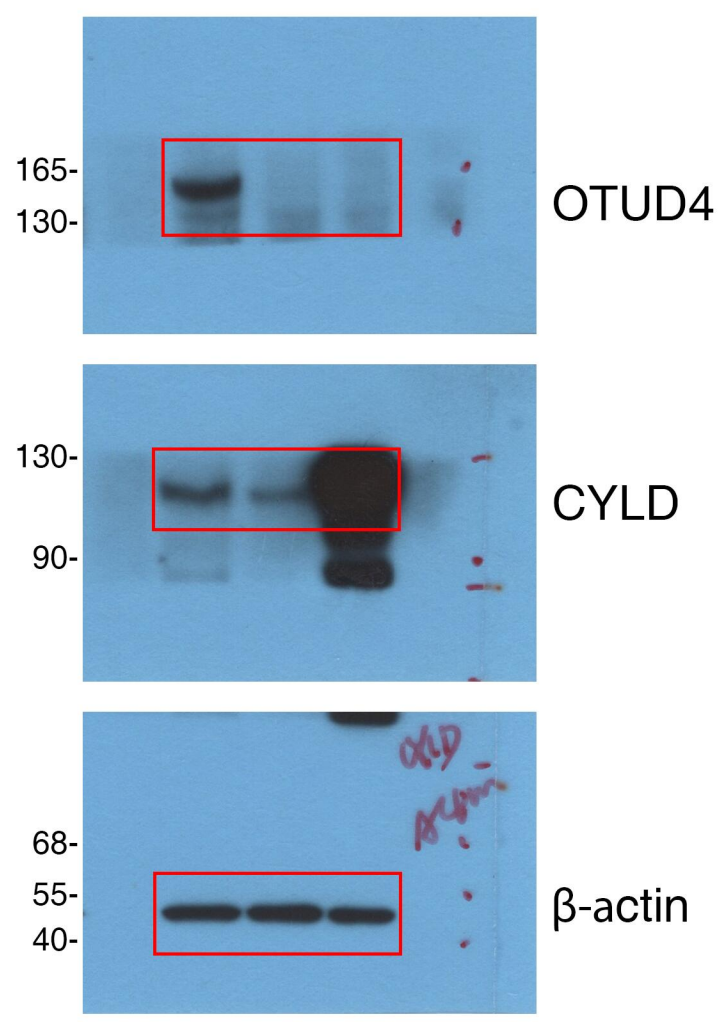

Figure S2A

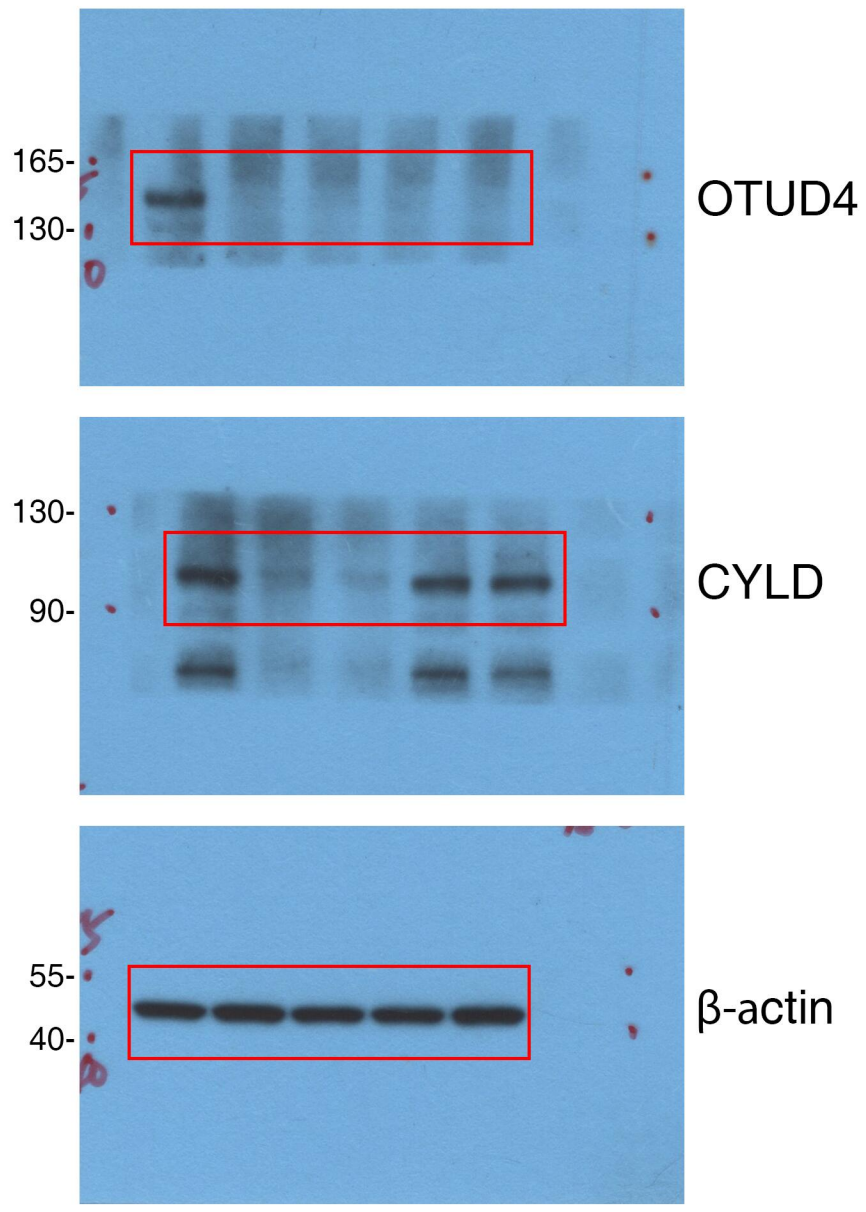

Figure S2B

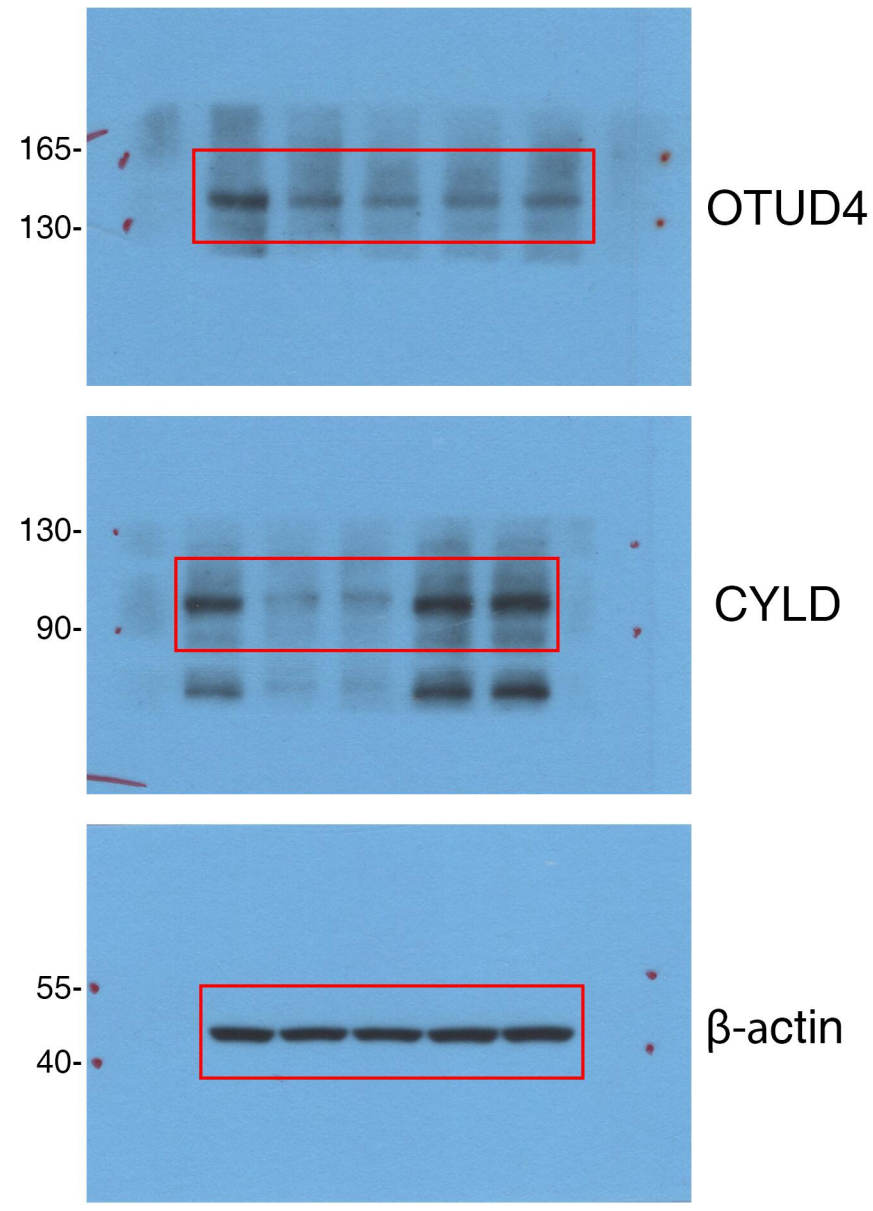

Figure S2C

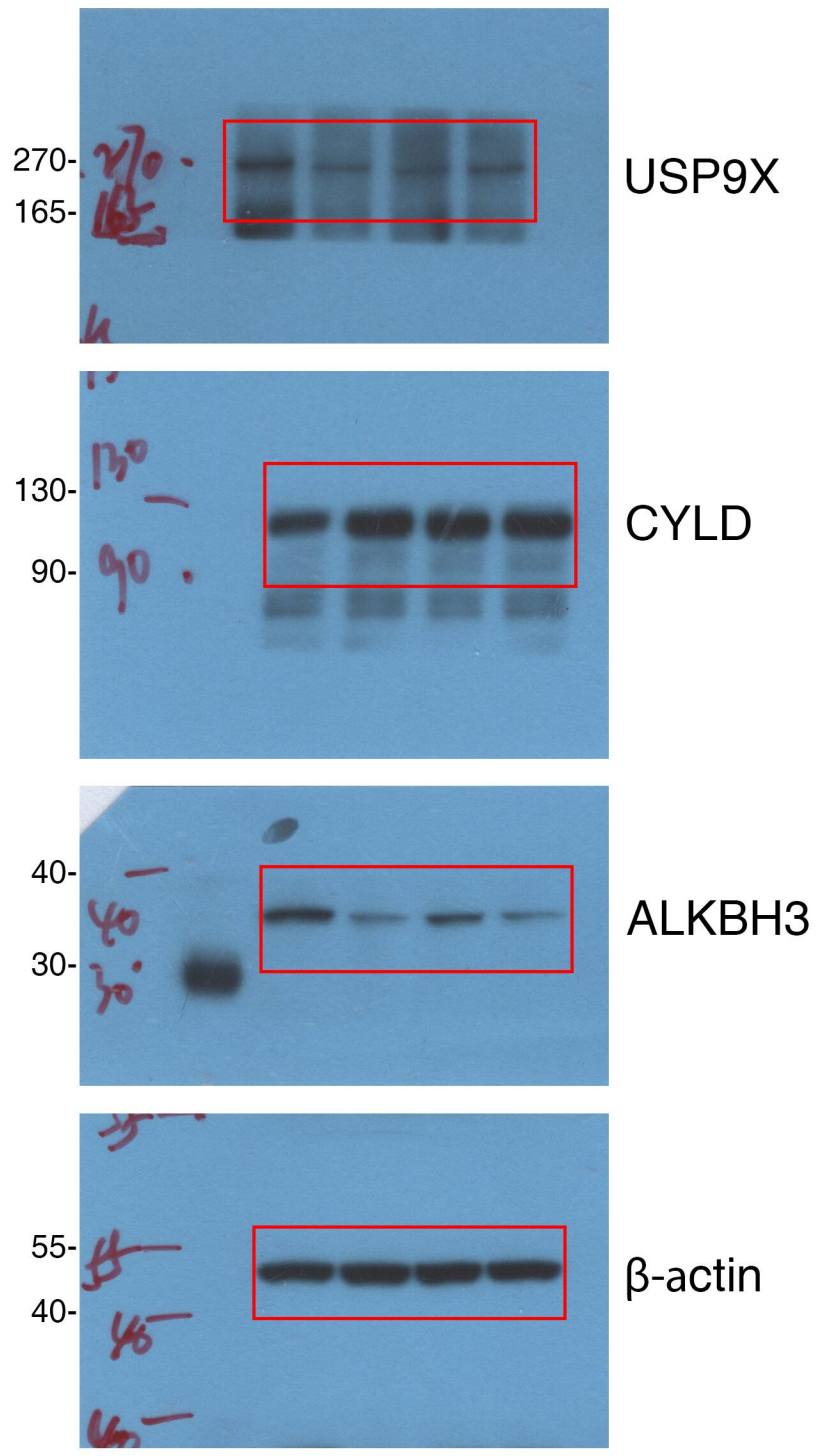

Figure S2D

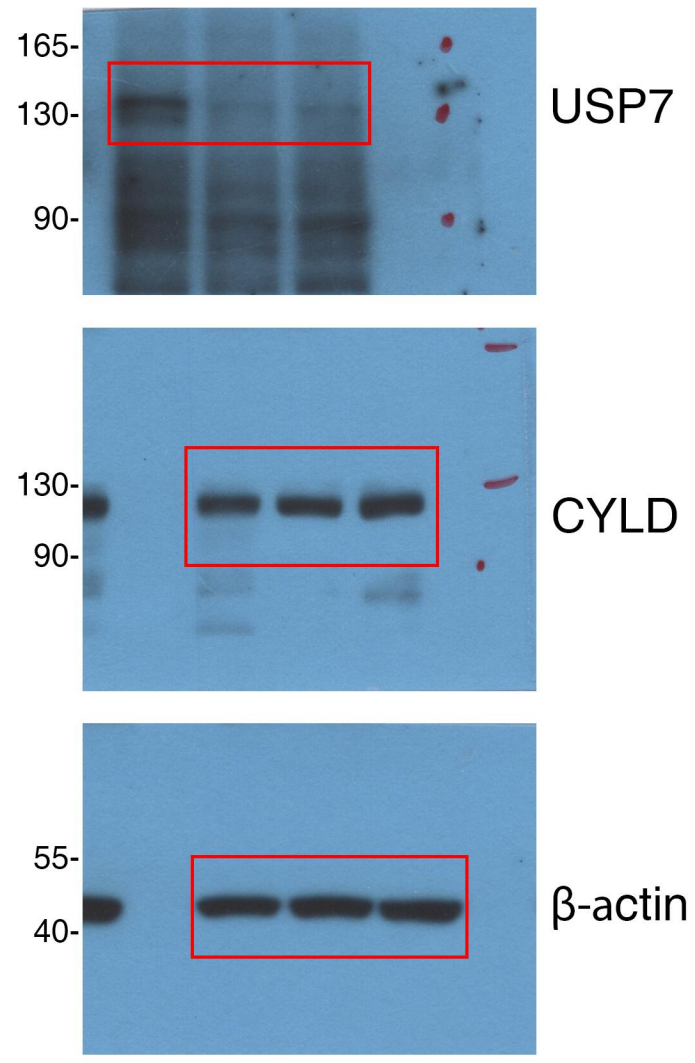

Figure S2E

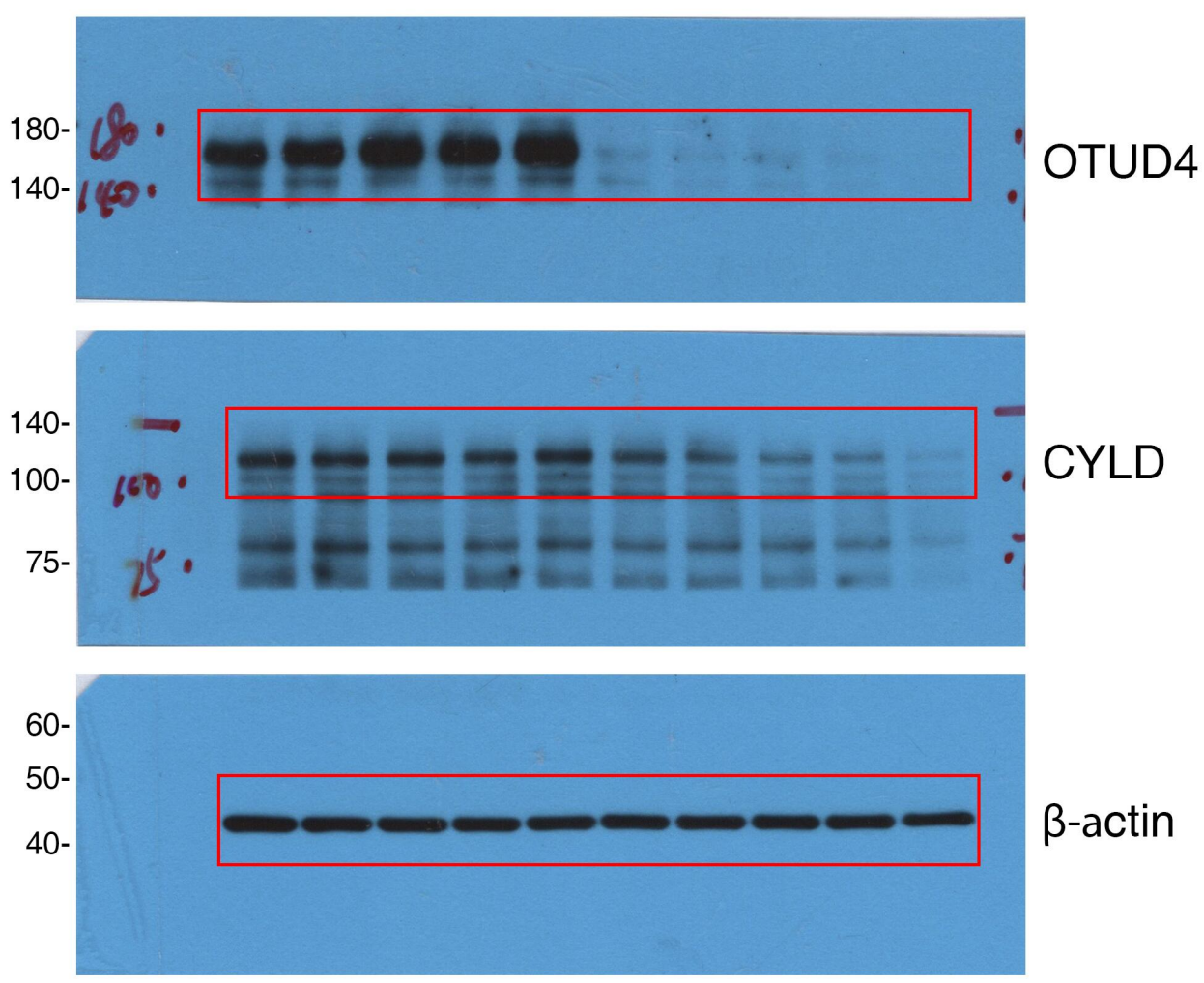

Figure S2F

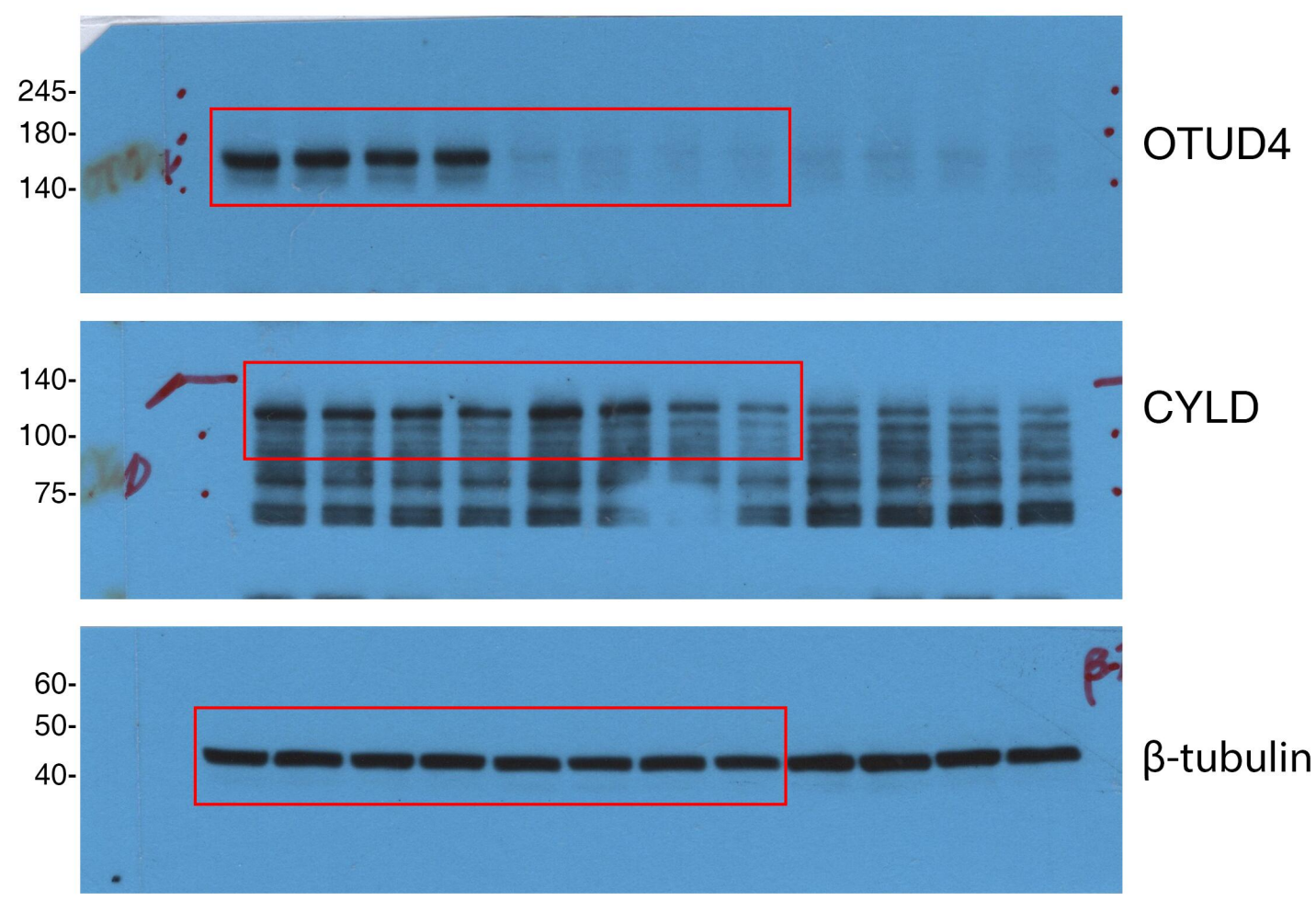

Figure S3A

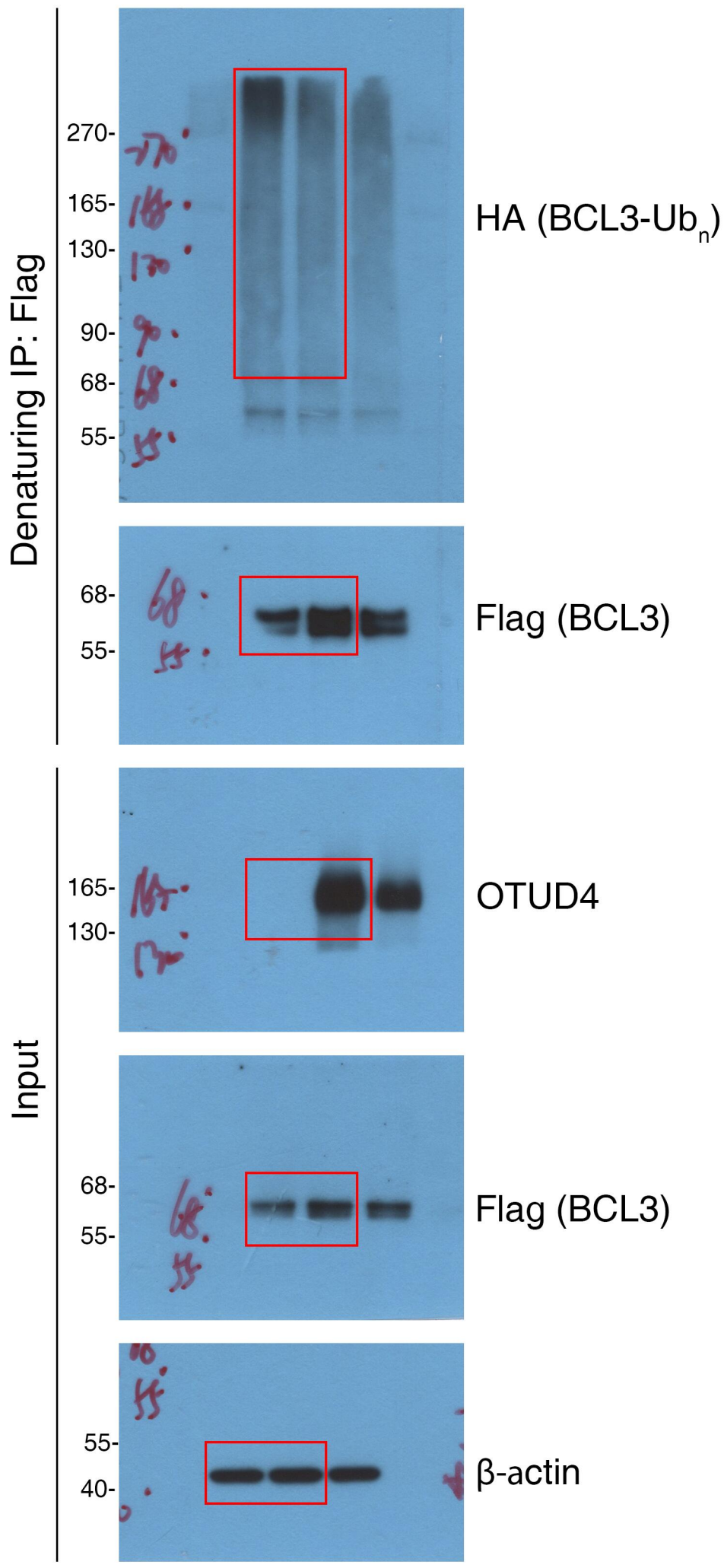

Figure S3B

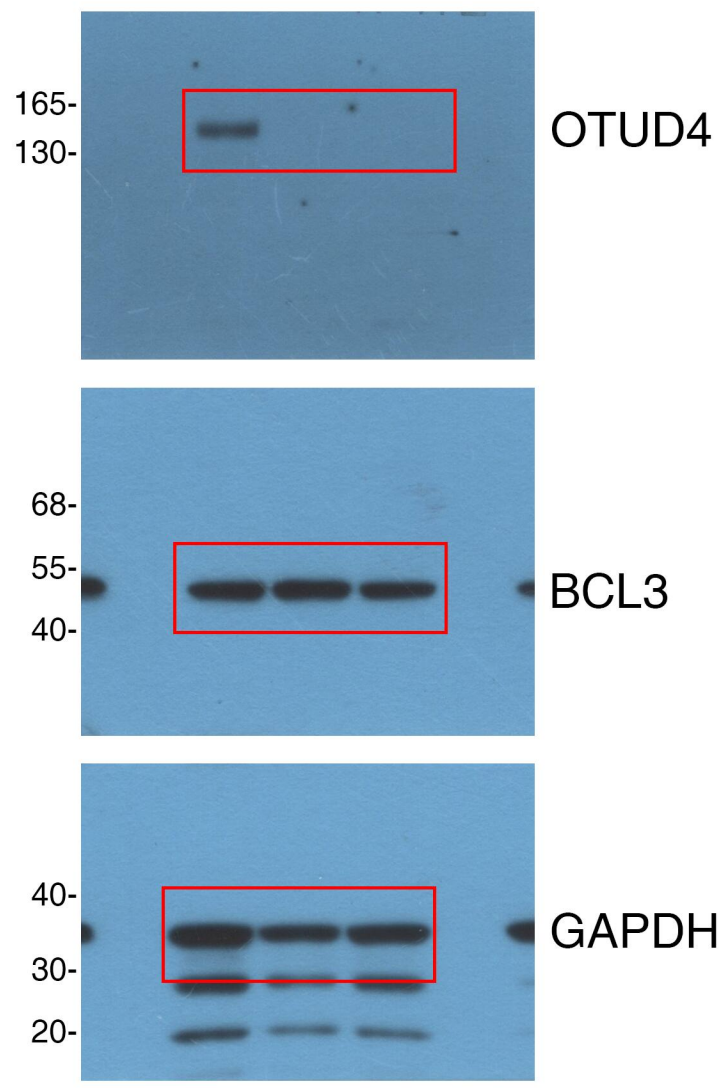

Figure S3C

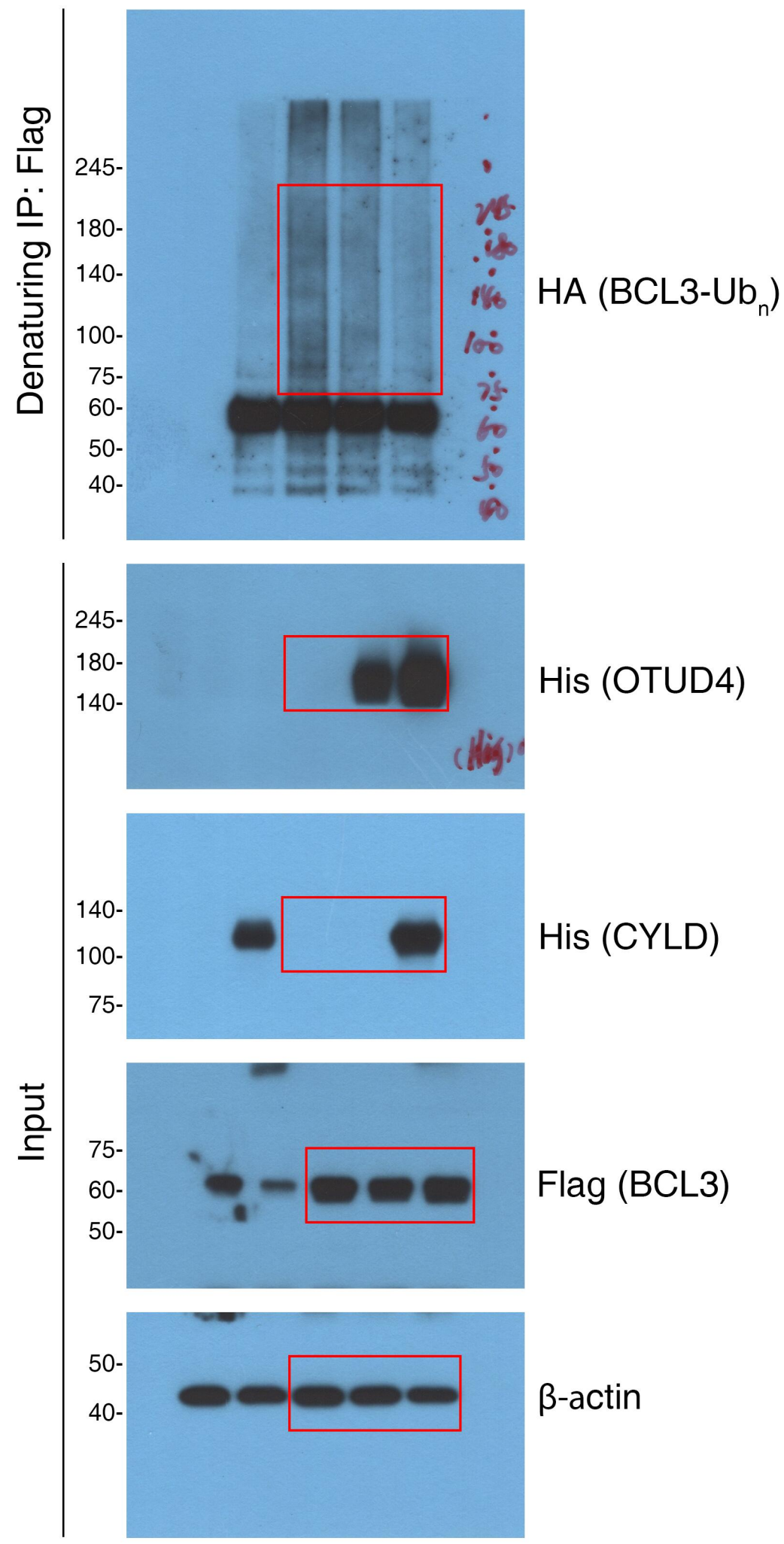

Figure S4A

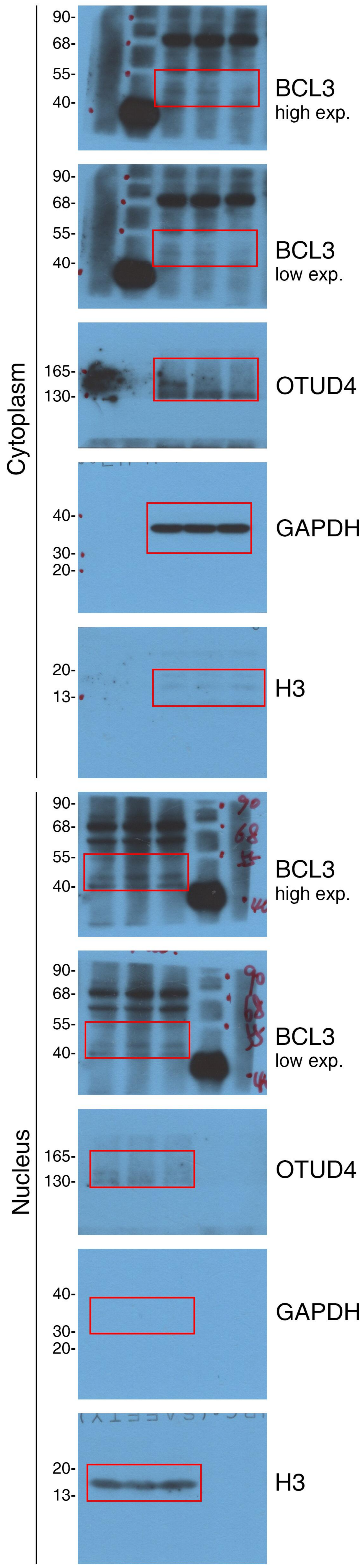

Figure S4B

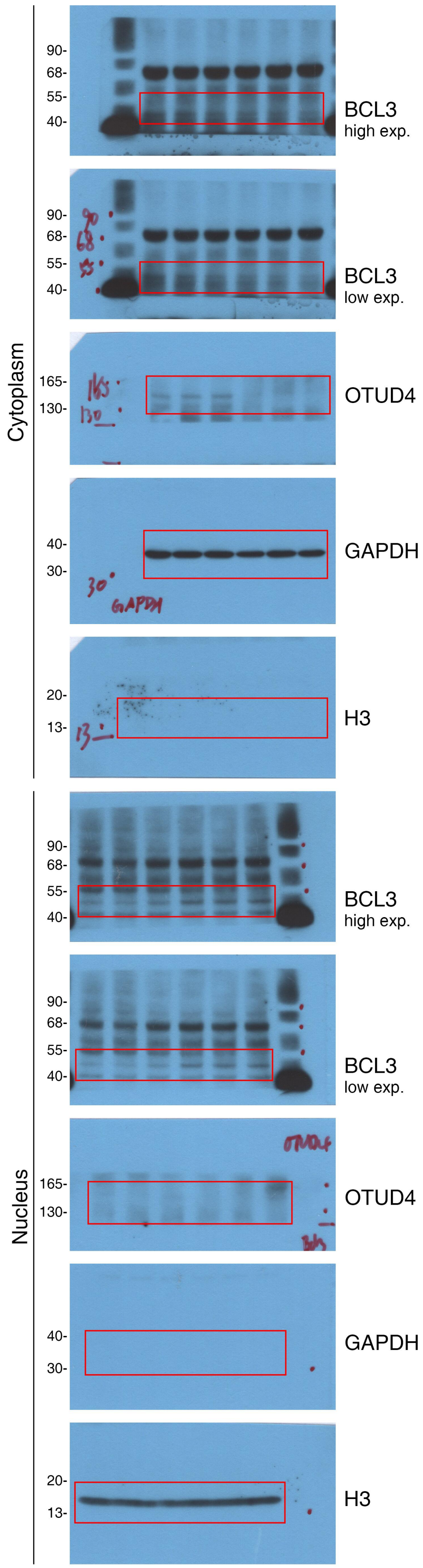

Figure S4C

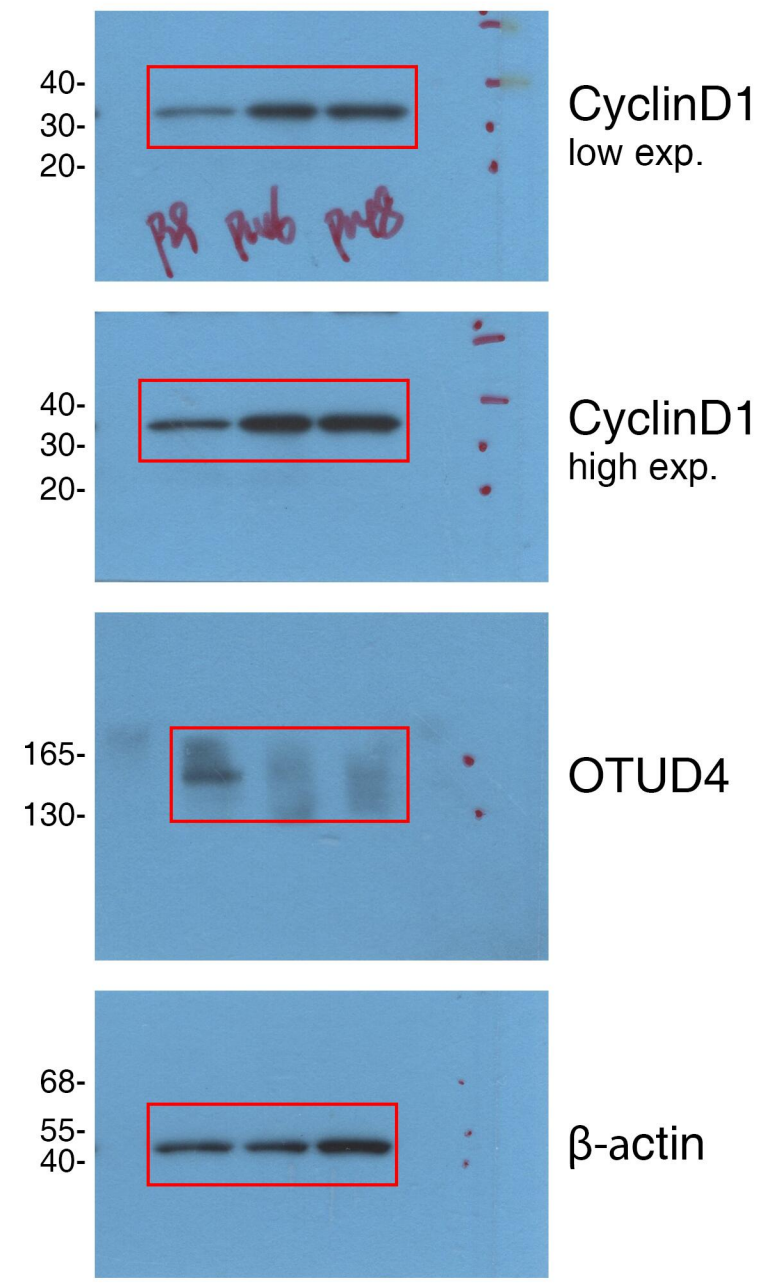

Figure S5B

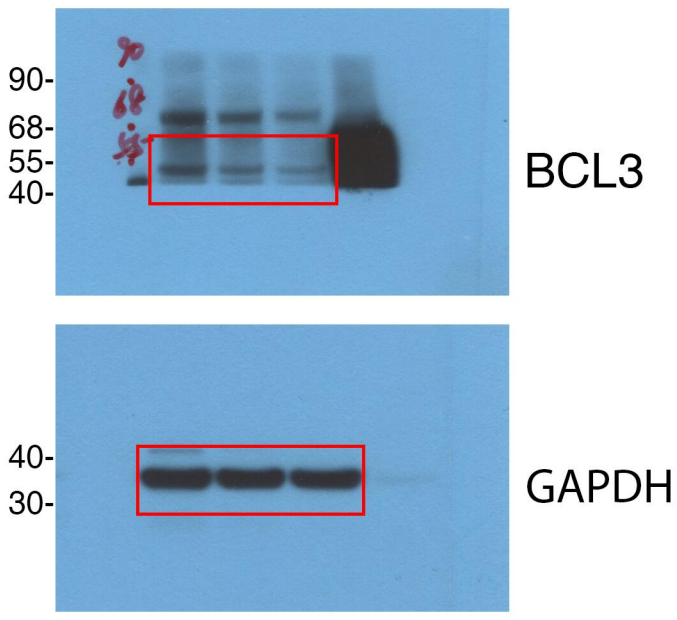

Figure S5E

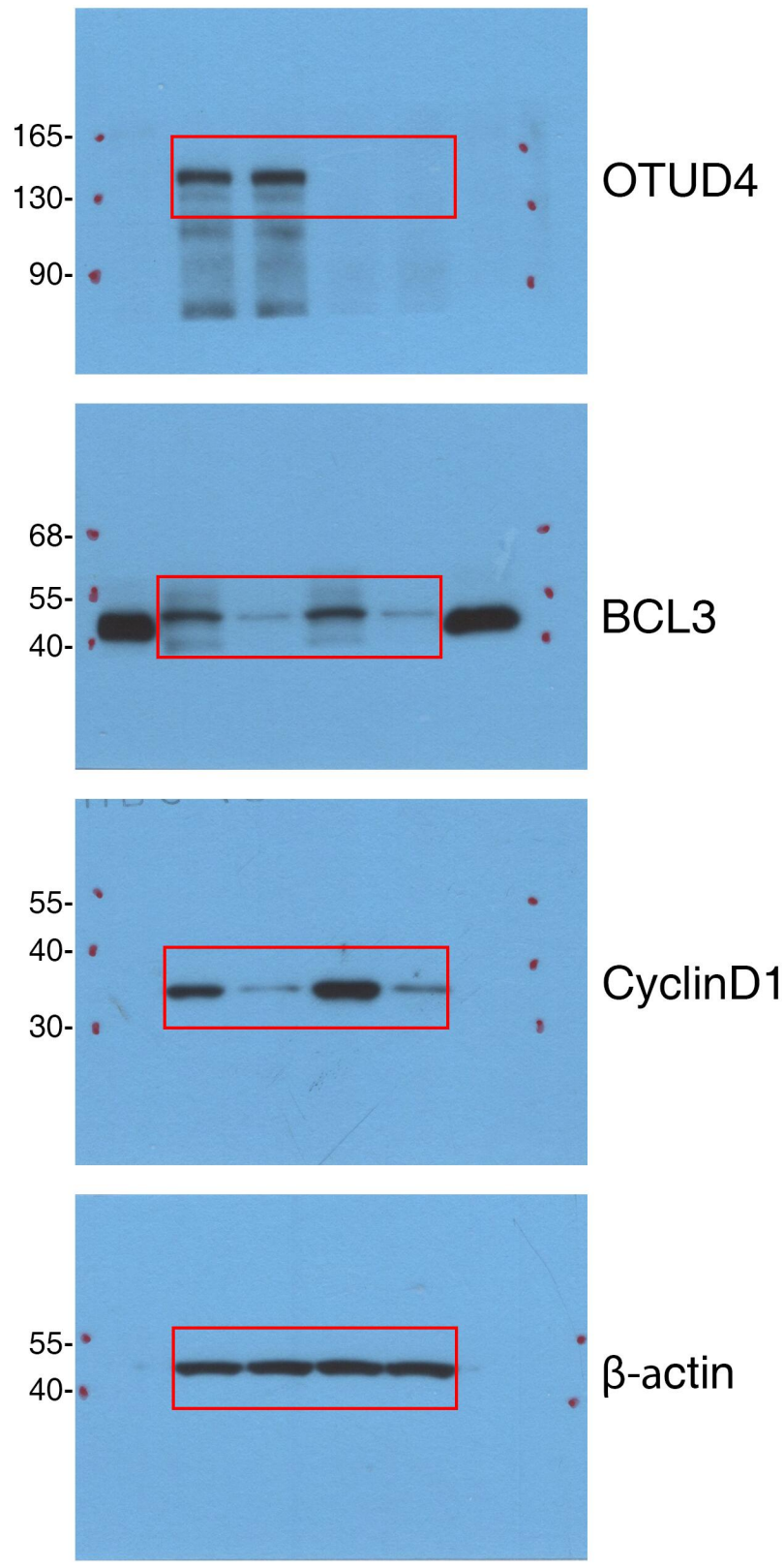

Supplement: Supplementary file 2 [file 41419_2026_9130_MOESM2_ESM.pdf]
